# Long Non-Coding RNA LINC01572 Promotes Hepatocellular Carcinoma Progression *via* Sponging miR-195-5p to Enhance PFKFB4-Mediated Glycolysis and PI3K/AKT Activation

**DOI:** 10.3389/fcell.2021.783088

**Published:** 2021-12-14

**Authors:** Shihui Lai, Zhipeng Quan, Yuesong Hao, Jun Liu, Zhiqian Wang, Luo Dai, Hongliang Dai, Songqing He, Bo Tang

**Affiliations:** ^1^ Department of Hepatobiliary Surgery, The First Affiliated Hospital of Guangxi Medical University, Nanning, China; ^2^ Key Laboratory of Basic and Clinical Application Research for Hepatobiliary Diseases of Guangxi, Nanning, China

**Keywords:** T2DM-related HCC, LINC01572, MiR-195-5p, PFKFB4, glycolysis

## Abstract

**Background:** Accumulating evidence indicates that type 2 diabetes mellitus (T2DM) is a risk factor for hepatocellular carcinoma (HCC), and T2DM-associated HCC represents a common type of HCC cases. We herein identify an lncRNA LINC01572 that was aberrantly upregulated in T2DM-related HCC *via* high-throughput screening. Based on this, the study was undertaken to identify the functional role and mechanism of LINC01572 in HCC progression.

**Methods:** RT-qPCR was used to detect the expressions of LINC01572 in HCC tissues and cell lines. Gain- or loss-of-function assays were applied to evaluate the *in vitro* and *in vivo* functional significance of LINC01572 in the HCC cell proliferation, migration, and invasion using corresponding experiments. Bioinformatics, RIP, RNA pull-down, and luciferase reporter assays were performed to explore the regulatory relationship of the LINC01572/miR-195-5p/PFKFB4 signaling axis.

**Result:** In this study, we profiled lncRNAs in HCC tissues and corresponding adjacent tissues from HCC patients with T2DM by RNA sequencing. Our data showed that LINC01572 was aberrantly upregulated in HCC tissues as compared with control, especially in those with concurrent T2DM. The high level of LINC01572 was correlated with advanced tumor stage, increased blood HbA1c level, and shortened survival time. The overexpression of LINC01572 significantly promoted HCC cell proliferation, migration, invasion, and epithelial-to-mesenchymal transition (EMT), while the knockdown of LINC01572 had the opposite effects on HCC cells. A mechanistic study revealed that LINC01572-regulated HCC progression *via* sponging miR-195-5p to increase the level of PFKFB4 and subsequent enhancement of glycolysis and activation of PI3K-AKT signaling.

**Conclusion:** LINC01572 acts as ceRNA of miR-195-5p to restrict its inhibition of PFKFB4, thereby enhancing glycolysis and activates PI3K/AKT signaling to trigger HCC malignancy.

## Introduction

Hepatocellular carcinoma (HCC) is the most common type in primary liver cancer and has extremely poor prognosis in spite of continuous progress of treatment strategies due to a high recurrence rate and difficult recognition in early stages. As such, HCC has ranked the second leading cause of cancer-related deaths globally (Sun et al., 2020; Ge et al., 2021). Hepatitis B virus (HBV) and hepatitis C virus (HCV) infection remain the leading causes of HCC incidence. Notably, accumulating evidence in recent years shows that type 2 diabetes mellitus (T2DM) is also closely associated with the increased risk of various malignancies, including HCC (Wild, 2011; Singh et al., 2018). Due to increased prevalence of unhealthy lifestyles, the incidence of T2DM has increased at an alarming rate, and at present, up to 9% of the world population is afflicted by this condition. This figure is projected to rise to 12% in 2025 if it continues like this (Jin and Liu, 2021), which would potentially contribute to the increased incidence of HCC. Based on this, further understanding the pathogenesis of T2DM-related HCC would help develop new diagnostic markers and therapeutic targets so as to clinically improve the therapeutic efficiency and prognosis.

Long non-coding RNAs (lncRNAs) is open reading frame (ORF)–free RNA molecules with a length >200 nucleotides. Although initially, this class of molecules was considered non-sense components for the organism, recent years have seen the critical role of lncRNAs in a variety of human diseases, including HCC, which implies that lncRNAs might become potentially efficient HCC diagnostic and therapeutic biomarkers (Chen et al., 2018; Zhao et al., 2019; Teng et al., 2020). Searching and identification of valuable HCC-related lncRNAs have emerged as a promising HCC research perspective (Hu et al., 2021; Mohan et al., 2021).

Long intergenic non-coding RNA 01572 (LINC01572) is a newly identified lncRNA molecule, with extremely limited information available regarding its role in cancer progression, although it has been revealed to be differentially expressed in lung squamous cancer (Chen et al., 2017) and regulate cisplatin resistance in gastric cancer (Song et al., 2020). Whether LINC01572 regulates HCC, fate is completely unclear. In the present study, we found that LINC01572 was upregulated in HCC tissues, those from patients complicated with T2DM in particular. On the basis of this, we then further analyzed its biological significance in HCC progression and also explored the possible molecular mechanism, in an attempt to provide a potentially effective diagnostic and therapeutic target for HCC, especially those complicated by T2DM.

## Materials and Methods

### Chemicals and Antibodies

TRIzol reagents were obtained from Invitrogen (Grand Island, NY, United States). Antibodies against E-cadherin, N-cadherin, vimentin, and *β*-catenin were purchased from Cell Signaling Technology (Danvers, MA, United States), and those against *β*-actin and PFKFB4 were purchased from Abcam (Cambridge, MA, United States). All other chemicals were purchased from Sigma-Aldrich unless otherwise stated (St. Louis, MO, United States).

### Patient Samples and Cell Lines

Seventy pairs of HCC tumor and corresponding adjacent normal tissues were collected from HCC patients at The First Affiliated Hospital of Guangxi Medical University. These tissues were placed in liquid nitrogen immediately after surgical resection and then transferred to −80°C for later use. All enrolled patients have provided written informed consent, and the study was reviewed and approved by the Ethics Committee of Guangxi Medical University.

Human HCC cell lines SK-hep1, SNU-449, SMMC-7721, HCC-LM3, Huh7, and MHCC-97H were purchased from ATCC (American Type Culture Collection) or the Institute of Biochemistry and Cell Biology (Chinese Academy of Sciences, Shanghai, China). SK-hep1, SNU-449, and SMMC-7721 were cultured in the RPMI-1640 medium containing 10% fetal bovine serum (FBS, Gibco), 1% penicillin, and streptomycin. Other cells were cultured in DMEM containing 10% fetal bovine serum (Gibco), 1% penicillin, and streptomycin. All the cells were subcultured in a 37°C incubator with 5% CO_2_.

### Plasmid Construction and Cell Transfection

HCC cells were inoculated into six-well plates and used for *in vitro* transfection when the cell density reached 70∼80% using Lipofectamine 3000 (Thermo Fisher Scientific). The cells were transfected with pcDNA3.1 (+) vectors expressing LINC01572, LINC01572 short hairpin RNA (shRNA), or PFKFB4, miR-195-5p mimics, and PFKFB4 small interference RNA (siRNA) and their corresponding controls (GenePharma, Shanghai, China) for 48 h. shLINC01572 was transfected into hepatocellular carcinoma cell SNU-449 using lentivirus in mouse xenograft.

### Reverse Transcription Quantitative Polymerase Chain Reaction (RT-qPCR)

An RNA Simple Total RNA Kit (TIANGEN, DP419) was used to extract total RNA from tissues and cultured cells according to the manufacturer’s instructions. The extracted mRNA and miRNA were then reversely transcribed into cDNA using a RevertAid First-Strand cDNA Synthesis Kit (Thermo Scientific, #K1622) and miRcute Plus miRNA First-Strand cDNA Kit (TIANGEN, Beijing, China), respectively. Subsequently, iTaq Universal SYBR^®^ Green Supermix (Bio-RAD, United States) and miRcute Plus miRNA qPCR Kit (SYBR, TIANGEN, Beijing, China) were used for RT-qPCR amplification. The RNA level was quantified using the 2^−ΔΔCt^ method.

### Western Blotting

Total protein was extracted from cell lysate and quantified by using the BCA method. Subsequently, an equal amount of protein was subjected to polyacrylamide gel electrophoresis (SDS-PAGE). Separated protein was then transferred to the polyvinylidene difluoride (PVDF) membrane. The membrane was blocked with 5% non-fat milk for 1 h at room temperature and then incubated with primary antibodies and horseradish peroxide (HRP)–labeled secondary antibodies. Bound antibodies were detected using enhanced chemiluminescence (ECL).

### Cell Counting Kit-8 (CCK-8)

The cells were seeded into 96-well plates and cultured at 37°C for 0∼96 h. Subsequently, 10 μL CCK-8 reaction reagent (Dojindo, Japan) was then added to each well, and after 2 h of incubation at 37°C, the absorbance value was detected at a wavelength of 450 nm.

### Colony Formation Assay

The cells were seeded in 6-well plates and allowed to grow for 10∼14 days at 37°C. At the end of the growth, cell colonies were fixed with 70% methanol and stained by crystal violet solution (Solarbio) for 30 min. The clones containing more than 50 cells were counted for analysis.

### Transwell Assay

Transwell assay was conducted to evaluate the migration and invasion of HCC cells. In the invasion assay, the chamber bottom was pre-coated with Matrigel, whereas in the migration assay, no Matrigel was applied. The cells suspended in a serum-free medium were seeded onto transwell inserts. After 24∼48 h, the migrated and invasive cells were fixed with paraformaldehyde and stained with crystal violet for visualization.

### Luciferase Activity Assay

The wild-type and mutant-type luciferase plasmids for LINC01572 or PFKFB4 were constructed using PmiRGLO dual-luciferase reporters. The constructed plasmid was co-transfected into SNU-449 and HCC-LM3 cells with miR-195-5p mimics or NC mimics. A dual-luciferase reporter assay system (Promega, Madison, United States) was used to determine the luciferase activity.

### RNA Immunoprecipitation (RIP) Assay

RIP assay was performed using a Magna RIP™ RNA-Binding Protein Immunoprecipitation Kit (Millipore, United States) according to the manufacturer’s instructions. The cell extracts were used for immunoprecipitation of RNA with beads conjugated with antibodies against AGO2. The proteins in the complex were removed by 0.1% SDS/protease K (0.5 mg/ml) at 55°C for 30 min. Immunoprecipitated LINC01572 and miR-195-5p were detected by RT-qPCR.

### RNA Pull-Down

LINC01572 was labeled using a Pierce^TM^ RNA 3 End Desthiobiotinylation Kit (Thermo, 20163) for the attachment to streptavidin magnetic beads. The RNA pull-down assay was performed using a Thermo Scientific Pierce Magnetic RNA-Protein pull-down Kit (Thermo, 20164), followed by the AGO2 analysis by Western blot, and miR-195-5p by RT-qPCR.

### Statistical Analysis

Data were expressed as mean ± standard deviation (SD) for at least three independent experiments, and GraphPad Prism 8.0 (GraphPad Software Inc., San Diego, CA, United States) was used for plotting and statistical analysis. Student’s t test and one-way analysis of variance (ANOVA) were performed for comparison between groups. Pearson’s correlation analysis was used for identifying the correlation between gene expressions. *p* < 0.05 was considered statistically significant.

## Result

### LINC01572 Was Highly Expressed in Hepatocellular Carcinoma and Associated With Poor Prognosis

**FIGURE 1:**
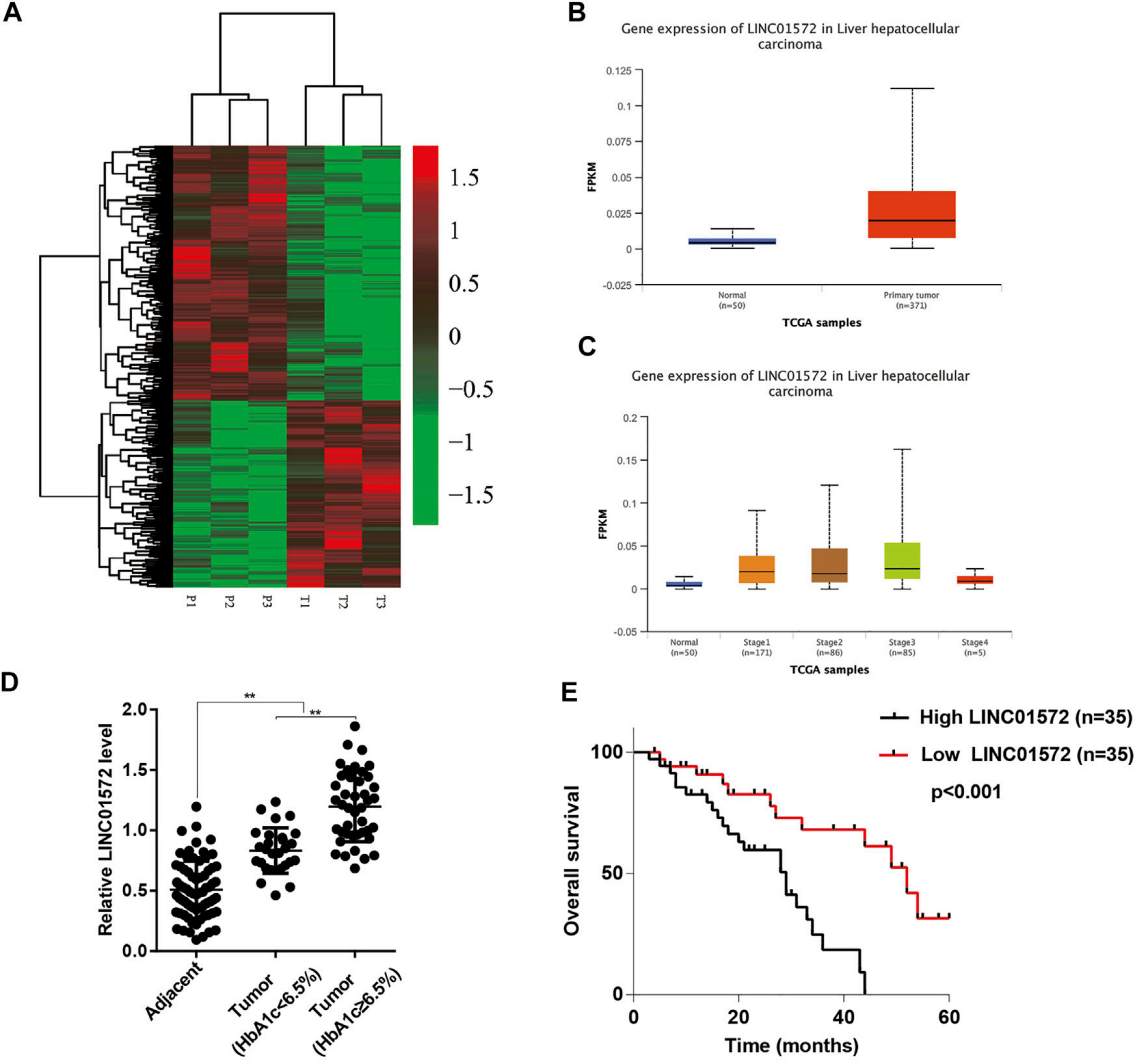
LINC01572 is highly expressed in HCC and is associated with poor prognosis. **(A)** Heatmap of the differential expression of LncRNA in cancer tissues and corresponding adjacent tissues from HCC patients with concurrent T2DM. **(B)** Comparison of LINC01572 expression in HCC (*n* = 371) and normal liver (*n* = 50) tissues based on TCGA dataset. **(C)** Analysis of LINC01572 expression in HCC samples at different stages based on TCGA dataset. **(D)** RT-qPCR analysis of LINC01572 expression in HCC tissues and adjacent normal tissues, in which HCC samples were subgrouped based on blood HbA1c level. **(E)** Kaplan–Meier analysis of survival time based on the LINC01572 expression in 70 HCC patients. ^**^ *p* < 0.01.

To identify that the lncRNAs were potentially involved in T2DM-related HCC progression, transcriptome sequencing was performed on three pairs of tumor and adjacent tissue samples from T2DM–HCC patients. The results showed that LINC01572 lied in the forefront among the differentially expressed lncRNAs (Figure 1A). In line with this, TCGA dataset showed that LINC01572 is upregulated in HCC samples as compared with adjacent non-cancerous tissues (Figure 1B), and further, its expression was correlated with the advanced HCC stage (Figure 1C). In addition, the RT-qPCR assay revealed that LINC01572 was further elevated in high-blood HbA1c subgroup (Figure 1D). Moreover, LINC01572 expression was significantly correlated with advanced tumor stage and HbA1c level (Table 1). In particular, the Kaplan–Meier survival analysis data indicated that the higher level of LINC01572 was associated with shorter survival time in HCC (*p* < 0.001) (Figure 1E). These data suggest that LINC01572 might play a key role in T2DM-related HCC progression.

**TABLE 1:** Relationship between LINC01572 and clinicopathological parameters in 70 HCC patients.

Variable	All cases	LINC01572 expression		*p*
		Low (*n* = 35)	High (*n* = 35)	
Age (years)				
< 50	28	11	17	—
≥ 50	42	24	18	0.1432
Gender				
Male	48	23	25	—
Female	22	12	10	0.6066
Tumor nodule number				
Solitary	30	17	13	—
Multiple (≥ 2)	40	18	22	0.3340
HBV infection				
Positive	44	21	23	—
Negative	26	14	12	0.6208
Tumor size (cm)				
< 5	46	22	24	—
≥ 5	24	13	11	0.6146
TNM stage				
Ⅰ-Ⅱ	31	22	9	—
Ⅲ-Ⅳ	39	13	26	0.0018
Serum AFP (μg/L)				
≤ 200	43	24	19	—
> 200	27	11	16	0.2196
HbA1c (%)				
< 6.5	28	20	8	—
≥ 6.5	42	15	27	0.0147

### LINC01572 Affected Proliferation, Migration, and Invasion of Hepatocellular Carcinoma Cells

**FIGURE 2:**
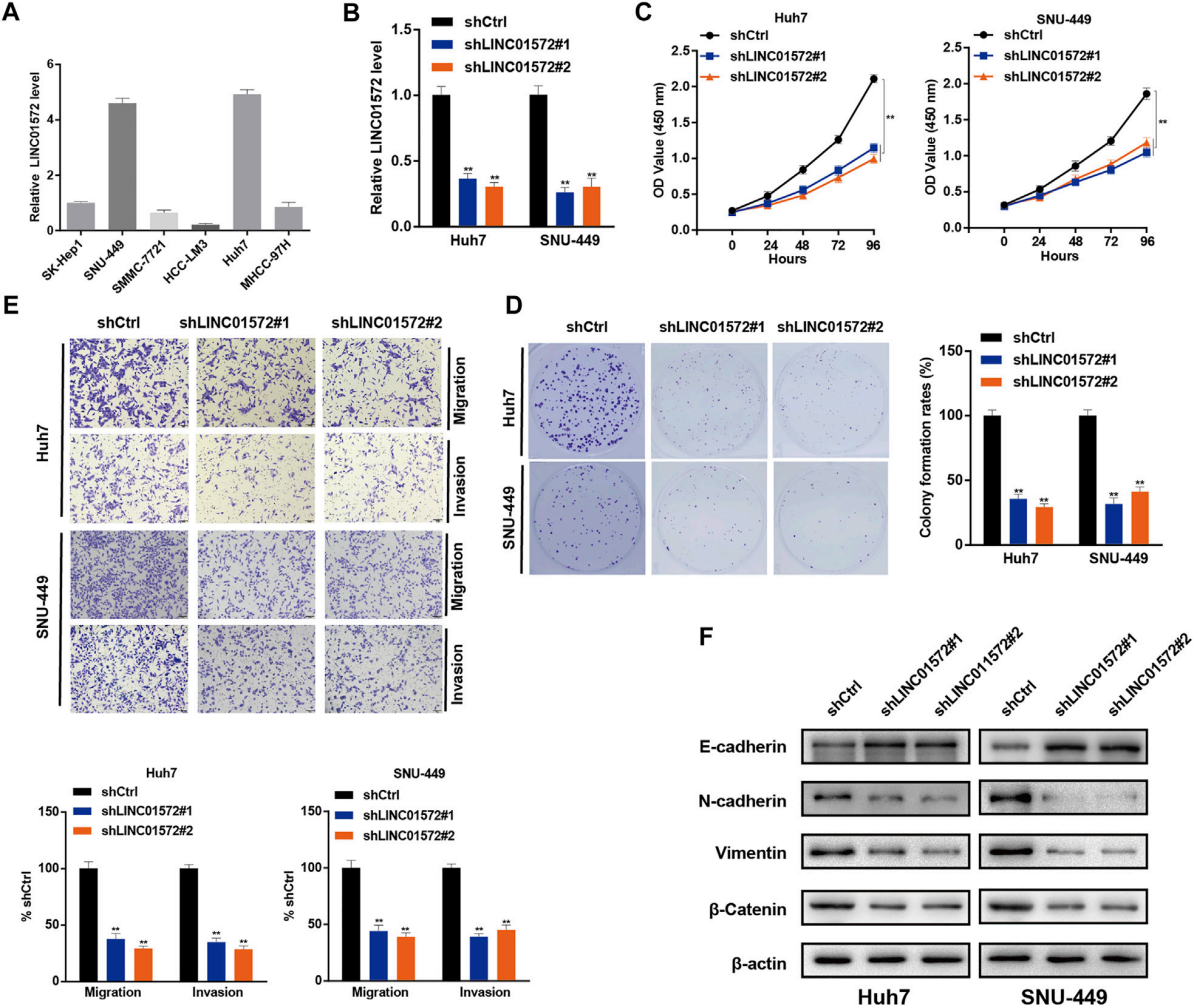
LINC01572 silencing inhibited proliferation, migration, and invasion of HCC cells. **(A)** RT-qPCR analysis of LINC01572 expression in six HCC cell lines. **(B)** RT-qPCR analysis of the suppressive effect of specific shRNA on the LINC01572 level. **(C,D)** Effect of LINC01572 knockdown on Huh7 and SNU-449 cell proliferation. **(E,F)** Effect of LINC01572 knockdown on Huh7 and SNU-449 cell migration, invasion, and EMT marker expression. All data are shown as mean ± standard deviation (*n* = 3). ^**^ *p* < 0.01.

**FIGURE 3:**
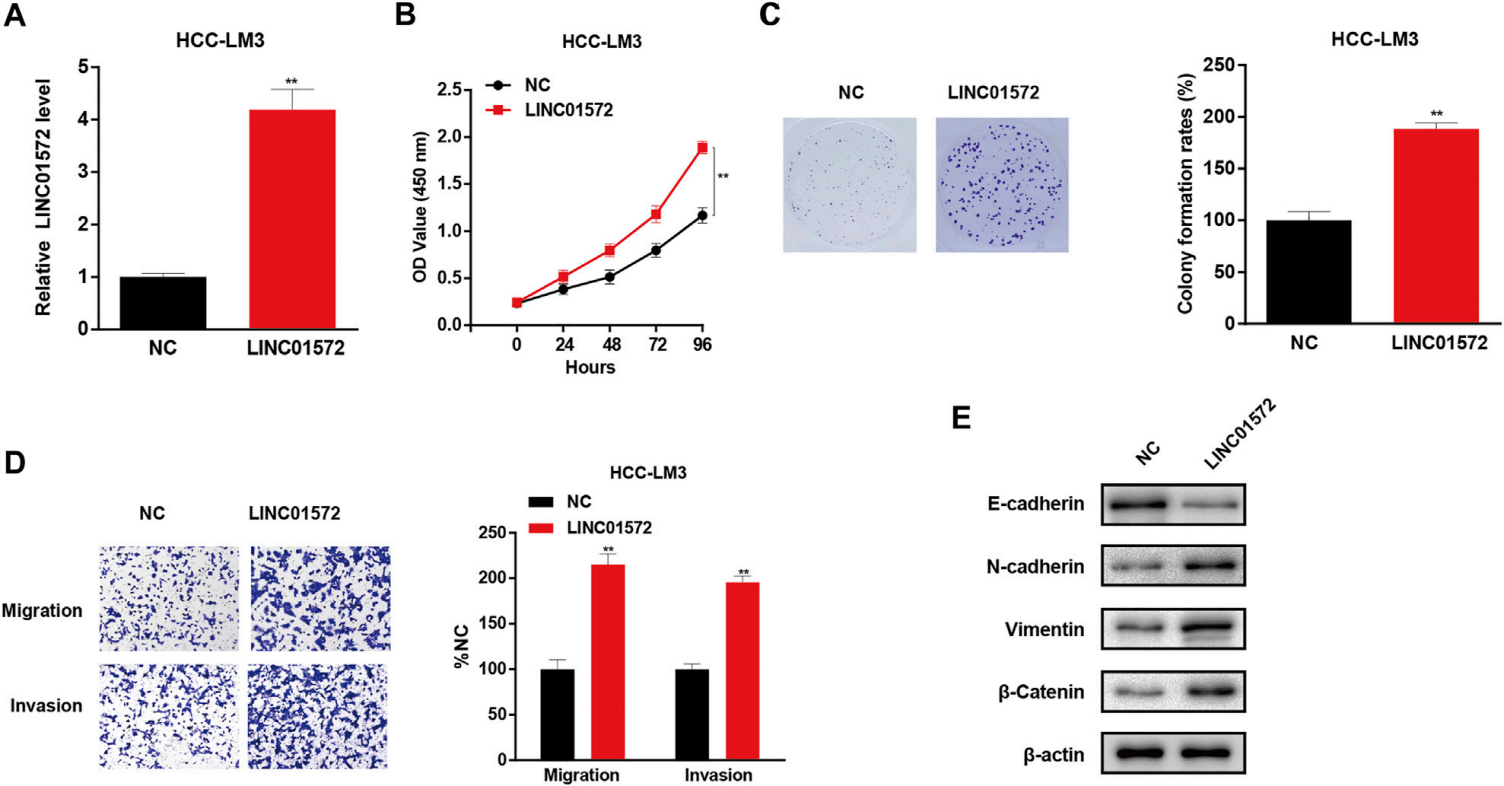
LINC01572 overexpression promoted the proliferation, migration, and invasion of HCC cells. **(A)** RT-qPCR analysis of LINC01572 level in LINC01572 overexpressed HCC-LM3 cells. **(B,C)** Effect of LINC01572 overexpression on HCC cell proliferation. **(D,E)** Effect of LINC01572 overexpression on HCC cell migration, invasion, and EMT marker expression. All data are shown as mean ± standard deviation (*n* = 3). ^**^ *p* < 0.01.

In order to identify the role of LINC01572 in HCC, Huh7 and SNU-449 cells were selected for loss-of-function assays and overexpression of LINC01572 in HCC-LM3 cells, according to the basal level of LINC01572 in HCC cell lines (Figure 2A). RT-qPCR showed that the LINC01572 level was significantly repressed upon shLINC01572 transfection and enhanced by LINC01572 overexpression (Figures 2B, 3A). Subsequently, cck-8 and colony formation assays showed that downregulation of LINC01572 remarkably decreased the growth rate of Huh7 and SNU-449 cells (Figures 2C,D), while overexpression significantly promoted the growth of HCC-LM3 cells (Figures 3B,C). Transwell assays revealed that migration and invasion were significantly prevented by shLINC01572 transfection in Huh7 and SNU-449 cells (Figure 2E) and enhanced in LINC01572 overexpressed HCC-LM3 cells (Figure 3D). Furthermore, the enhanced E-cadherin and decreased N-cadherin, vimentin, and *β*-catenin were also found with LINC01572 knockdown, whereas overexpression of LINC01572 in HCC-LM3 cells produced an opposite result (Figures 2F, 3E). The aforementioned results demonstrated that LINC01572 stimulates the proliferation, migration, and invasion of HCC cells *in vitro*.

### Silencing of LINC01572 Inhibits Hepatocellular Carcinoma Cell Growth and Distant Metastasis *In Vivo*

**FIGURE 4:**
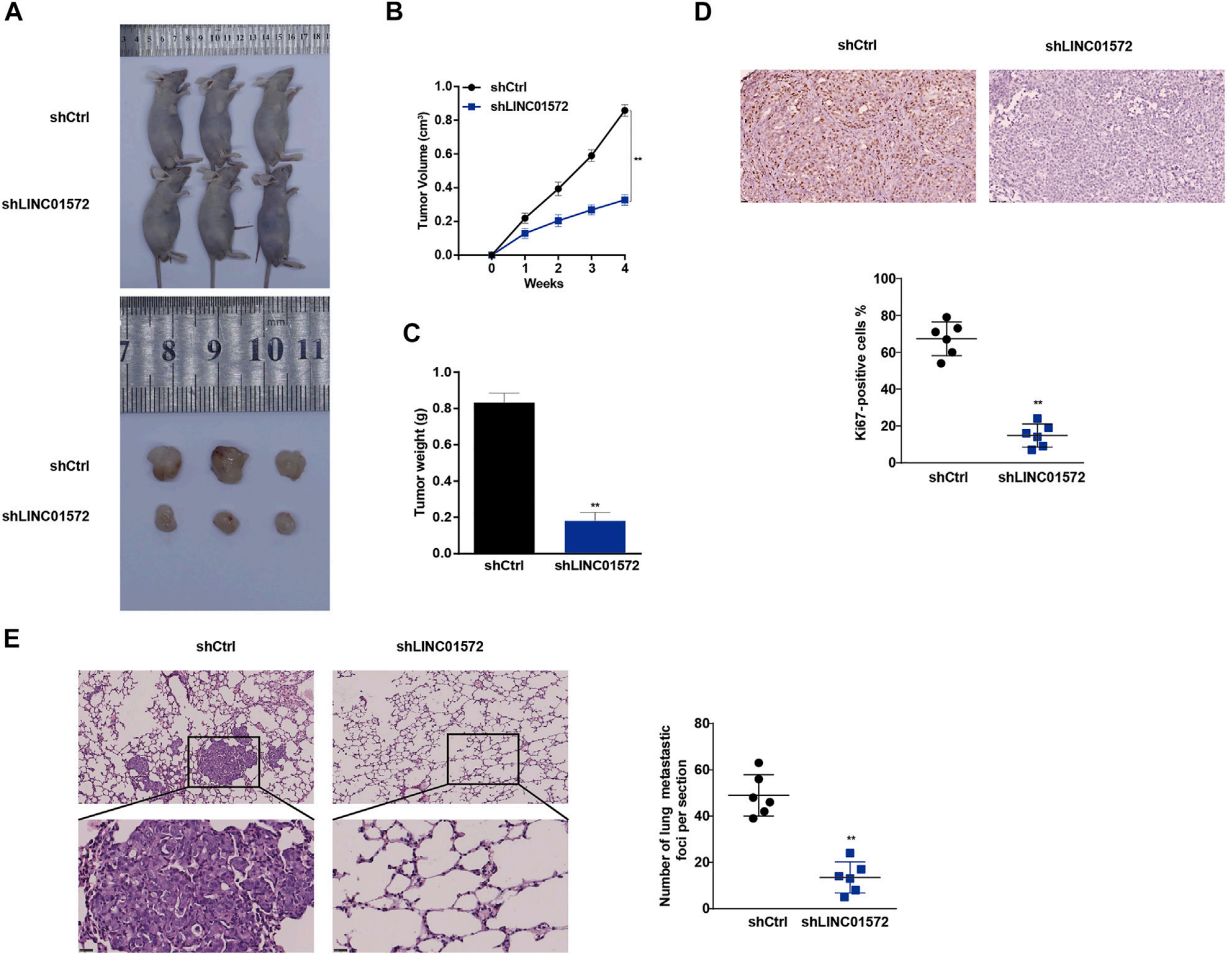
Silencing of LINC01572 inhibits the HCC cell growth and distant metastasis *in vivo*. **(A)** Representative image of tumors following injection of SNU-449-shLINC01572 or control cells **(B,C)** Xenograft tumor volume and weight in nude mice subcutaneously injected with SNU-449-shLINC01572 or control cells. **(D)** Ki67-positive cell numbers in the sections of xenograft tumors from nude mice injected with SNU-449-shLINC01572 or control cells. **(E)** Number of lung metastatic nodules following tail vein injection of SNU-449-shLINC01572 or control cells. All data are shown as mean ± standard deviation (*n* = 3). ^**^ *p* < 0.01.

In order to identify the role of LINC01572 *in vivo* in HCC progression, a mice xenograft model was established. As shown in Figures 4A–C, tumor volume and weight were significantly lower in the shLINC01572 group. Furthermore, it was observed that Ki67-positive cells in xenograft tumors were also reduced (Figure 4D). In addition, in the lung metastasis model, we observed that the number of lung metastatic nodules in the shLINC01572 group was significantly lower than that in the control group (Figure 4E). These results suggest that silencing LINC01572 restrains HCC growth and metastasis *in vivo*.

### LINC01572 Upregulates PFKFB4 Expression to Increase Glycolysis and Activate PI3K/AKT by Sponging miR-195-5p

**FIGURE 5:**
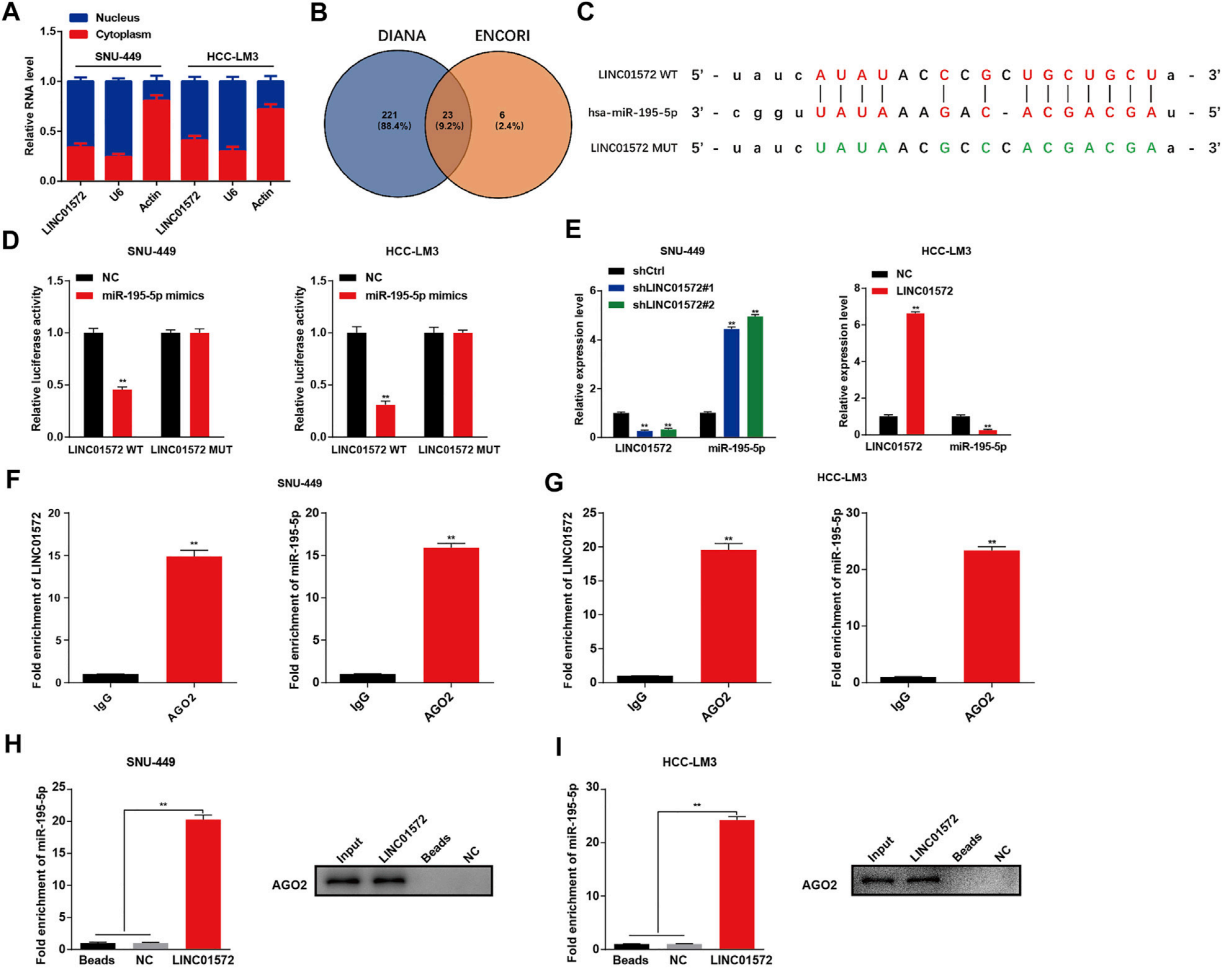
LINC01572 sponges and downregulates miR-195-5p in HCC cells. **(A)** Distribution of LINC01572 in nucleus and cytoplasm fraction was detected by RT-qPCR assay **(B)** DIANA Tool and ENCORI were used to predict the downstream target gene of LINC01572, and the intersection was obtained. **(C)** ENCORI was used to predict the binding sites of LINC01572 and miR-195-5p. **(D)** Target relationship of LINC01572 and miR-195-5p was verified by dual-luciferase assay **(E)** Relative expression of miR-195-5p after LINC01572 knockdown and overexpression. **(F,G)** RIP assay was used to detected the relative enrichment of LINC01572 and miR-195-5p in anti-IgG–specific or anti-AGO2–specific immunoprecipitates. **(H,I)** RNA pull-down assay showing the interaction between LINC01572 and miR-195-5p. All data are shown as mean ± standard deviation (*n* = 3). ^**^ *p* < 0.01.

To understand the mechanism underlying LINC01572-mediated HCC malignant behavior, the subcellular location of LINC01572 was analyzed using the nuclear/cytosol fraction assay. It was found that a considerable proportion of LINC01572 was distributed in the cytoplasm of HCC cells (Figure 5A). We thus reckoned that LINC01572 might exert its biological function in HCC *via* a ceRNA mechanism. Using DIANA Tools and ENCORI, we obtained 23 miRNAs that could potentially bind to LINC01572 (Figure 5B). Among others, it has been reported that miRNA-195-5p acts as a suppressor in HCC (Xu et al., 2015). Therefore, miRNA-195-5p was selected for the subsequent study subsequently. The potential biding between LINC01572 and miR-195-5p was predicted by ENCORI (Figure 5C). Next, the binding between LINC01572 and miR-195-5p was confirmed by the dual-luciferase assay (Figure 5D). RT-qPCR data revealed that LINC01572 negatively modulated the expression of miR-195-5p (Figure 5E). The RIP assay showed a significantly increased enrichment of LINC01572 and miR-195-5p in the AGO2 precipitate compared with the IgG control (Figures 5F,G). In line with this, the RNA pull-down assay showed a significantly increased binding of AGO2 and miR-195-5p to LINC01572 (Figures 5H,I).

**FIGURE 6:**
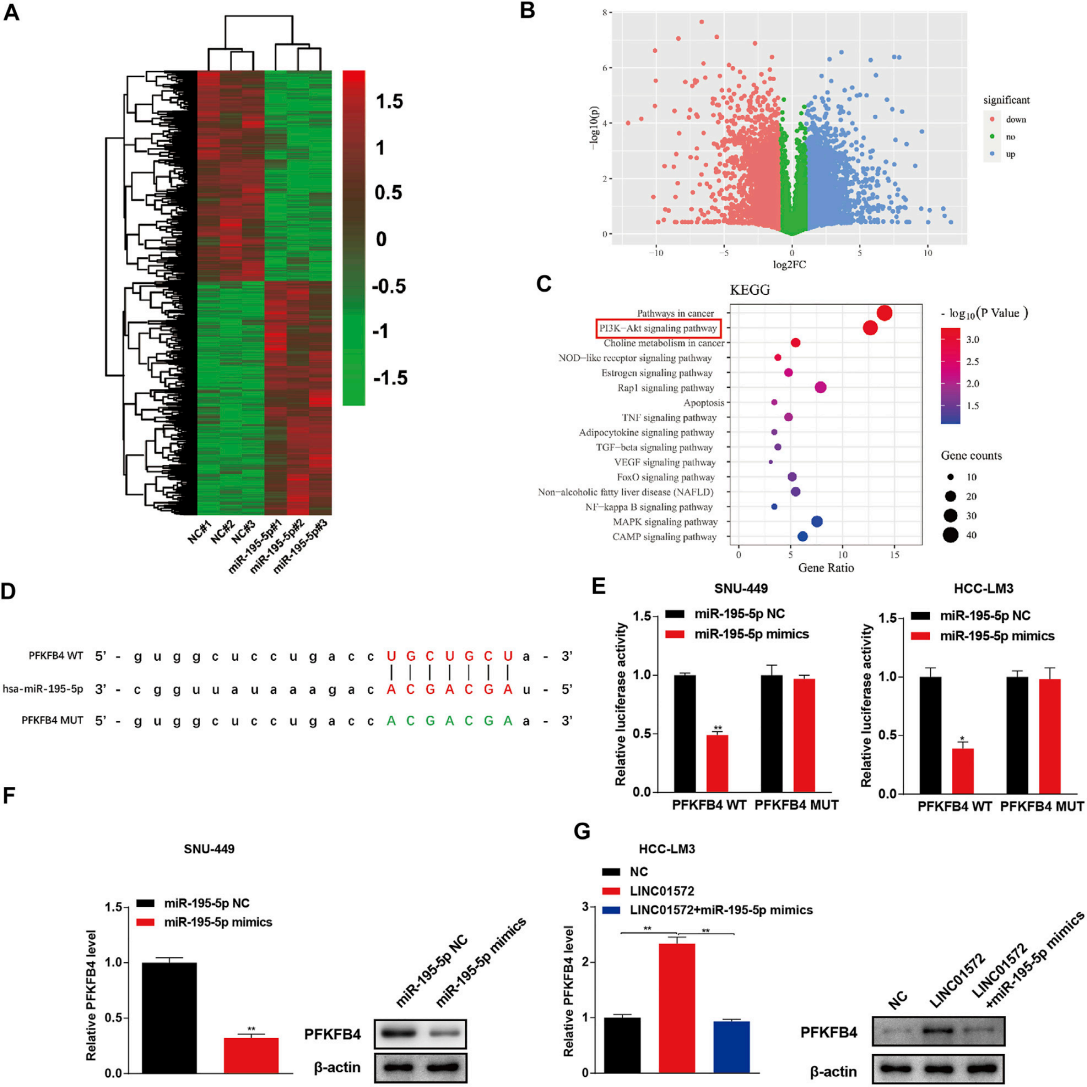
miR-195-5p targets to PFKFB4 in HCC. **(A)** Heatmap of the differentially expressed genes after miR-195-5p overexpressed in SNU-449 cells **(B)** Volcano plot showing the significantly expressed genes. **(C)** KEGG analysis was used to screen the enriched pathways after miR-195-5p overexpressed. **(D)** Binding sites of miR-195-5p and PFKFB4 was predicted by using the ENCORI. **(E)** The target relationship of miR-195-5p and PFKFB4 were verified by dual-luciferase assay. **(F)** Influence of miR-195-5p mimics on the PFKFB4 level in SNU-449 cells. **(G)** Influence of miR-195-5p on LINC01572-induced upregulation of PFKFB4. All data are shown as mean ± standard deviation (*n* = 3). ^*^ *p* < 0.05, ^**^ *p* < 0.01.

In order to determine the molecular mechanism underlying LINC01572/miR-195-5p–triggered HCC malignant behaviors, microarray analysis was performed to identify the potential targets on SNU-449 cells transfected with miR-195-5p mimics and negative control (Figure 6A). As a result, it was found that PFKFB4 was significantly downregulated following miR-195-5p mimics transfection (Figure 6B). The KEGG analysis further showed that PI3K-AKT signaling was potentially involved in the miR-195-5p effect (Figure 6C). The ENCORI prediction algorithm also showed that PFKFB4 was a potential target of miR-195-5p (Figure 6D). The dual-luciferase reporter activity assay showed that miR-195-5p significantly decreased luciferase activity in cells co-transfected with PFKFB4-WT but not PFKFB4-MUT (Figure 6E). Furthermore, miR-195-5p mimics significantly decreased the expression of PFKFB4 and prevented LINC01572-mediated upregulation of PFKFB4 (Figures 6F,G).

**FIGURE 7:**
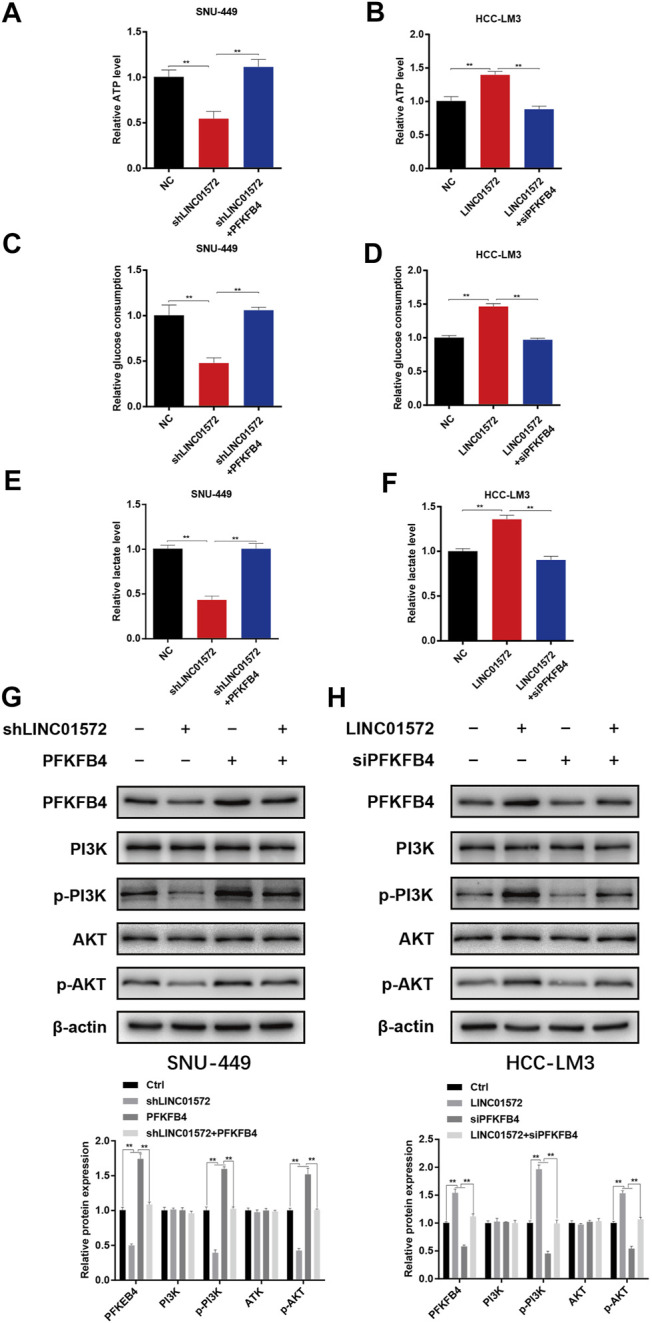
LINC01572 increases glycolysis and activates PI3K/AKT signaling by regulating the PFKFB4 expression. **(A,B)** ATP production, **(C,D)** glucose consumption, and **(E,F)** lactate production following transfection of indicated constructs. **(G,H)** Effect of PFKFB4 overexpression or silencing on PI3K/AKT signaling, following LINC01572 silencing or overexpression, respectively. All data are shown as mean ± standard deviation (*n* = 3). ^**^ *p* < 0.01.

PFKFB4 is a key glycolysis regulator which activates the rate-limiting enzyme phosphofructokinase-1 (PFK-1) in glycolysis *via* its product fructose-2,6-biphosphate and has been proposed recently as a potential oncogene in HCC (Shen et al., 2021). Considering the significance of glycolysis in HCC malignancy (Hu L. et al., 2019; Feng et al., 2020; Gu et al., 2020), we reckoned that LINC01572 might have a glycolysis-promoting potential *via* increase in the PFKFB4 level. In line with this notion, we found that LINC01572 knockdown significantly inhibited the glycolytic process, which was effectively reversed by PFKFB4 overexpression (Figures 7A,C,E), whereas LINC01572 overexpression promoted HCC glycolysis, and PFKFB4 silencing remarkably blunted this effect (Figures 7B,D,F,H).

PI3K-AKT signaling has documented an important HCC-promoting pathway (Liao et al., 2019; Zhao et al., 2021), and a recent study has shown that transcriptional enhancement of the PFKFB4 expression promotes glioma malignancy progression by activating PI3K/AKT signaling (Zhang et al., 2021). We thus analyzed whether PI3K-AKT signaling was modulated by LINC01572 *via* PFKFB4. As expected, our results showed that LINC01572 positively affected the phosphorylation of PI3K/AKT in a PFKFB4-dependent manner (Figures 7G,H). Collectively, these data indicate that LINC01572 enhances PFKFB4 expression to enhance the glycolysis process and activate PI3K-AKT signaling in HCC *via* competitive sponging of miR-195-5p.

### Upregulation of PFKFB4 Inhibits shLINC01572-Mediated Malignant Behaviors in Hepatocellular Carcinoma

**FIGURE 8:**
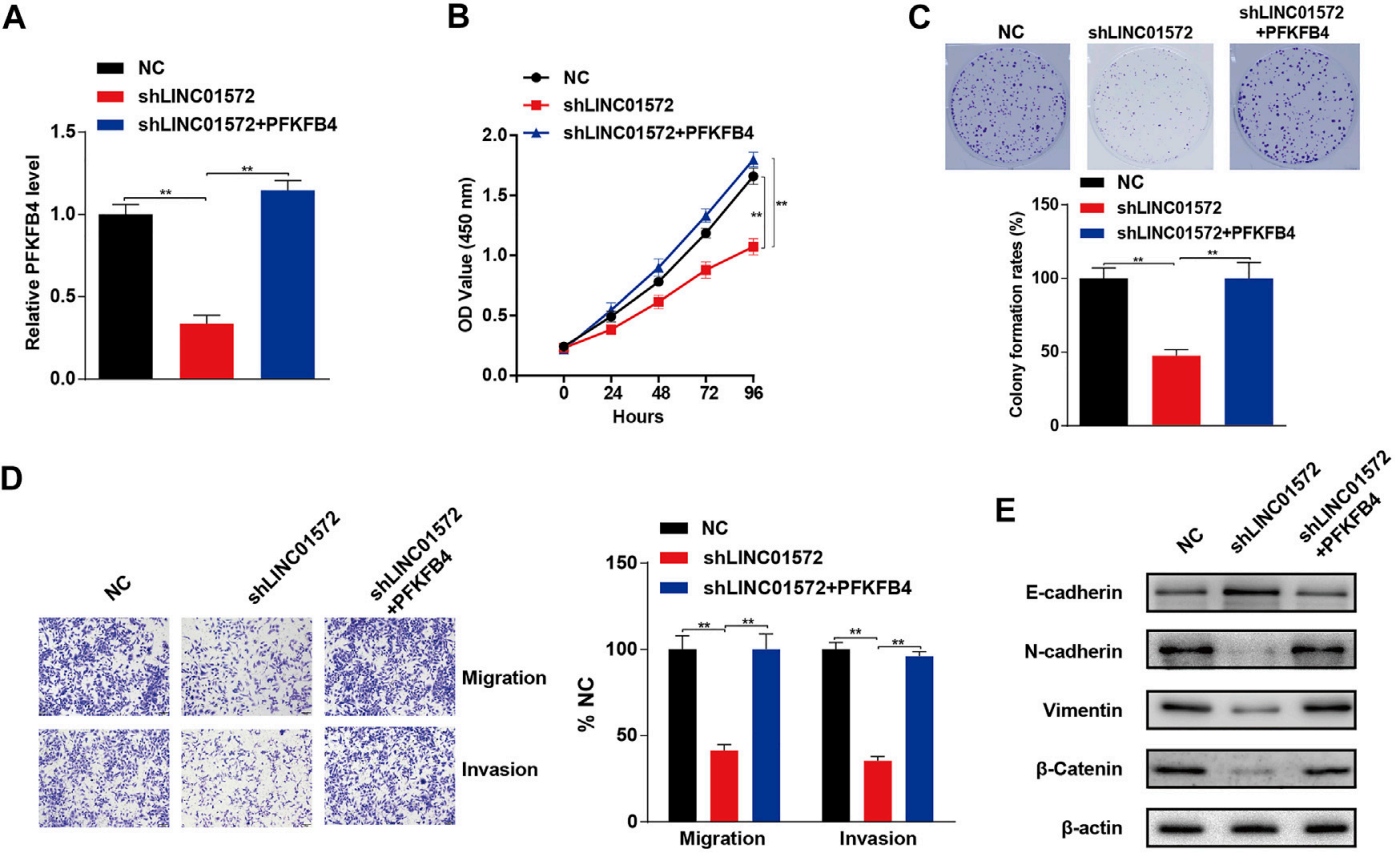
PFKFB4 reverses LINC01572 provoked inhibition of proliferation, invasion, and migration in HCC cells. **(A)** RT-qPCR was used to detect the PFKFB4 level. **(B,C)** cck-8 and colony formation assays assessing the proliferation ability following LINC01572 silencing in the presence or absence of PFKFB4 overexpression. **(D,E)** Effect of LINC01572 silencing on HCC cell migration, invasion, and EMT marker expression in the presence or absence of PFKFB4 overexpression. All data are shown as mean ± standard deviation (*n* = 3). ^**^ *p* < 0.01.

Next, the functional significance of PFKFB4 in LINC01572-mediated HCC malignancy was explored *via* a series of rescue experiments. It was shown that the PFKFB4 level was significantly inhibited by shLINC01572 transfection and restored by PFKFB4 overexpression (Figure 8A). CCK-8 and colony formation demonstrated that shLINC01572-mediated HCC cell proliferation inhibition was significantly blunted by PFKFB4 overexpression (Figures 8B,C). shLINC01572-mediated HCC cell migration and invasion reduction were also restored by PFKFB4 overexpression (Figure 8D). In addition, PFKFB4 overexpression effectively reversed the MET process by shLINC01572 transfection in HCC cells (Figure 8E).

## Discussion

HCC develops primarily due to chronic hepatitis caused by HBV and HCV infection and resultant liver cirrhosis and fibrosis, causing approximately one million deaths each year worldwide (Lombardi et al., 2018). Increasing evidence shows that concurrent T2DM strongly predisposes to the occurrence of HCC (Dyal et al., 2016; Gerbes et al., 2018; Tan et al., 2019). More attention should be paid to HCC patients accompanied by T2DM as past decades have seen a noticeable increase in T2DM and T2DM-related HCC (Shi et al., 2021). This study aimed at identifying potential lncRNA molecules critically involved in T2DM-related HCC. This study demonstrated that LINC01572 was aberrantly upregulated in HCC tissues, especially those from patients complicated by T2DM. A mechanistic study revealed that LINC01572 promoted HCC malignancy *via* sponging miR-195-5p to enhance PFKFB4-mediated glycolysis and PI3K/AKT signaling activation.

LncRNA has been proven to be critically associated with the occurrence, progression, and metastasis of cancers (Hu Y.-p. et al., 2019; Huang et al., 2021). LINC01572 is a newly recognized lncRNA molecule, and functionally, only one study reported that it mediated cisplatin resistance in gastric cancer cells (Song et al., 2020). However, its role in HCC is still unknown. In this study, we profiled lncRNAs in HCC tissues and corresponding adjacent tissues from HCC patients with T2DM by RNA sequencing. Our data showed that LINC01572 was aberrantly upregulated in HCC tissues as compared with the control, especially in those with concurrent T2DM. The high level of LINC01572 was correlated with the advanced T stage, increased blood HbA1c level, and shortened survival time. The overexpression of LINC01572 significantly promoted HCC cell proliferation, migration, invasion, and epithelial-to-mesenchymal transition (EMT), while the knockdown of LINC01572 had the opposite effects on HCC cells. Furthermore, silencing LINC01572 significantly restricted the growth and metastasis of HCC *in vivo*. Collectively, these results suggest that LINC01572 is carcinogenic and plays a role in the progression of HCC, particularly T2DM-related HCC.

We subsequently performed mechanistic experiments to further understand how LINC01572 modulates HCC progression. LncRNAs might adopt different mechanisms to regulate gene expression, depending on their subcellular location. Generally, lncRNAs located in the nucleus interact with RNA-binding proteins to control gene expression at transcriptional level (Zhang et al., 2020), whereas cytoplasmic lncRNAs might interact with miRNA as a competitive endogenous RNA (ceRNA), controlling gene expression at the post-transcriptional level (Han et al., 2020). A previous study showed that LINC01572 enhanced HCC cell proliferation and migration by inducing the transcription of downstream target genes as an eRNA(Lee et al., 2021). In this study, we found that LINC01572 was not only distributed in the nucleus but also in the cytoplasm of HCC cells, implying that ceRNA mechanism might be alternatively involved in LINC01572-mediated HCC malignancy.

Using ENCORI, we predicted that LINC01572 had a possible interaction with miRNA-195-5p. This predication was further confirmed by a series of data in our study, including those from the double-luciferase assay, RIP assay, RNA pull-down experiments, and modulating relationship between LINC01572 and miRNA-195-5p as revealed by RT-qPCR. These results indicate that LINC01572 might exert its oncogenic role in HCC *via* target binding to miRNA-195-5p, which seems to be plausible as miRNA-195-5p has documented to be a tumor suppressor in HCC (Xu et al., 2015).

Subsequently, using high-throughput screening and various confirmatory experiments, we presented evidence showing that 6-phosphofructo-2-kinase/fructose-2, 6-bisphosphatase 4 (PFKFB4) is a critical target molecule to mediate LINC01572/miR-195-5p effects in HCC. In actual fact, the carcinogenic role of PFKFB4 has been documented in a variety of malignant tumors, including in HCC (Cai et al., 2021; Shen et al., 2021; Wang et al., 2021). Considering that in cancers, PFKFB4 is critically involved in enhanced aerobic glycolysis, a phenomenon termed the Warburg effect (Chen et al., 2021; Zhou et al., 2021), and we reckoned LINC01572 might exert its oncogenic effect *via* PFKFB4-enhanced glycolysis. This postulation was corroborated in our study as the influence of LINC01572 on both glycolysis and HCC malignant behaviors was dependent on the PFKFB4 level. In addition to mediating the Warburg effect, our KEGG enrichment analysis revealed that the PI3K/AKT pathway significantly affected the signaling transduction process by miR-195-5p. In line with this, our data showed that PI3K/AKT signaling was under control of LINC01572. More importantly, this process was PFKFB4-dependent, indicating that PFKFB4-mediated PI3K/AKT signaling activation represents another paralleled mechanism to enhanced glycolysis mediating the LINC01572 effect in HCC. Consistently, it has been documented that PFKFB4 exerts its oncogenic effect on glioma *via* increased activation of PI3K/AKT signaling (Zhang et al., 2021). Collectively, our results support that PFKFB4 relays the LINC01572 effect in HCC *via* enhanced glycolysis and paralleled hyperactivated PI3K/AKT signaling.

Taken together, we herein provide evidence demonstrating that lncRNA LINC01572 plays a pivotal role in promoting HCC cell malignancy *via* a ceRNA mechanism involving miR-195-5p and the glycolytic rate–limiting enzyme PFKFB4. This finding suggests a potential target for diagnosis, treatment, and prognosis of HCC, especially those complicated by T2DM.

## Data Availability

The original contributions presented in the study are publicly available. This data can be found here: https://doi.org/10.6084/m9.figshare.16968199.v1, and https://doi.org/10.6084/m9.figshare.16968247.v1.

## References

[B1] Cai Y.-C. Yang H. Shan H.-B. Su H.-F. Jiang W.-Q. Shi Y.-X. (2021). PFKFB4 Overexpression Facilitates Proliferation by Promoting the G1/S Transition and Is Associated with a Poor Prognosis in Triple-Negative Breast Cancer. Dis. Markers 2021, 8824589. 10.1155/2021/8824589 34211613 PMC8211511

[B2] Chen J. Huang X. Wang W. Xie H. Li J. Hu Z. (2018). LncRNA CDKN2BAS Predicts Poor Prognosis in Patients with Hepatocellular Carcinoma and Promotes Metastasis via the miR-153-5p/ARHGAP18 Signaling axis. Aging 10 (11), 3371–3381. 10.18632/aging.101645 30510148 PMC6286843

[B3] Chen W.-J. Tang R.-X. He R.-Q. Li D.-Y. Liang L. Zeng J.-H. (2017). Clinical Roles of the Aberrantly Expressed lncRNAs in Lung Squamous Cell Carcinoma: a Study Based on RNA-Sequencing and Microarray Data Mining. Oncotarget 8 (37), 61282–61304. 10.18632/oncotarget.18058 28977863 PMC5617423

[B4] Chen Y. Song S. Zhang L. Zhang Y. (2021). Circular RNA Hsa_circ_0091579 Facilitates the Warburg Effect and Malignancy of Hepatocellular Carcinoma Cells via the miR-624/H3F3B axis. Clin. Transl Oncol. 23, 2280–2292. 10.1007/s12094-021-02627-4 33934291

[B5] Dyal H. K. Aguilar M. Bartos G. Holt E. W. Bhuket T. Liu B. (2016). Diabetes Mellitus Increases Risk of Hepatocellular Carcinoma in Chronic Hepatitis C Virus Patients: A Systematic Review. Dig. Dis. Sci. 61 (2), 636–645. 10.1007/s10620-015-3983-3 26703125

[B6] Feng J. Dai W. Mao Y. Wu L. Li J. Chen K. (2020). Simvastatin Re-sensitizes Hepatocellular Carcinoma Cells to Sorafenib by Inhibiting HIF-1α/ppar-Γ/pkm2-Mediated Glycolysis. J. Exp. Clin. Cancer Res. 39 (1), 24. 10.1186/s13046-020-1528-x 32000827 PMC6993409

[B7] Ge Y. Gu P. Wang W. Cao L. Zhang L. Li J. (2021). Benzo[a]pyrene Stimulates miR-650 Expression to Promote the Pathogenesis of Fatty Liver Disease and Hepatocellular Carcinoma via SOCS3/JAK/STAT3 Cascades. J. Mol. Cel. Biol. 13, 556. 10.1093/jmcb/mjab052 PMC8697348 34450627

[B8] Gerbes A. Zoulim F. Tilg H. (2018). Correction: Gut Roundtable Meeting Paper: Selected Recent Advances in Hepatocellular Carcinoma. Gut 67 (3), 594. 10.1136/gutjnl-2017-315068corr1 29439114

[B9] Gu Y. Ji F. Liu N. Zhao Y. Wei X. Hu S. (2020). Loss of miR-192-5p Initiates a Hyperglycolysis and Stemness Positive Feedback in Hepatocellular Carcinoma. J. Exp. Clin. Cancer Res. 39 (1), 268. 10.1186/s13046-020-01785-7 33256802 PMC7708108

[B10] Han T.-S. Hur K. Cho H.-S. Ban H. S. (2020). Epigenetic Associations between lncRNA/circRNA and miRNA in Hepatocellular Carcinoma. Cancers 12 (9), 2622. 10.3390/cancers12092622 PMC7565033 32937886

[B11] Hu L. Zeng Z. Xia Q. Liu Z. Feng X. Chen J. (2019a). Metformin Attenuates Hepatoma Cell Proliferation by Decreasing Glycolytic Flux through the HIF-1α/PFKFB3/PFK1 Pathway. Life Sci. 239, 116966. 10.1016/j.lfs.2019.116966 31626790

[B12] Hu Y.-p. Jin Y.-p. Wu X.-s. Yang Y. Li Y.-s. Li H.-f. (2019b). LncRNA-HGBC Stabilized by HuR Promotes Gallbladder Cancer Progression by Regulating miR-502-3p/SET/AKT axis. Mol. Cancer 18 (1), 167. 10.1186/s12943-019-1097-9 31752906 PMC6868746

[B13] Hu X. Zhu H. Shen Y. Zhang X. He X. Xu X. (2021). The Role of Non-coding RNAs in the Sorafenib Resistance of Hepatocellular Carcinoma. Front. Oncol. 11, 696705. 10.3389/fonc.2021.696705 34367979 PMC8340683

[B14] Huang X. Pan L. Zuo Z. Li M. Zeng L. Li R. (2021). LINC00842 Inactivates Transcription Co-regulator PGC-1α to Promote Pancreatic Cancer Malignancy through Metabolic Remodelling. Nat. Commun. 12 (1), 3830. 10.1038/s41467-021-23904-4 34158490 PMC8219694

[B15] Jin Z.-L. Liu W. (2021). Progress in Treatment of Type 2 Diabetes by Bariatric Surgery. World J. Diabetes 12 (8), 1187–1199. 10.4239/wjd.v12.i8.1187 34512886 PMC8394224

[B16] Lee Y.-E. Lee J. Lee Y. S. Jang J. J. Woo H. Choi H. I. (2021). Identification and Functional Characterization of Two Noncoding RNAs Transcribed from Putative Active Enhancers in Hepatocellular Carcinoma. Mol.Cells 44 (9), 658–669. 10.14348/molcells.2021.0173 34588321 PMC8490203

[B17] Liao J. Jin H. Li S. Xu L. Peng Z. Wei G. (2019). Apatinib Potentiates Irradiation Effect via Suppressing PI3K/AKT Signaling Pathway in Hepatocellular Carcinoma. J. Exp. Clin. Cancer Res. 38 (1), 454. 10.1186/s13046-019-1419-1 31694662 PMC6836669

[B18] Lombardi A. Grimaldi A. Zappavigna S. Misso G. Caraglia M. (2017). Hepatocarcinoma: Genetic and Epigenetic Features. Minerva Gastroenterol. 64 (1), 14–27. 10.23736/s1121-421x.17.02408-4 28398025

[B19] Mohan C. D. Rangappa S. Nayak S. C. Sethi G. Rangappa K. S. (2021). Paradoxical Functions of Long Noncoding RNAs in Modulating STAT3 Signaling Pathway in Hepatocellular Carcinoma. Biochim. Biophys. Acta (Bba) - Rev. Cancer 1876 (1), 188574. 10.1016/j.bbcan.2021.188574 34062154

[B20] Shen C. Ding L. Mo H. Liu R. Xu Q. Tu K. (2021). Long Noncoding RNA FIRRE Contributes to the Proliferation and Glycolysis of Hepatocellular Carcinoma Cells by Enhancing PFKFB4 Expression. J. Cancer 12 (13), 4099–4108. 10.7150/jca.58097 34093813 PMC8176253

[B21] Shi T. Kobara H. Oura K. Masaki T. (2021). Mechanisms Underlying Hepatocellular Carcinoma Progression in Patients with Type 2 Diabetes. J. Hepatocell Carcinoma 8, 45–55. 10.2147/jhc.S274933 33604315 PMC7886236

[B22] Singh M. K. Das B. K. Choudhary S. Gupta D. Patil U. K. (2018). Diabetes and Hepatocellular Carcinoma: A Pathophysiological Link and Pharmacological Management. Biomed. Pharmacother. 106, 991–1002. 10.1016/j.biopha.2018.06.095 30119271

[B23] Song Z. Jia N. Li W. Zhang X.-Y. (2020). LINC01572 Regulates Cisplatin Resistance in Gastric Cancer Cells by Mediating miR-497-5p. Onco Targets Ther. 13, 10877–10887. 10.2147/ott.S267915 33149605 PMC7602899

[B24] Sun C. Huang S. Hou Y. Li Z. Xia D. Zhang L. (2020). Long Noncoding RNA AC092171.4 Promotes Hepatocellular Carcinoma Progression by Sponging microRNA-1271 and Upregulating GRB2. Aging 12 (14), 14141–14156. 10.18632/aging.103419 32692718 PMC7425487

[B25] Tan Y. Wei S. Zhang W. Yang J. Yang J. Yan L. (2019). Type 2 Diabetes Mellitus Increases the Risk of Hepatocellular Carcinoma in Subjects with Chronic Hepatitis B Virus Infection: a Meta-Analysis and Systematic Review. Cancer Manag. Res. 11, 705–713. 10.2147/cmar.S188238 30679924 PMC6338123

[B26] Teng F. Zhang J.-X. Chang Q.-M. Wu X.-B. Tang W.-G. Wang J.-F. (2020). Correction to: LncRNA MYLK-AS1 Facilitates Tumor Progression and Angiogenesis by Targeting miR-424-5p/E2F7 axis and Activating VEGFR-2 Signaling Pathway in Hepatocellular Carcinoma. J. Exp. Clin. Cancer Res. 39 (1), 277. 10.1186/s13046-020-01780-y 33298087 PMC7724876

[B27] Wang F. Wu X. Li Y. Cao X. Zhang C. Gao Y. (2021). PFKFB4 as a Promising Biomarker to Predict a Poor Prognosis in Patients with Gastric Cancer. Oncol. Lett. 21 (4), 296. 10.3892/ol.2021.12557 33732372 PMC7905623

[B28] Wild S. H. (2011). Diabetes, Treatments for Diabetes and Their Effect on Cancer Incidence and Mortality: Attempts to Disentangle the Web of Associations. Diabetologia 54 (7), 1589–1592. 10.1007/s00125-011-2169-6 21541783

[B29] Xu H. Hu Y.-W. Zhao J.-Y. Hu X.-M. Li S.-F. Wang Y.-C. (2015). MicroRNA-195-5p Acts as an Anti-oncogene by Targeting PHF19 in Hepatocellular Carcinoma. Oncol. Rep. 34 (1), 175–182. 10.3892/or.2015.3957 25955388

[B30] Zhang L. Liu Z. Dong Y. Kong L. (2021). E2F2 Drives Glioma Progression via PI3K/AKT in a PFKFB4-dependent Manner. Life Sci. 276, 119412. 10.1016/j.lfs.2021.119412 33774025

[B31] Zhang X.-Z. Liu H. Chen S.-R. (2020). Mechanisms of Long Non-coding RNAs in Cancers and Their Dynamic Regulations. Cancers 12 (5), 1245. 10.3390/cancers12051245 PMC7281179 32429086

[B32] Zhao L. Hu K. Cao J. Wang P. Li J. Zeng K. (2019). lncRNA Miat Functions as a ceRNA to Upregulate Sirt1 by Sponging miR-22-3p in HCC Cellular Senescence. Aging 11 (17), 7098–7122. 10.18632/aging.102240 31503007 PMC6756895

[B33] Zhao T. Guo B.-J. Xiao C.-L. Chen J.-J. Lü C. Fang F.-F. (2021). Aerobic Exercise Suppresses Hepatocellular Carcinoma by Downregulating Dynamin-Related Protein 1 through PI3K/AKT Pathway. J. Integr. Med. 19 (5), 418–427. 10.1016/j.joim.2021.08.003 34454893

[B34] Zhou Y. Lin F. Wan T. Chen A. Wang H. Jiang B. (2021). ZEB1 Enhances Warburg Effect to Facilitate Tumorigenesis and Metastasis of HCC by Transcriptionally Activating PFKM. Theranostics 11 (12), 5926–5938. 10.7150/thno.56490 33897890 PMC8058737

