# Supplementary figures and images for: Long Non-Coding RNA LINC01572 Promotes Hepatocellular Carcinoma Progression via Sponging miR-195-5p to Enhance PFKFB4-Mediated Glycolysis and PI3K/AKT Activation
Source: Front Cell Dev Biol. 2021 Dec 14;9:783088. doi: 10.3389/fcell.2021.783088 (PMC8712893; doi:10.3389/fcell.2021.783088)

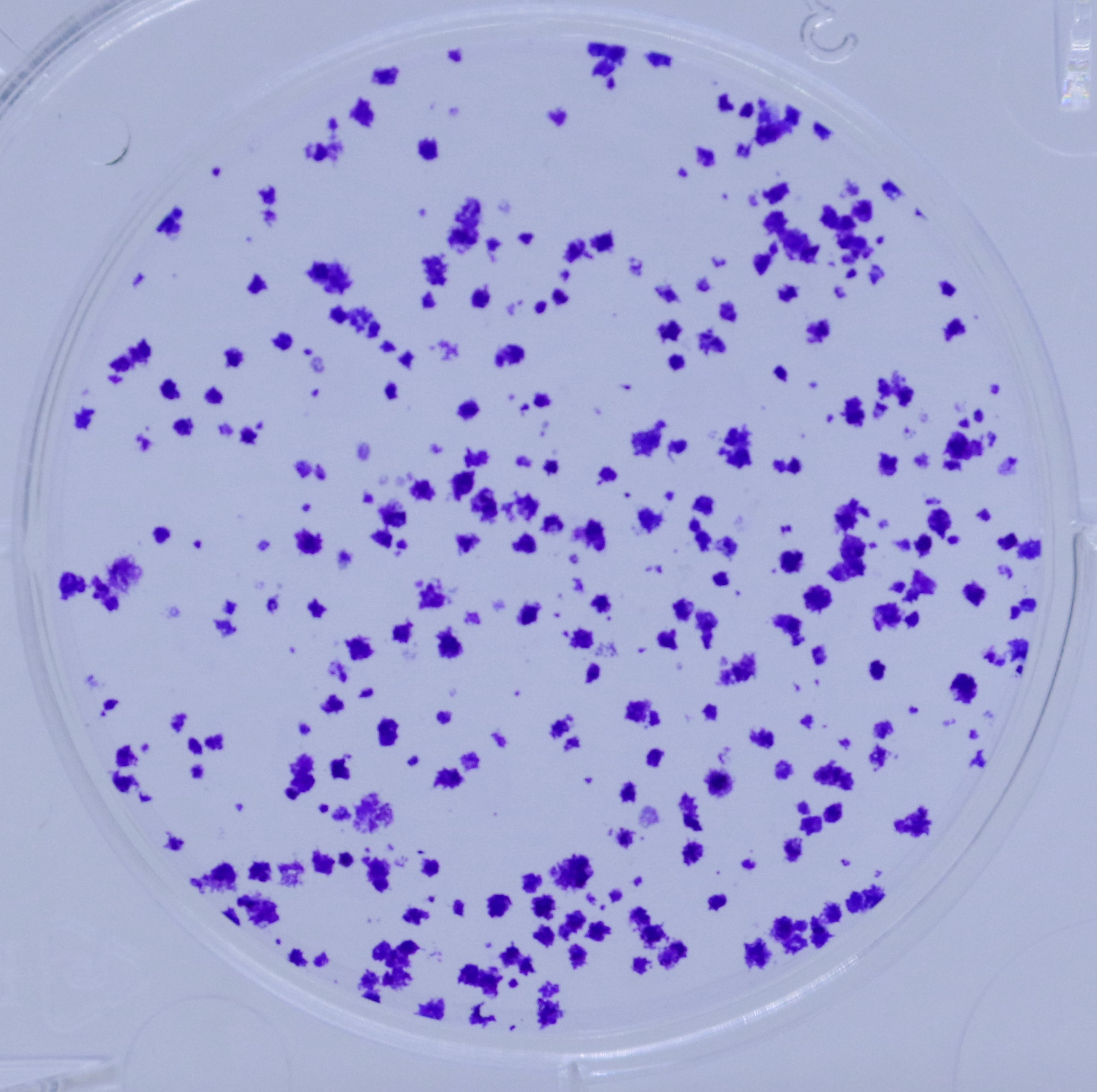

Supplement: Supplementary file 1 [file DataSheet1.zip › Figure 2 Excel/D/Huh7/shCtrl.JPG]

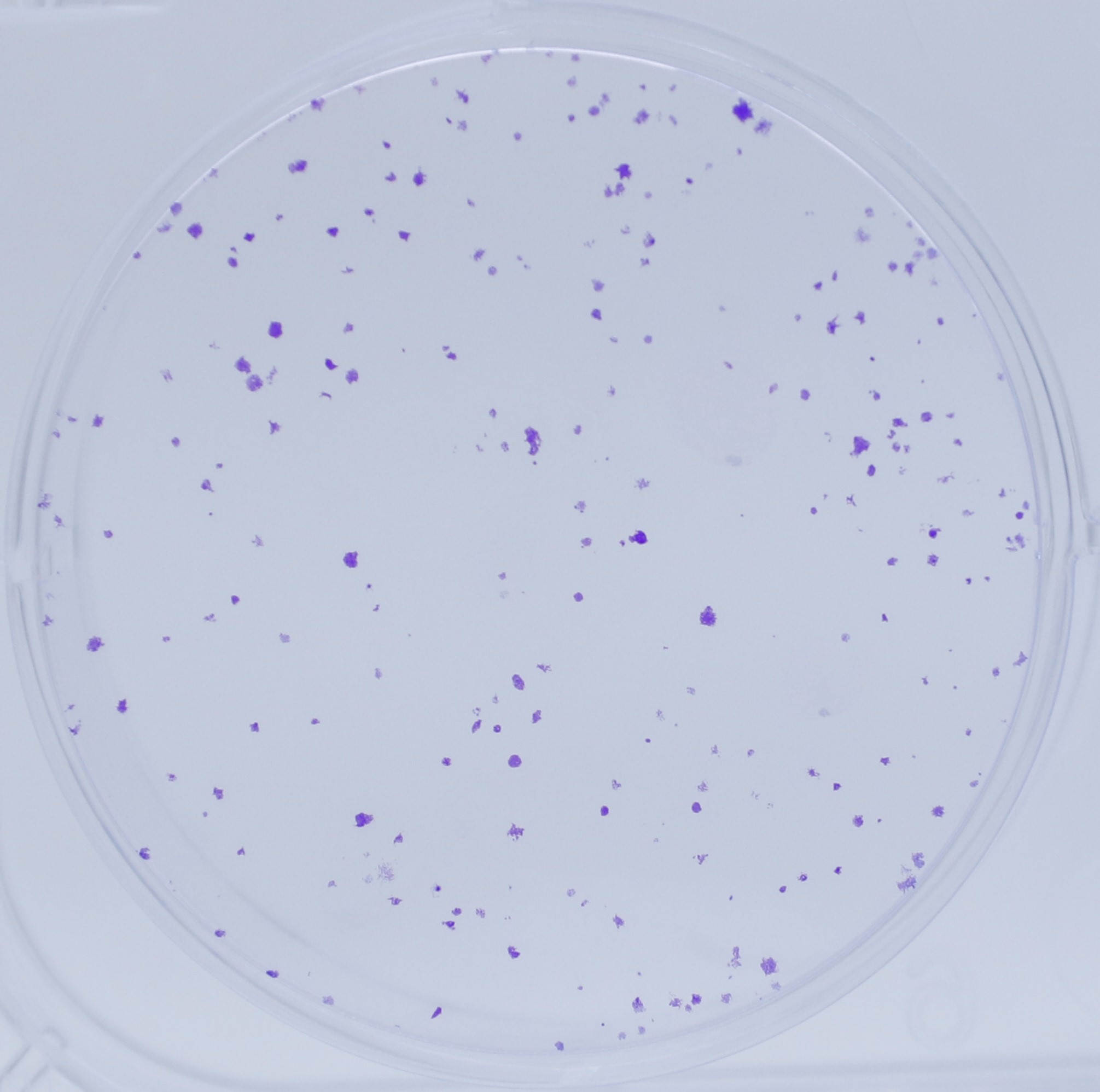

Supplement: Supplementary file 1 [file DataSheet1.zip › Figure 2 Excel/D/Huh7/shLINC01572#1.JPG]

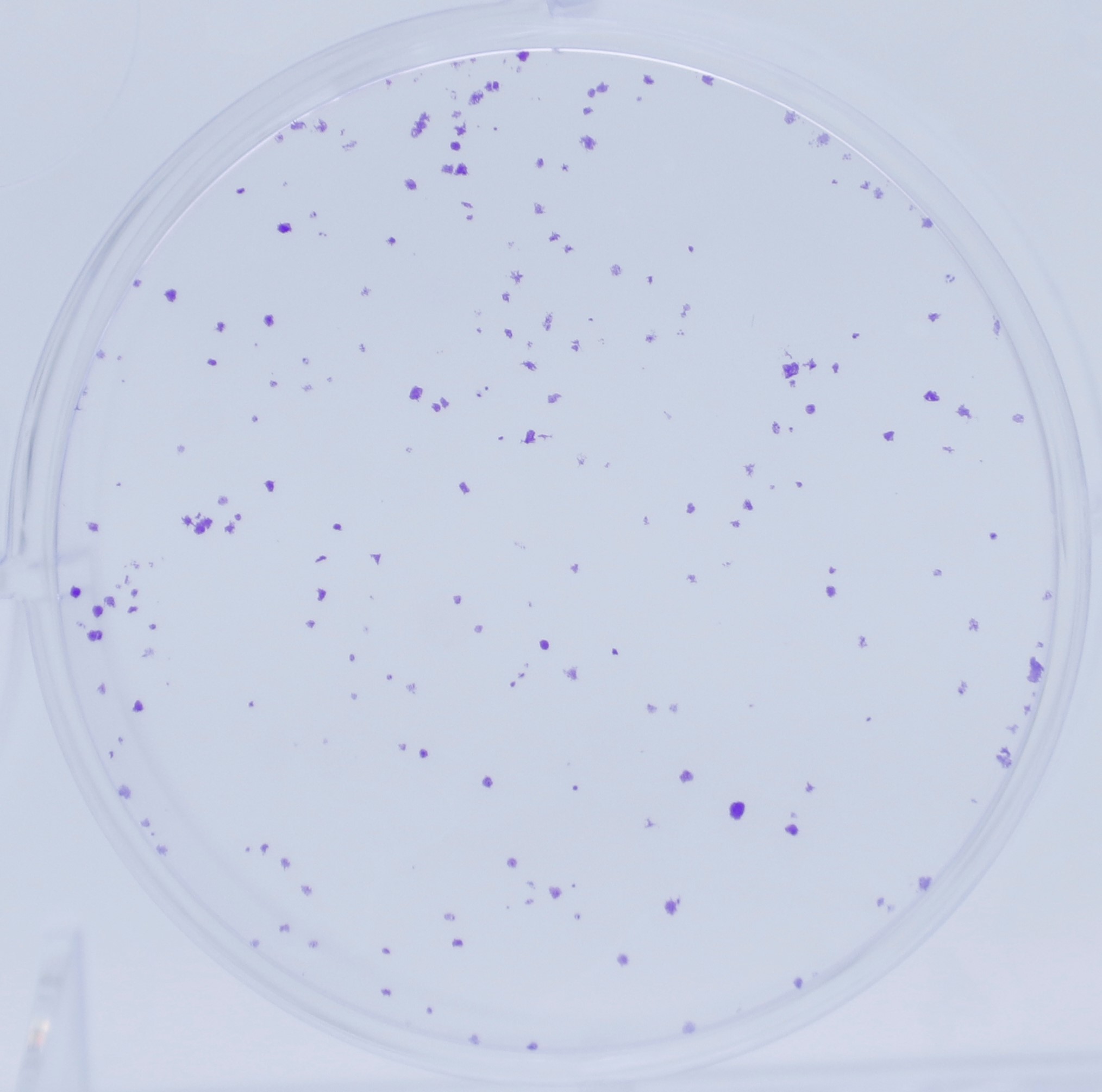

Supplement: Supplementary file 1 [file DataSheet1.zip › Figure 2 Excel/D/Huh7/shLINC01572#2.JPG]

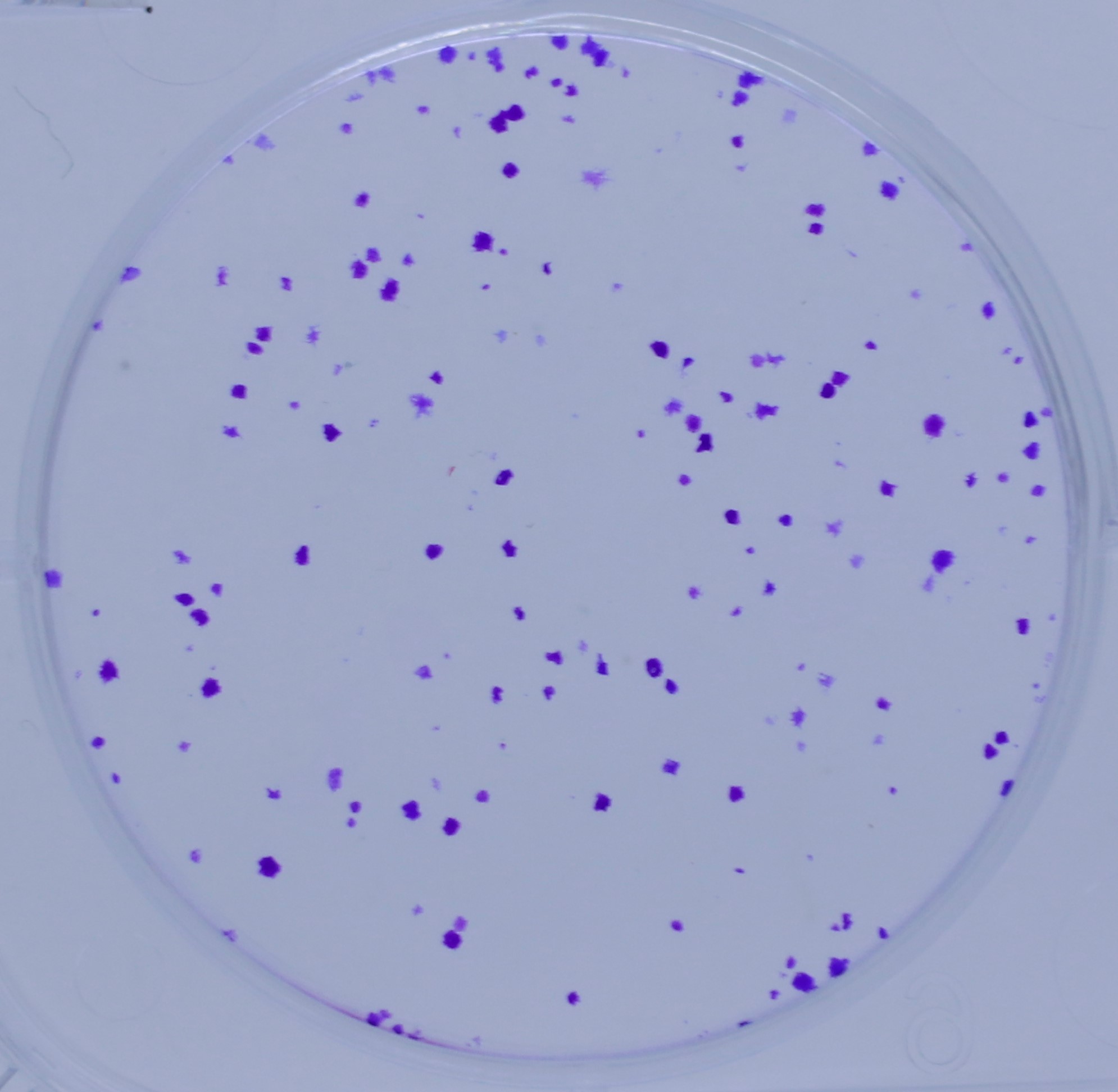

Supplement: Supplementary file 1 [file DataSheet1.zip › Figure 2 Excel/D/SNU-449/shCtrl.JPG]

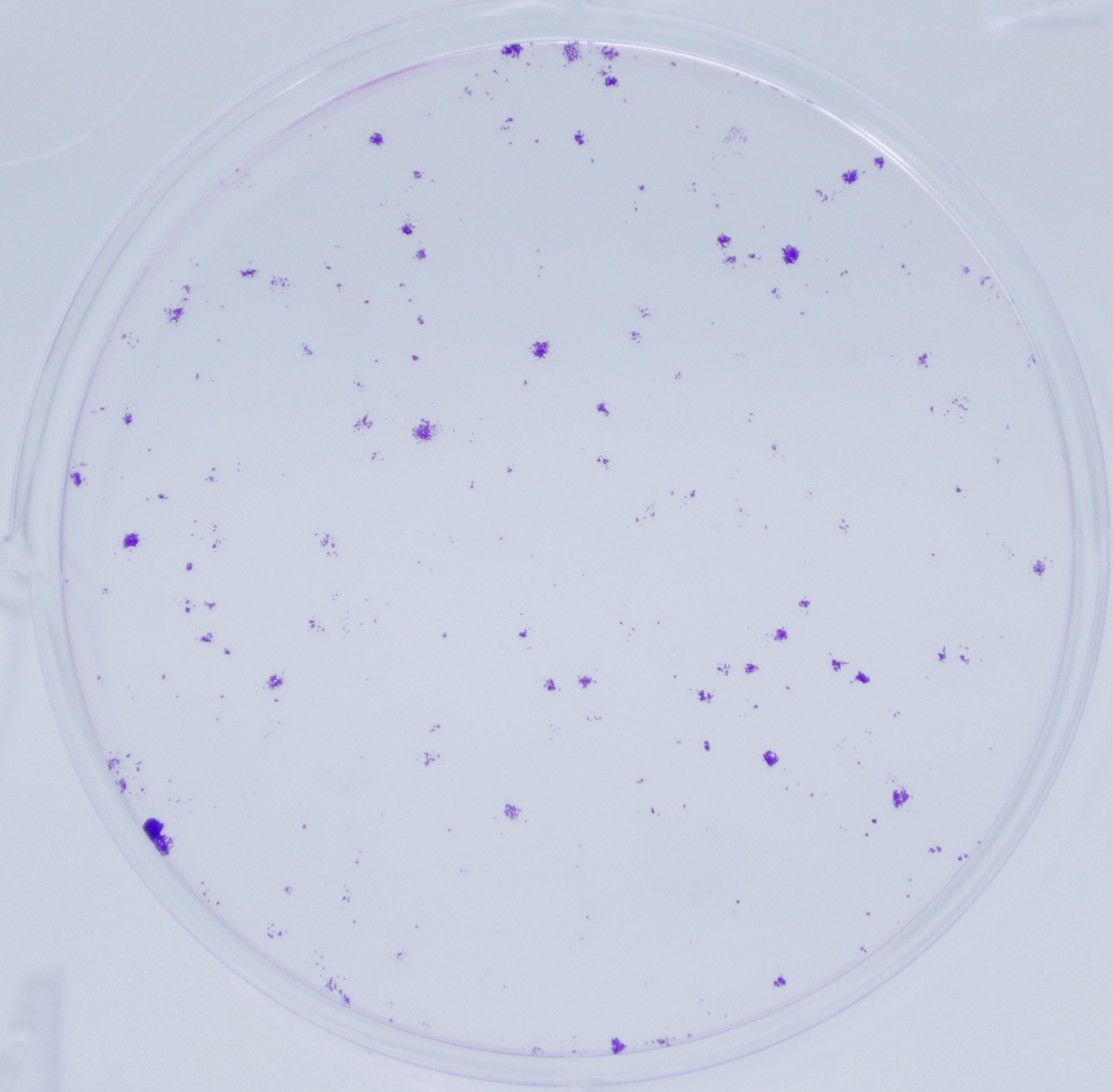

Supplement: Supplementary file 1 [file DataSheet1.zip › Figure 2 Excel/D/SNU-449/shLINC01572#1.JPG]

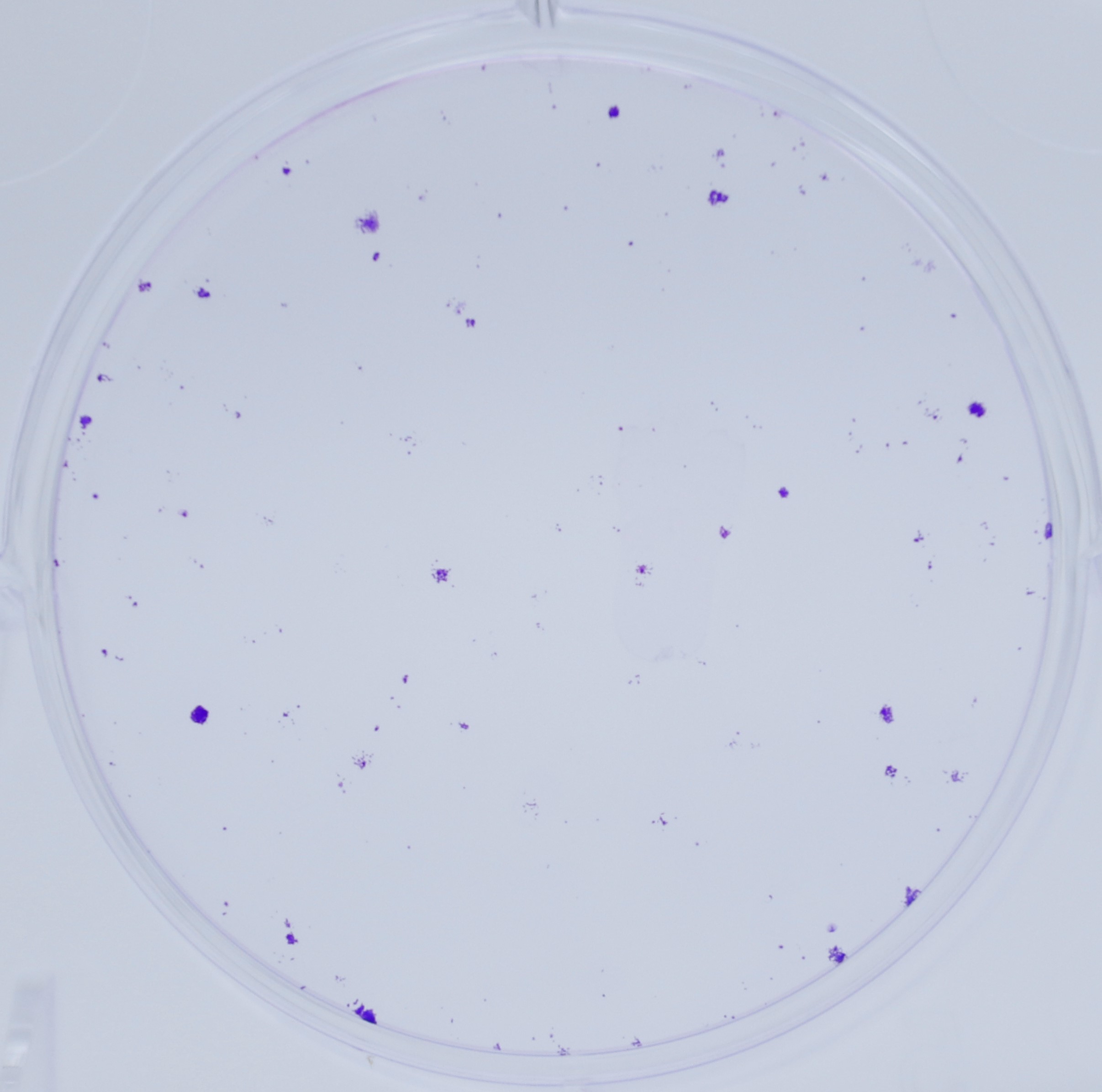

Supplement: Supplementary file 1 [file DataSheet1.zip › Figure 2 Excel/D/SNU-449/shLINC01572#2.JPG]

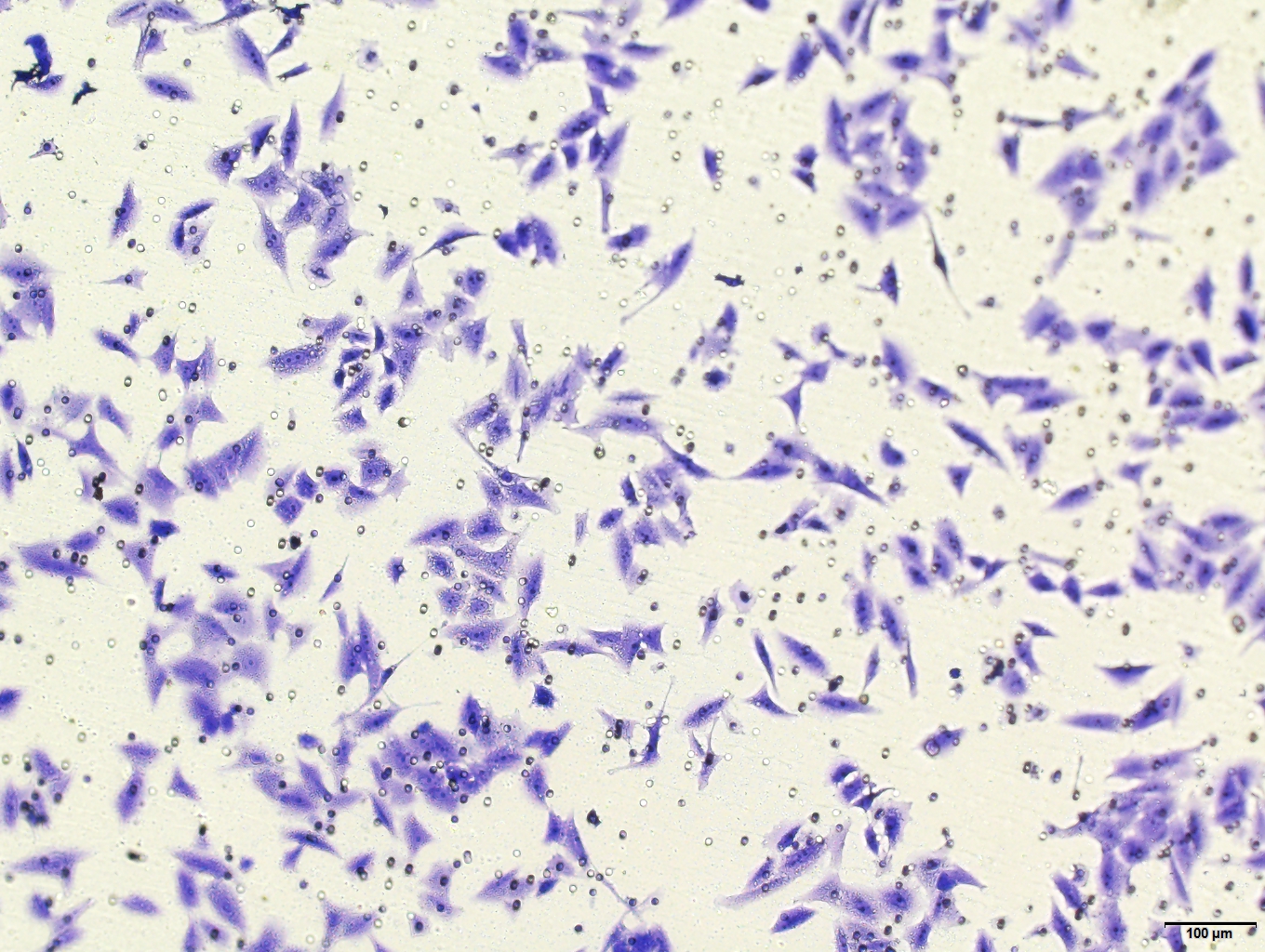

Supplement: Supplementary file 1 [file DataSheet1.zip › Figure 2 Excel/E/Huh7/Invasion/shCtrl.jpg]

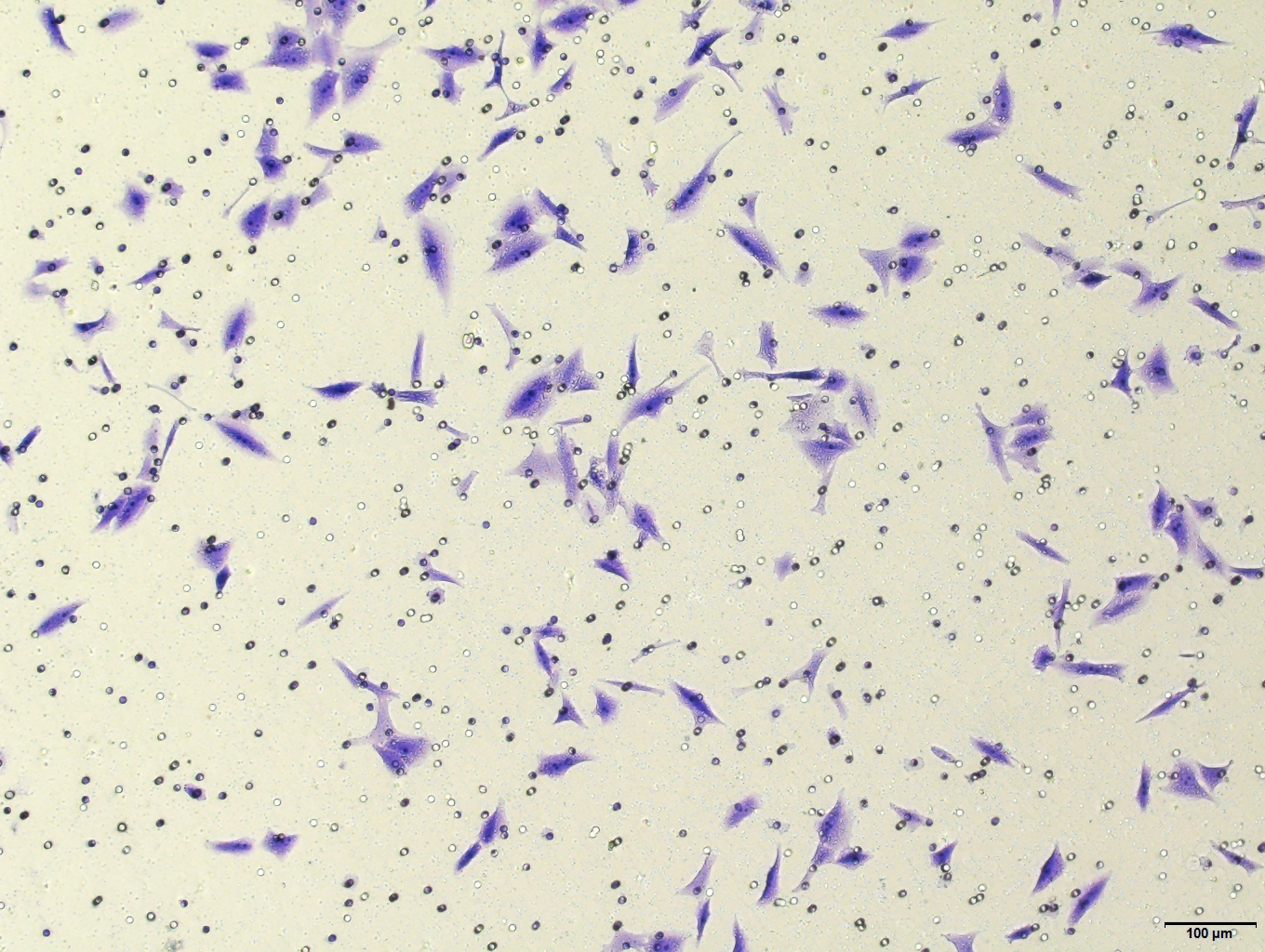

Supplement: Supplementary file 1 [file DataSheet1.zip › Figure 2 Excel/E/Huh7/Invasion/shLINC01572#1.jpg]

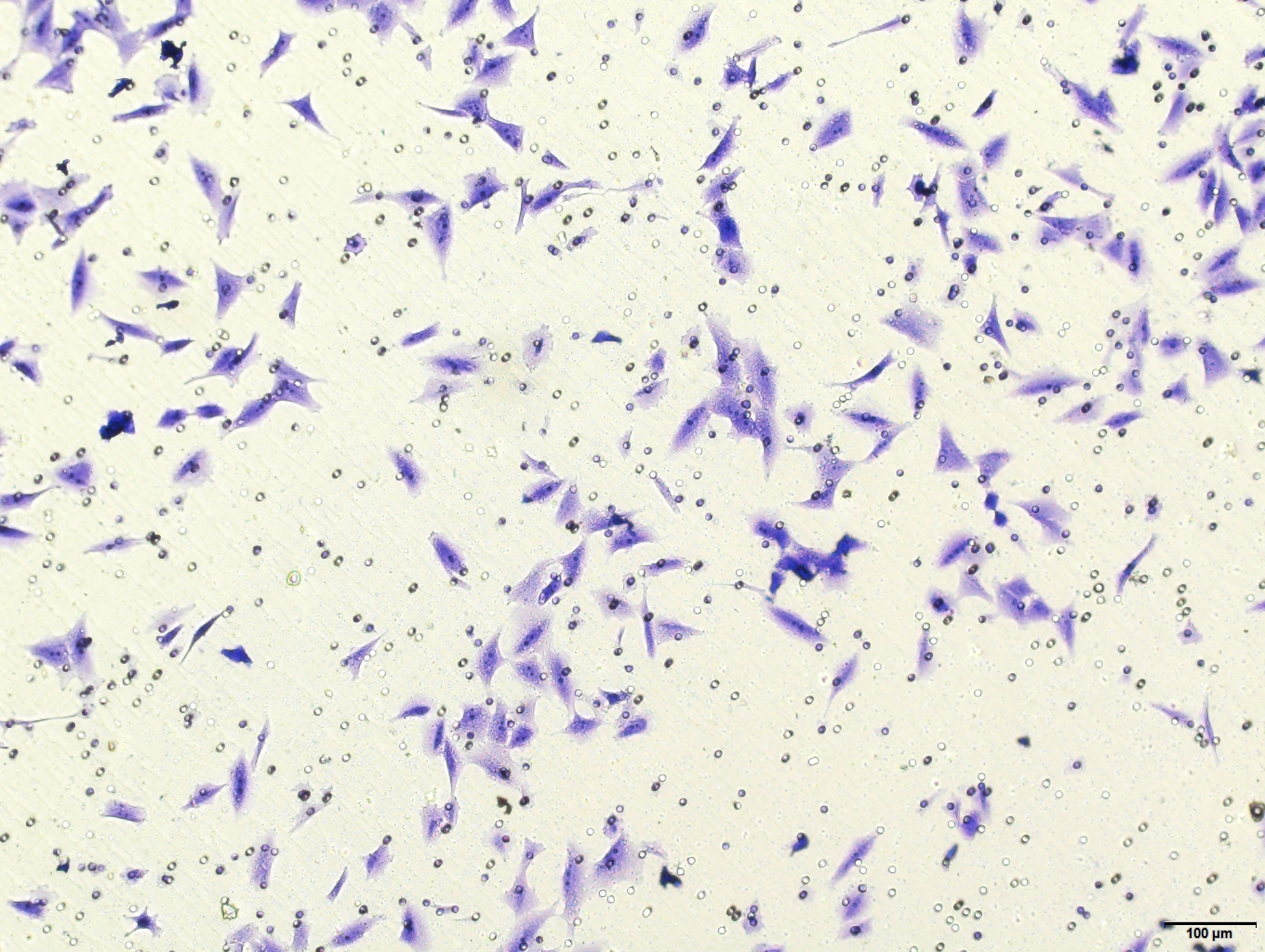

Supplement: Supplementary file 1 [file DataSheet1.zip › Figure 2 Excel/E/Huh7/Invasion/shLINC01572#2.jpg]

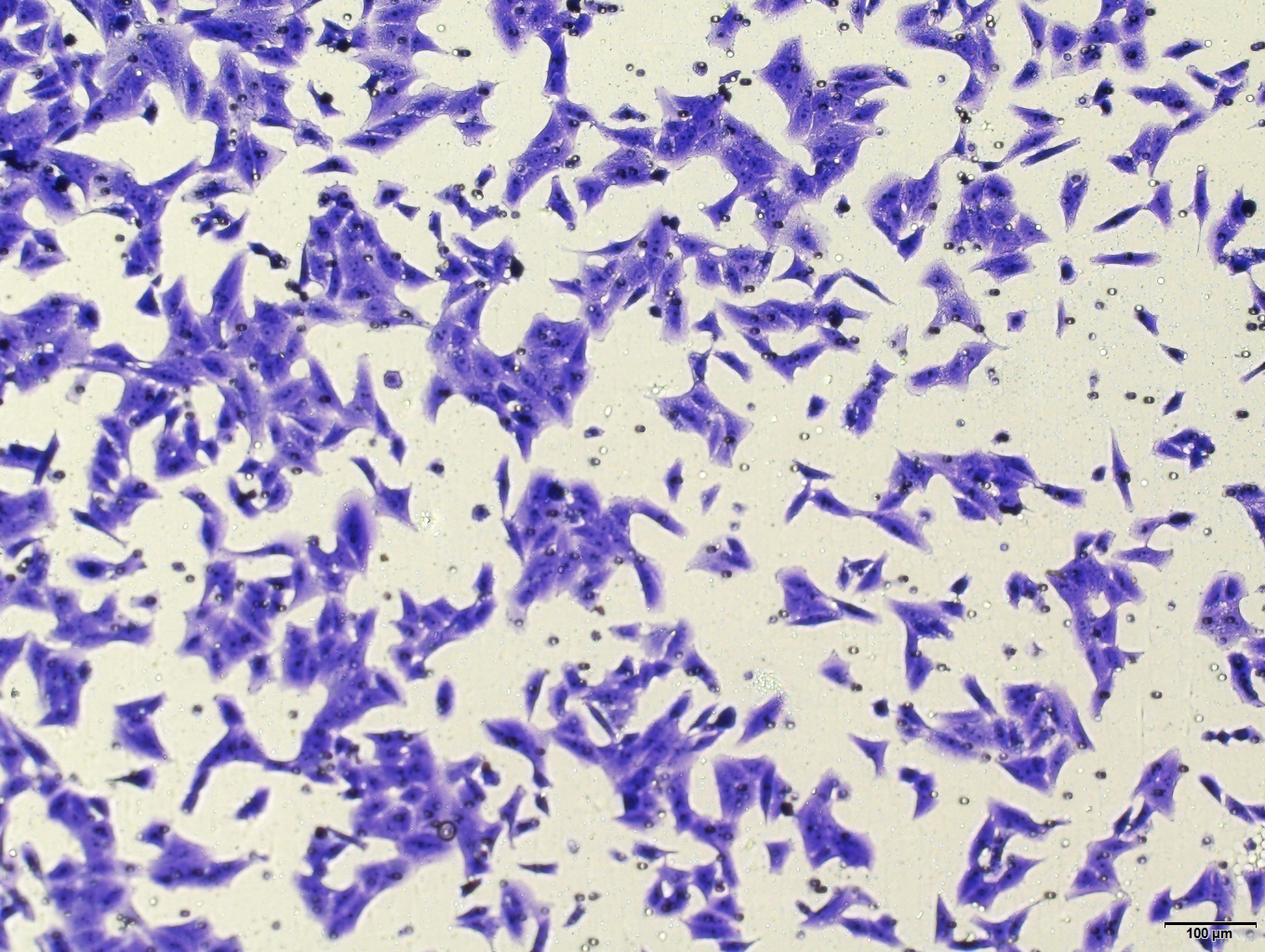

Supplement: Supplementary file 1 [file DataSheet1.zip › Figure 2 Excel/E/Huh7/Migration/shCtrl.jpg]

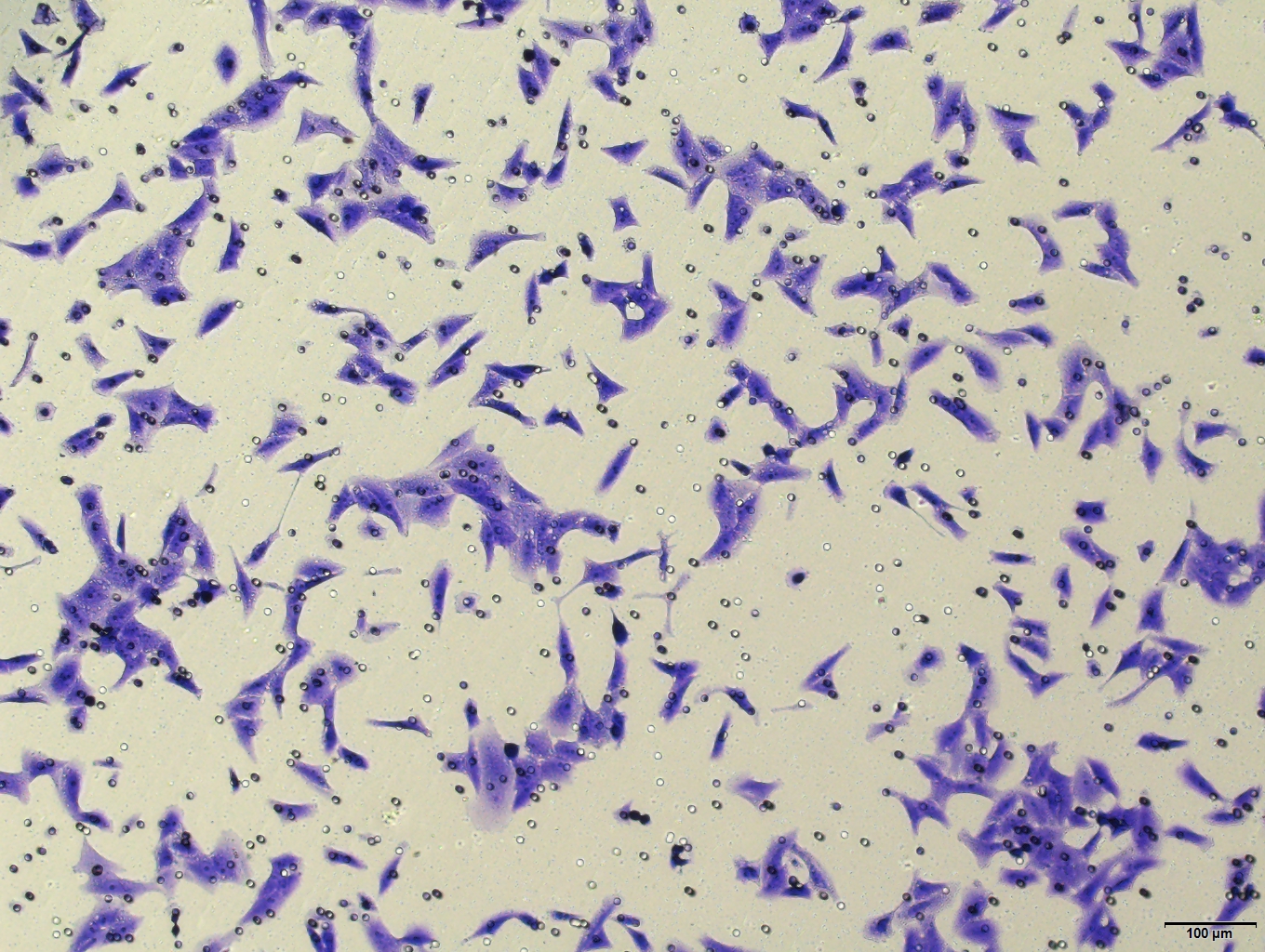

Supplement: Supplementary file 1 [file DataSheet1.zip › Figure 2 Excel/E/Huh7/Migration/shLINC01572#1.jpg]

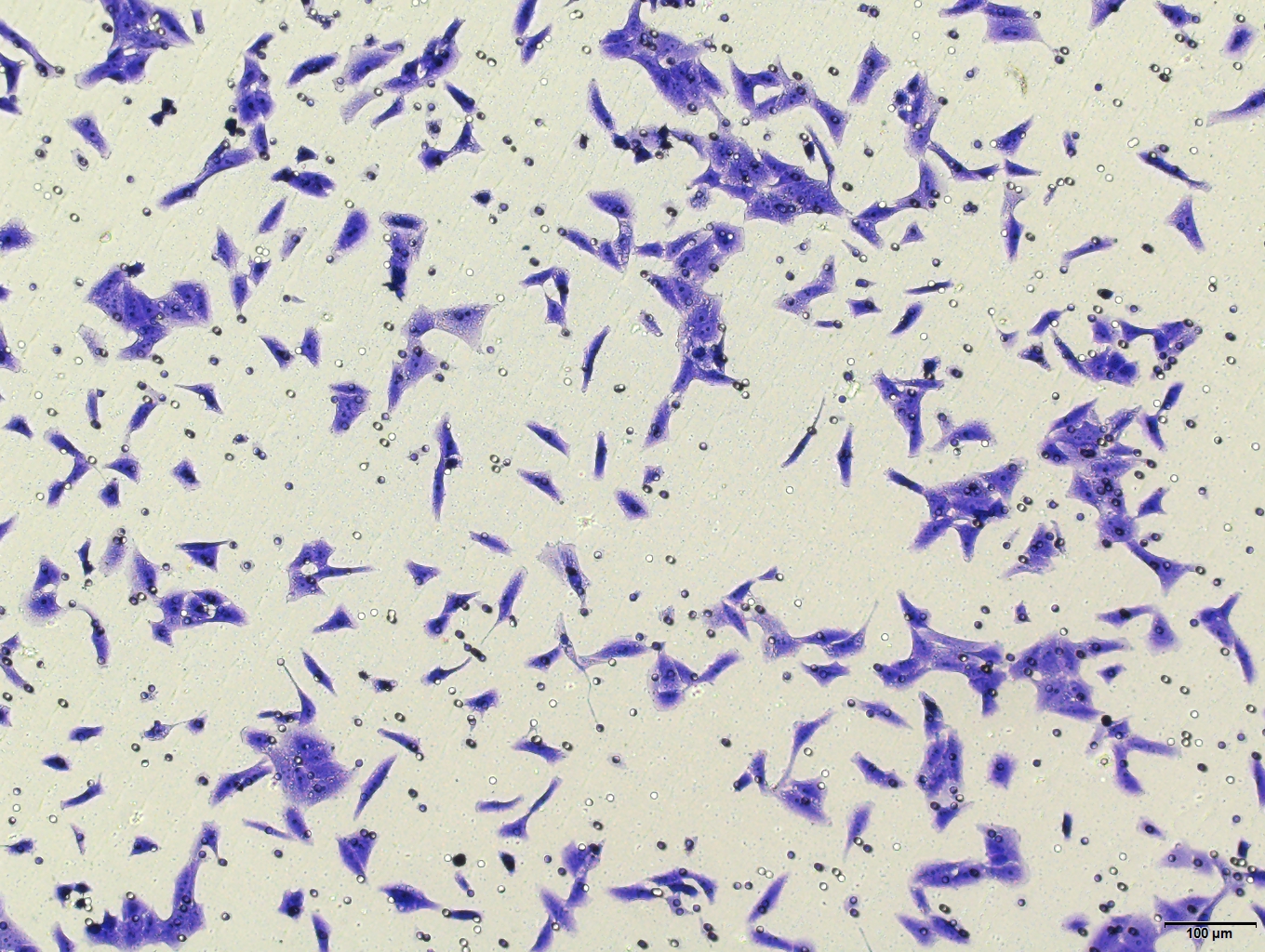

Supplement: Supplementary file 1 [file DataSheet1.zip › Figure 2 Excel/E/Huh7/Migration/shLINC01572#2.jpg]

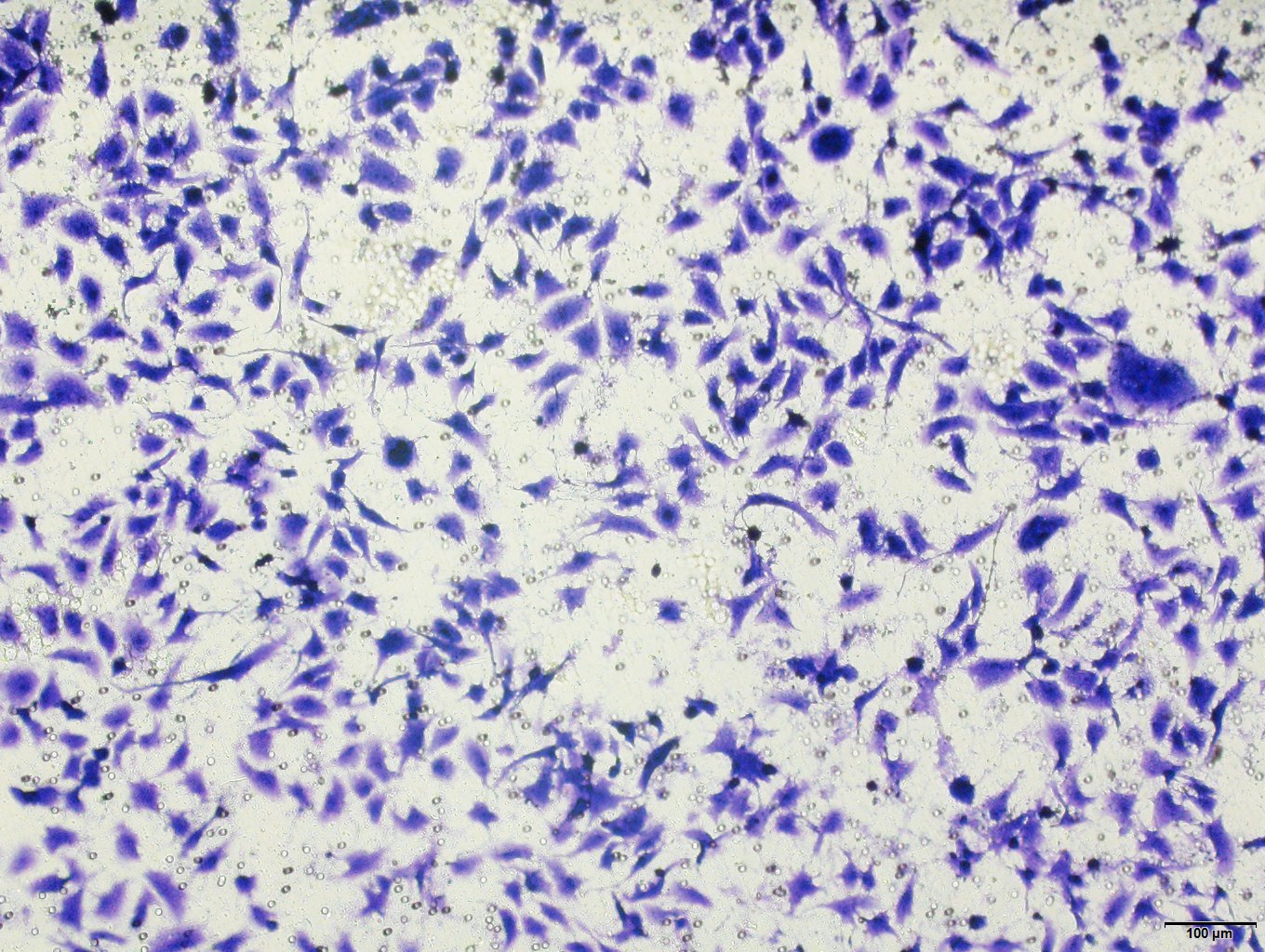

Supplement: Supplementary file 1 [file DataSheet1.zip › Figure 2 Excel/E/SNU-449/Invasion/shCtrl.jpg]

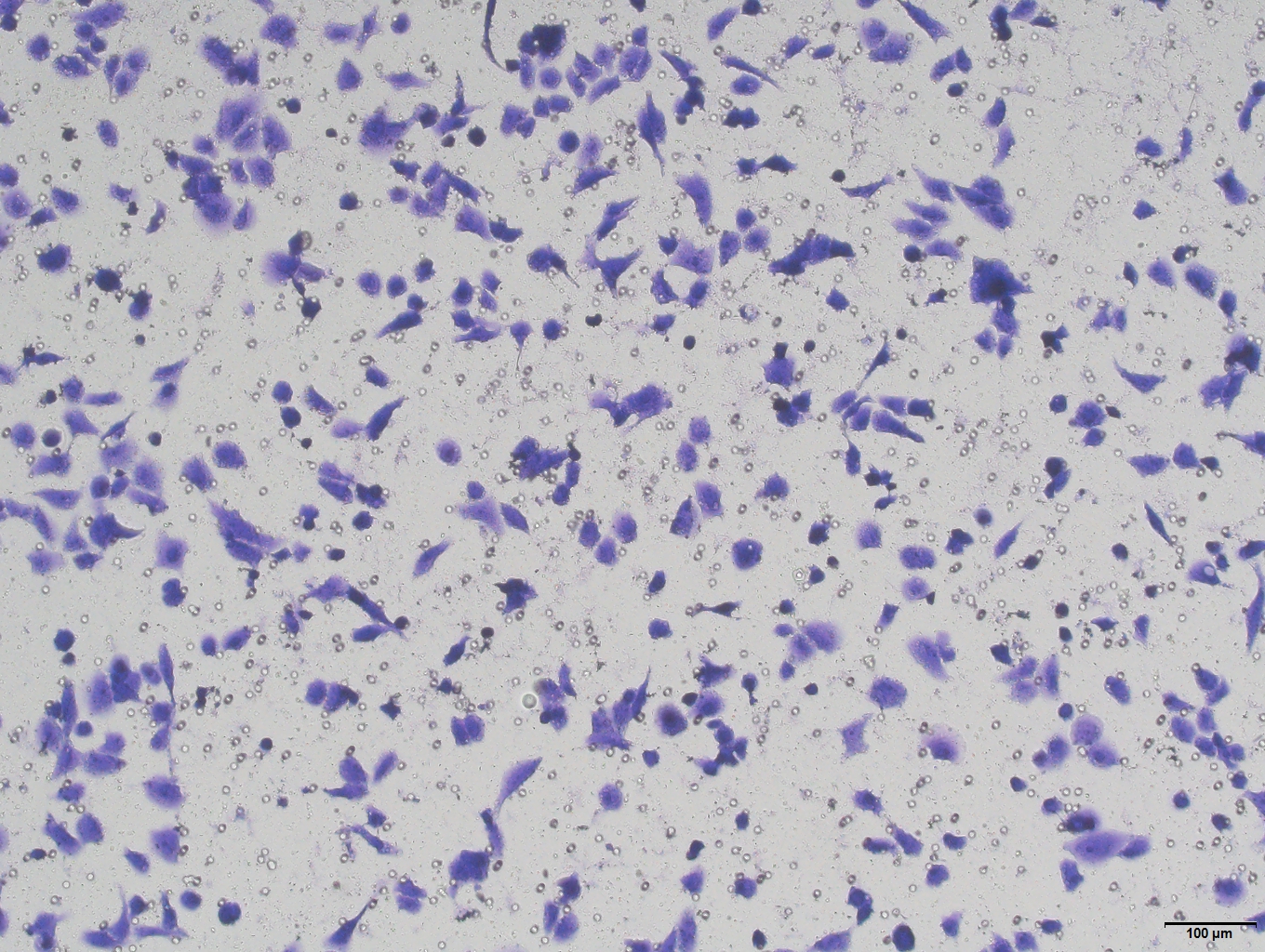

Supplement: Supplementary file 1 [file DataSheet1.zip › Figure 2 Excel/E/SNU-449/Invasion/shLINC01572#1.jpg]

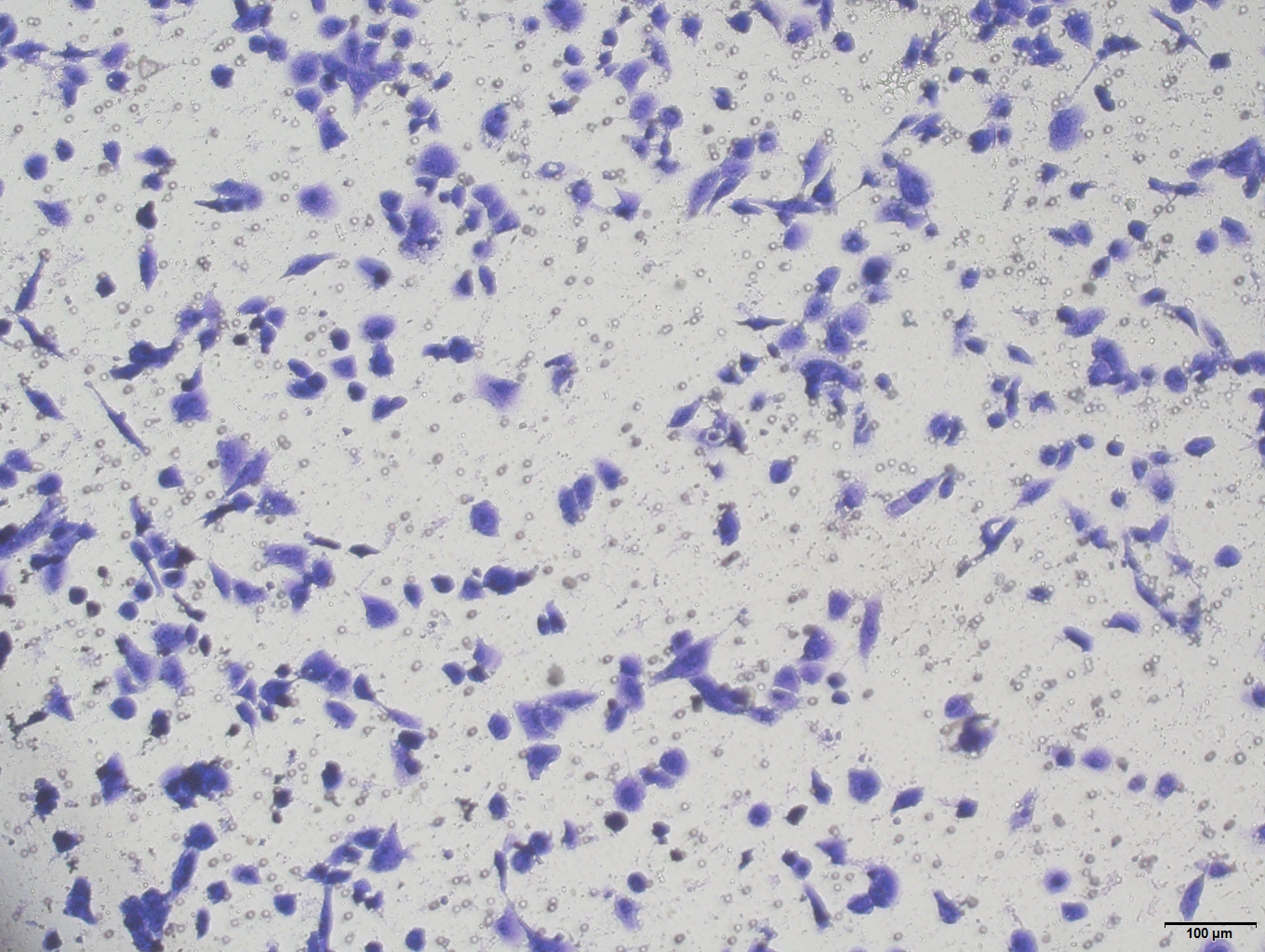

Supplement: Supplementary file 1 [file DataSheet1.zip › Figure 2 Excel/E/SNU-449/Invasion/shLINC01572#2.jpg]

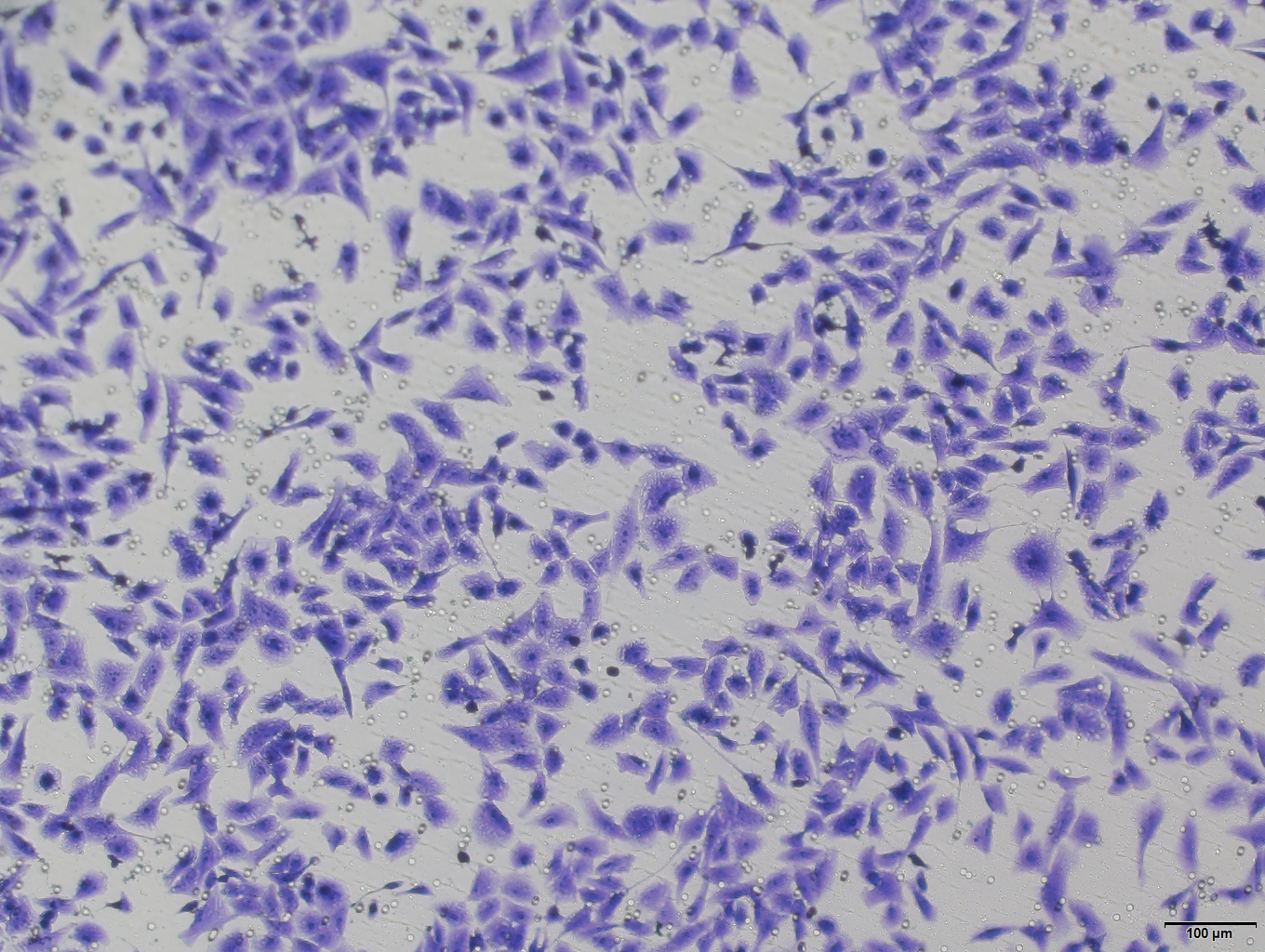

Supplement: Supplementary file 1 [file DataSheet1.zip › Figure 2 Excel/E/SNU-449/Migration/shCtrl.jpg]

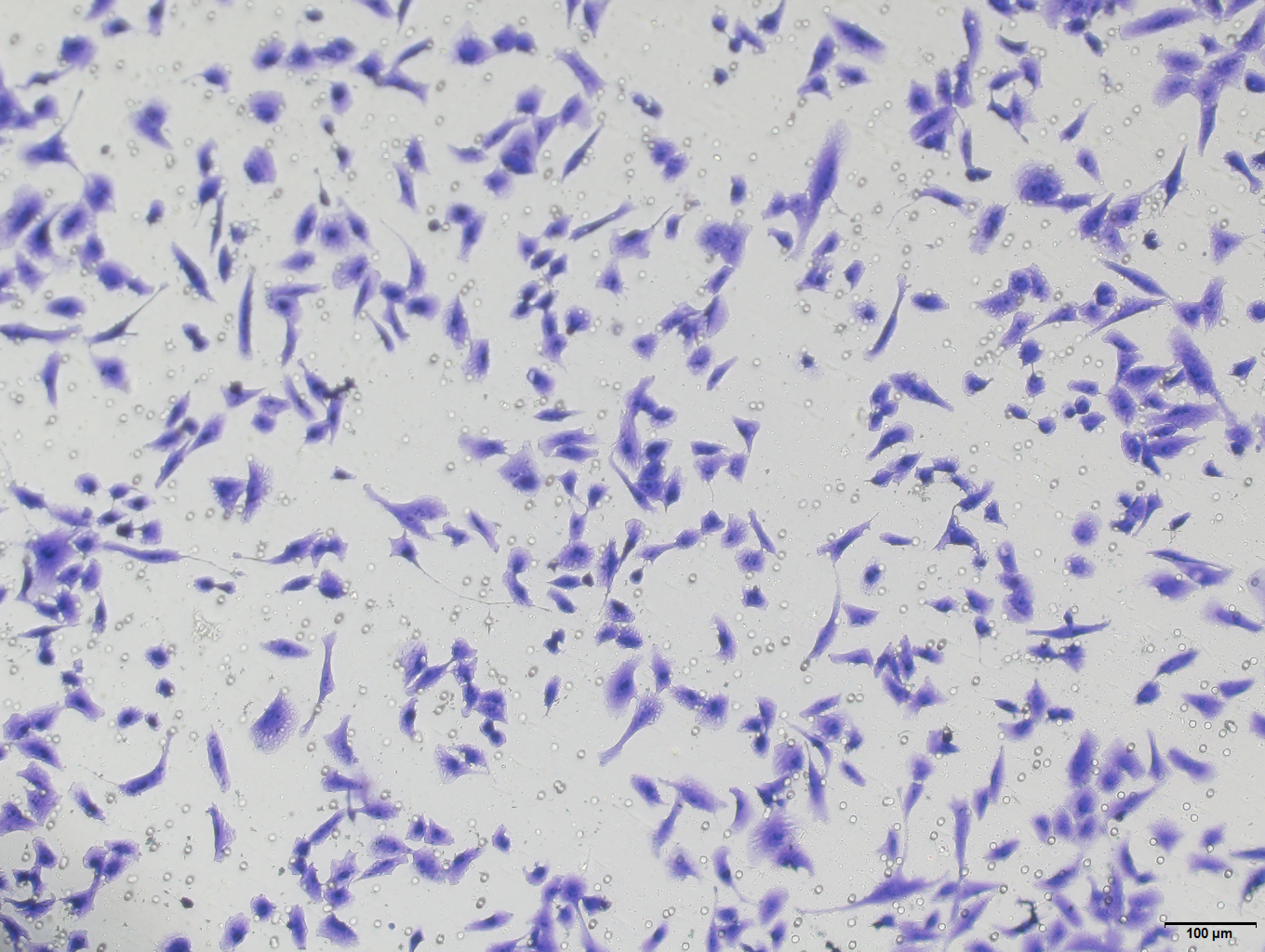

Supplement: Supplementary file 1 [file DataSheet1.zip › Figure 2 Excel/E/SNU-449/Migration/shLINC01572#1.jpg]

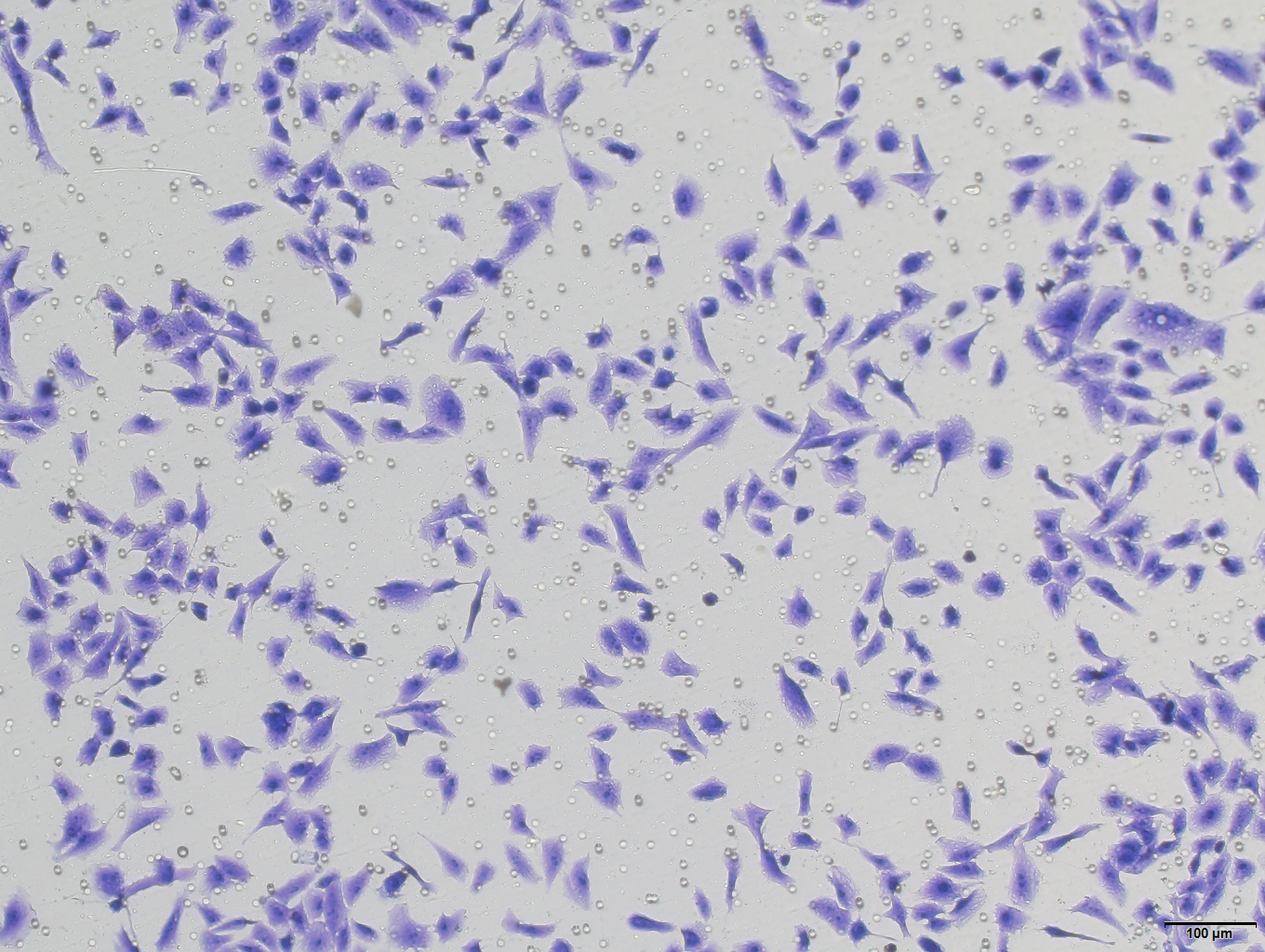

Supplement: Supplementary file 1 [file DataSheet1.zip › Figure 2 Excel/E/SNU-449/Migration/shLINC01572#2.jpg]

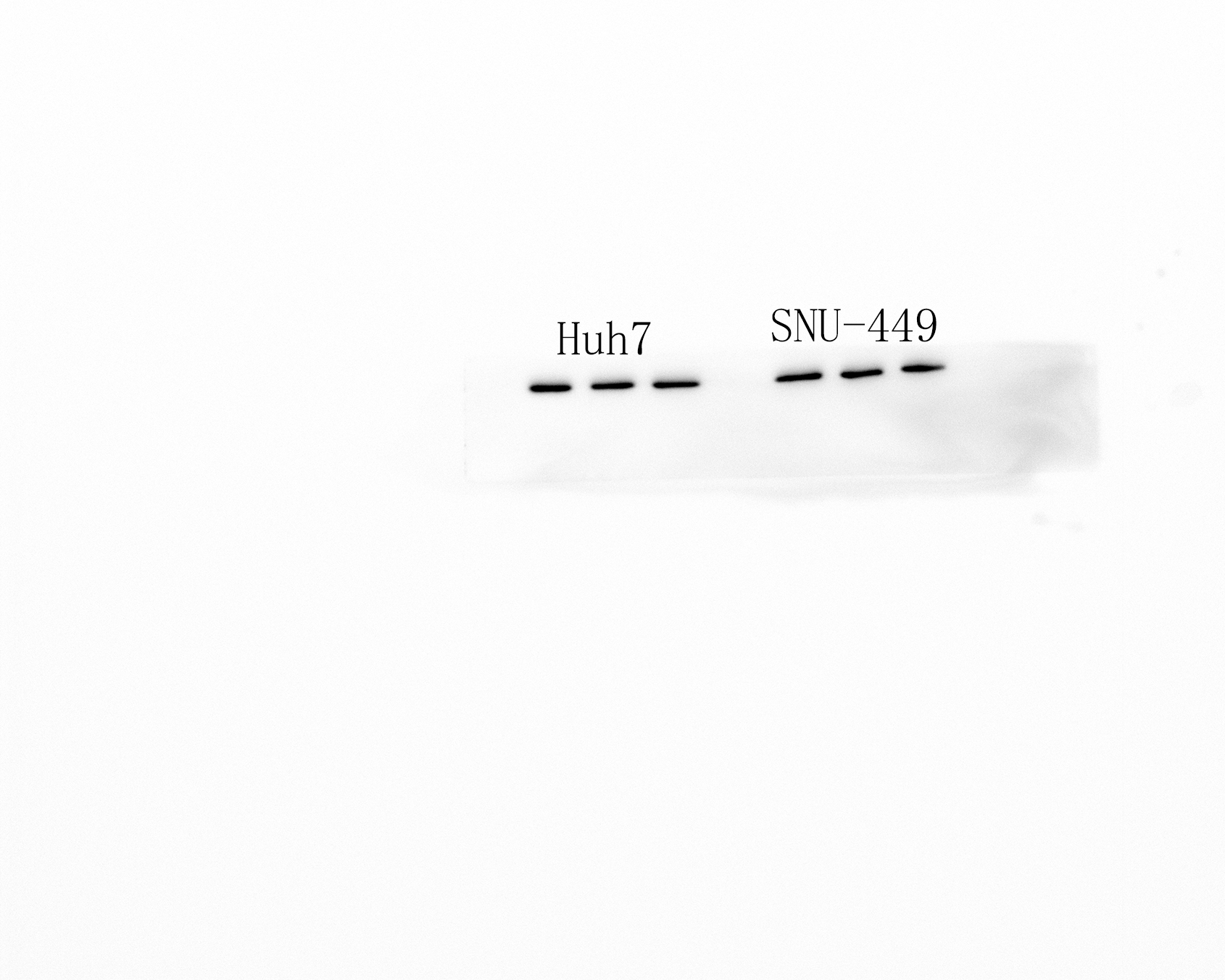

Supplement: Supplementary file 1 [file DataSheet1.zip › Figure 2 Excel/F WB/a--actin/a--actin 1.jpg]

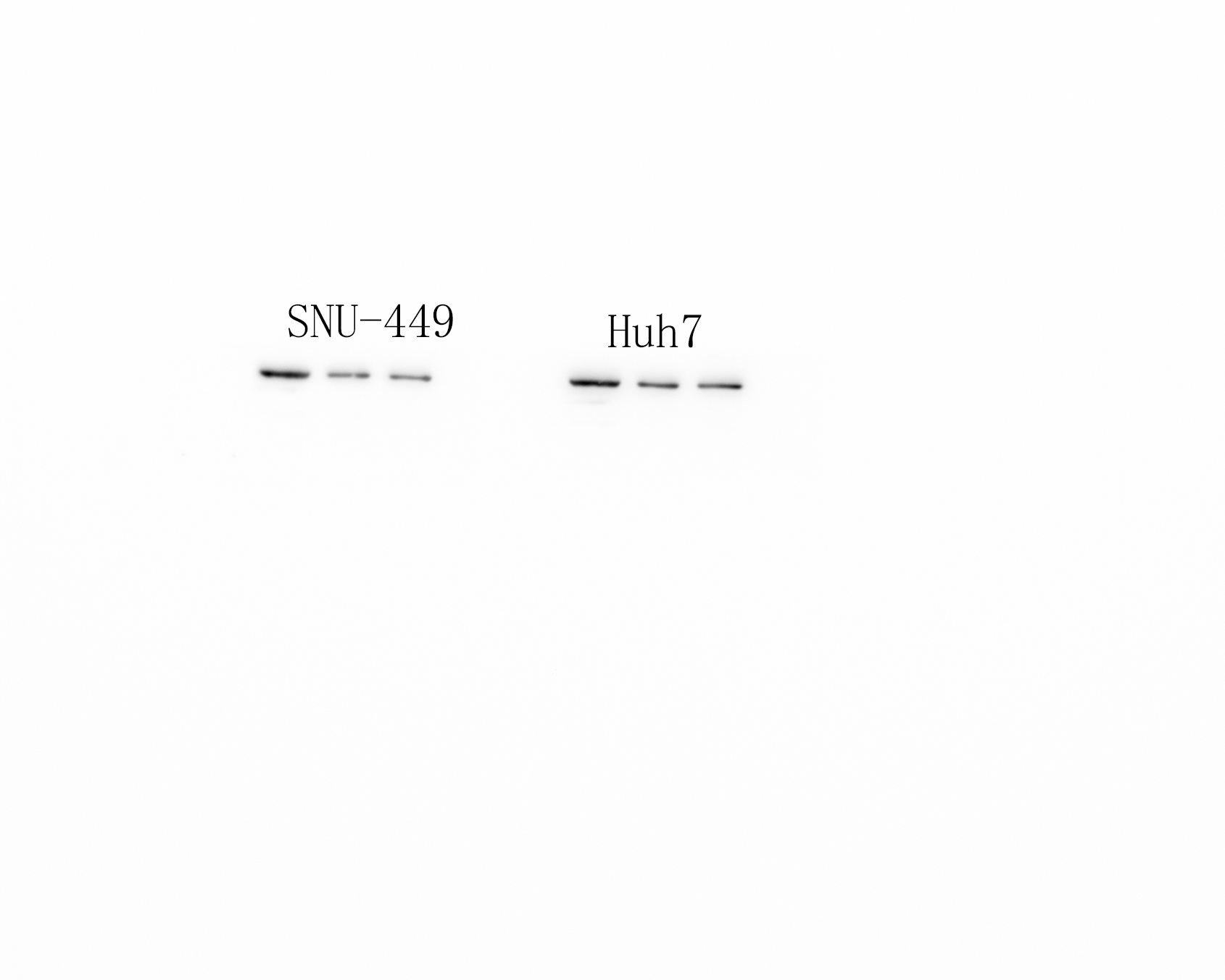

Supplement: Supplementary file 1 [file DataSheet1.zip › Figure 2 Excel/F WB/a--Catenin/a--Catenin 1.jpg]

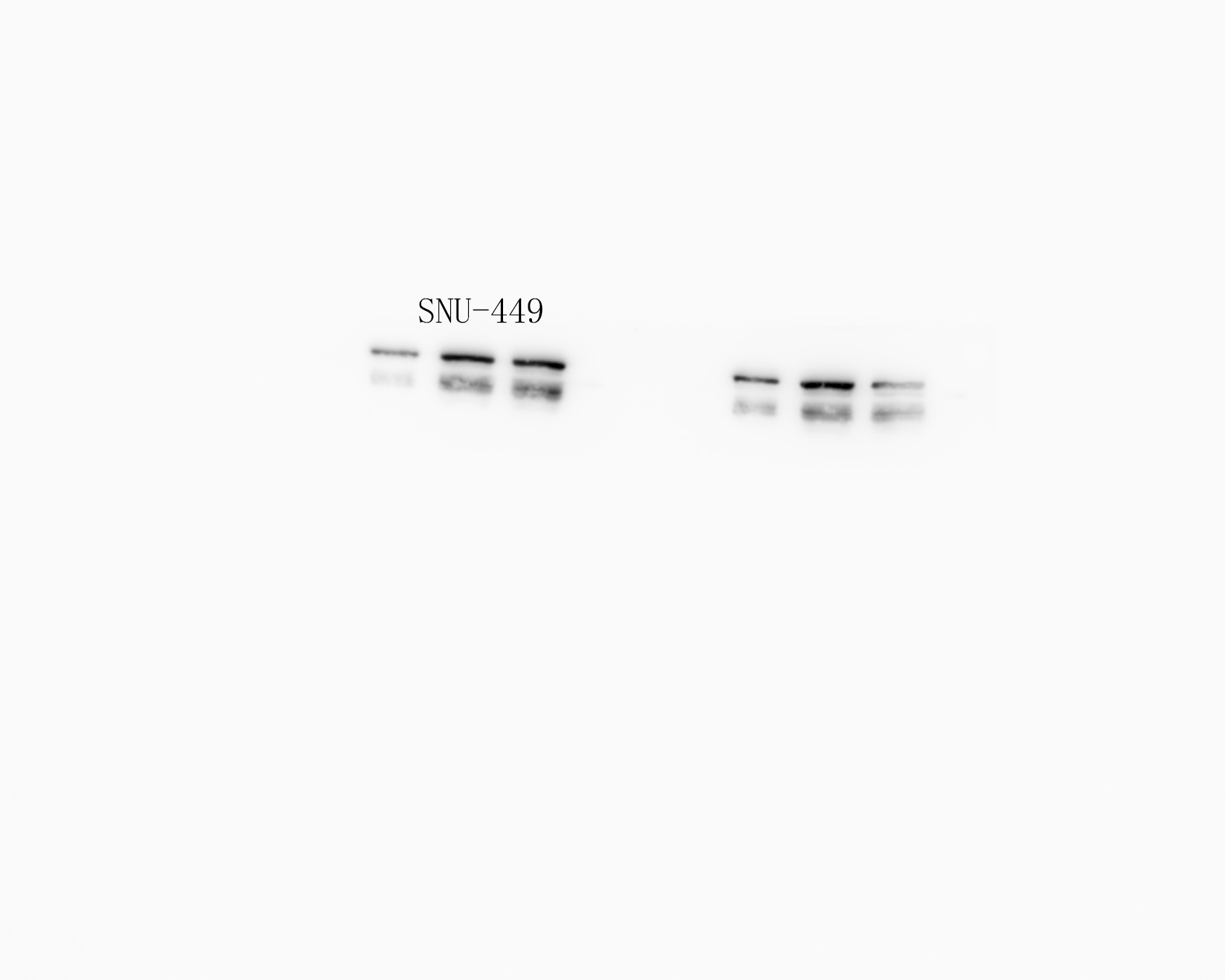

Supplement: Supplementary file 1 [file DataSheet1.zip › Figure 2 Excel/F WB/E-cadherin/E-cadherin 1.jpg]

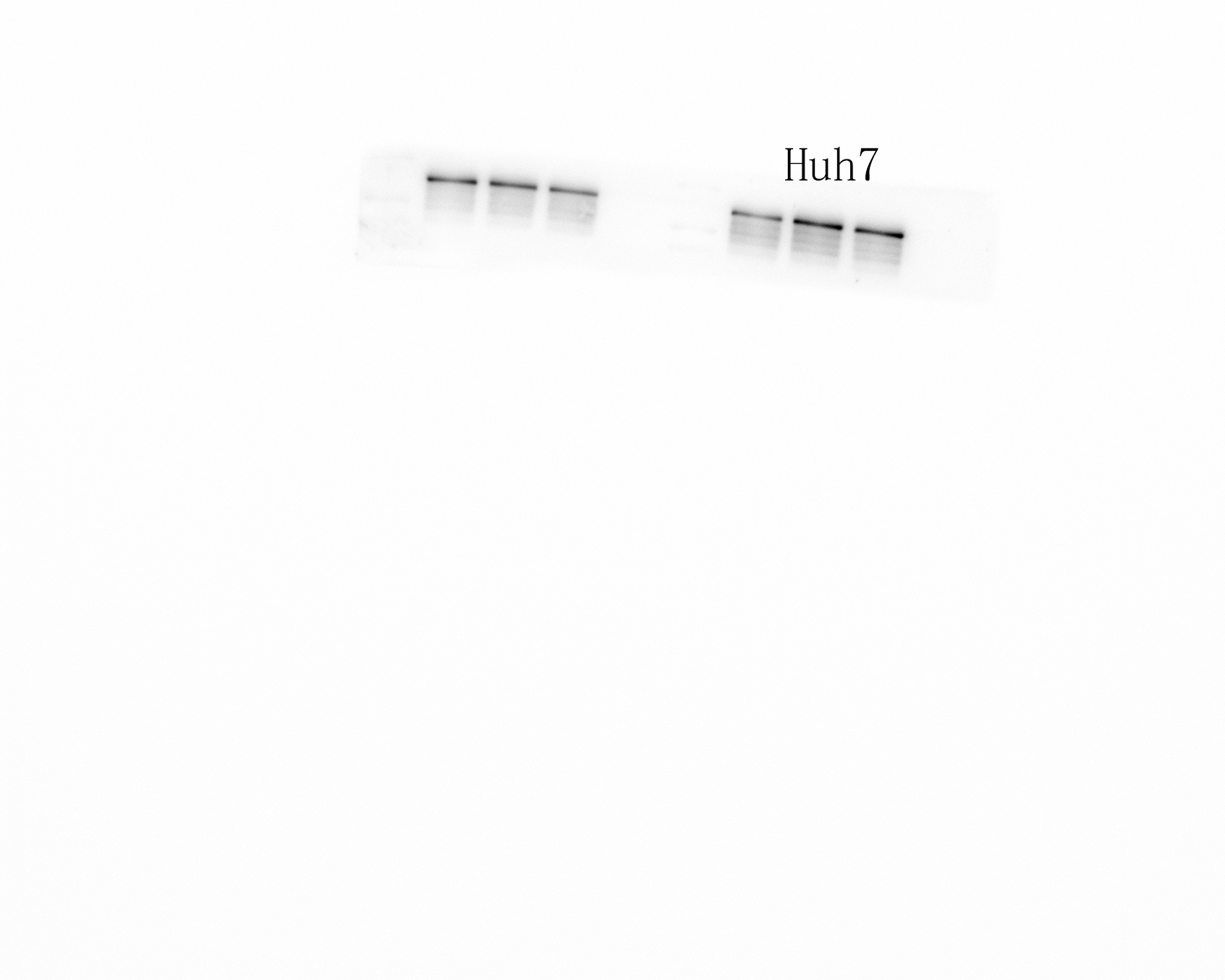

Supplement: Supplementary file 1 [file DataSheet1.zip › Figure 2 Excel/F WB/E-cadherin/E-cadherin 2.jpg]

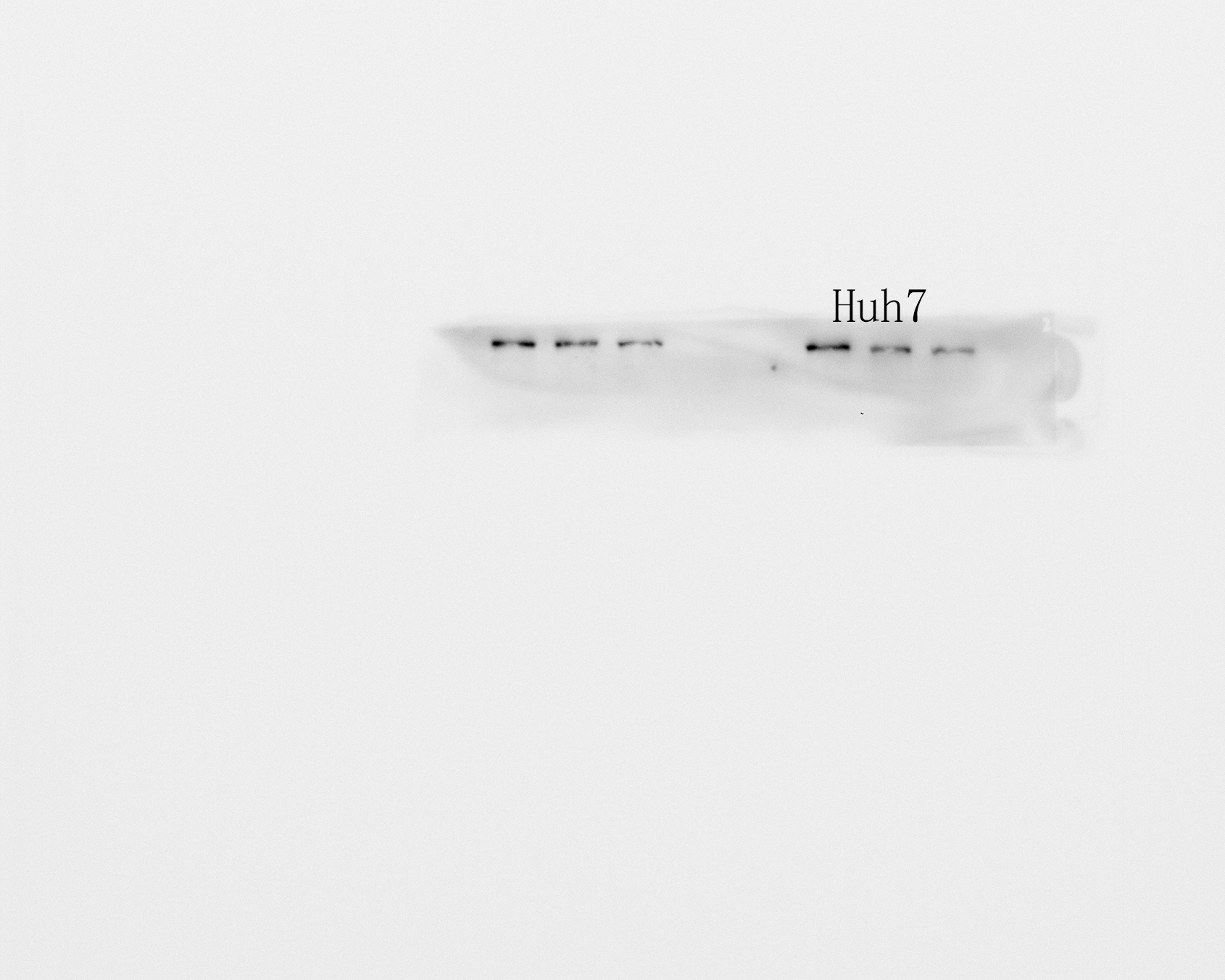

Supplement: Supplementary file 1 [file DataSheet1.zip › Figure 2 Excel/F WB/N-cadherin/N-cadherin 1.jpg]

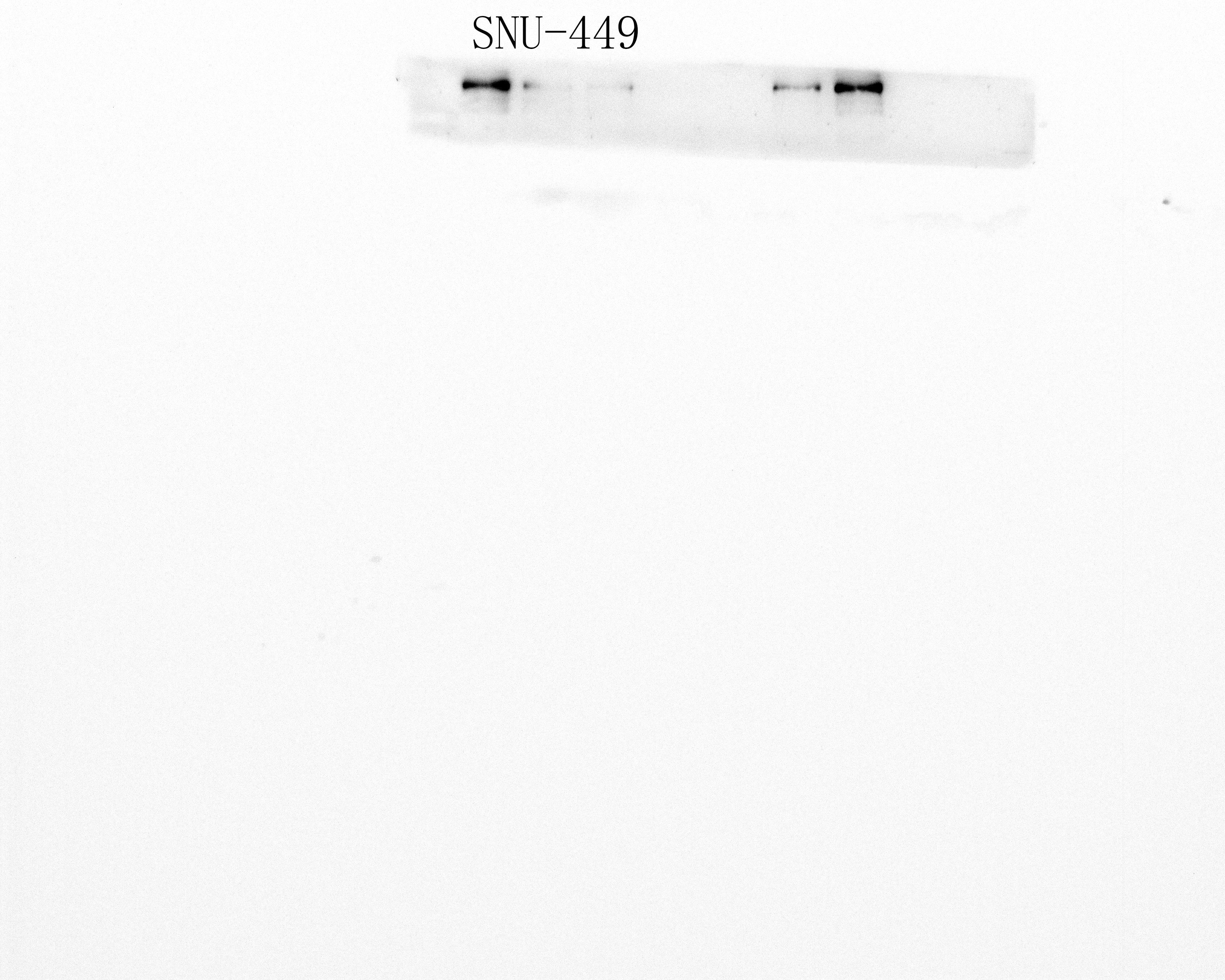

Supplement: Supplementary file 1 [file DataSheet1.zip › Figure 2 Excel/F WB/N-cadherin/N-cadherin 2.jpg]

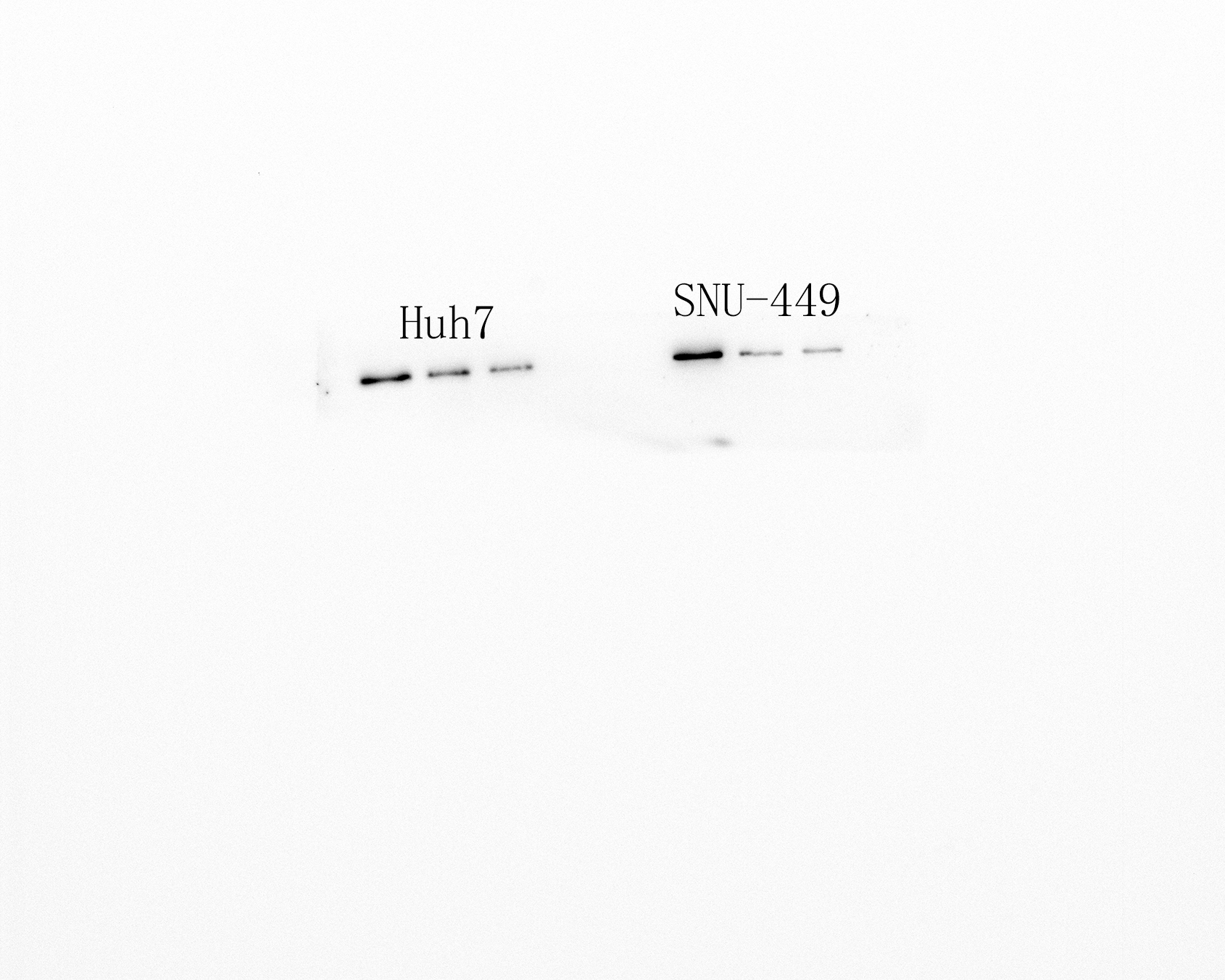

Supplement: Supplementary file 1 [file DataSheet1.zip › Figure 2 Excel/F WB/Vimentin/Vimentin 1.jpg]

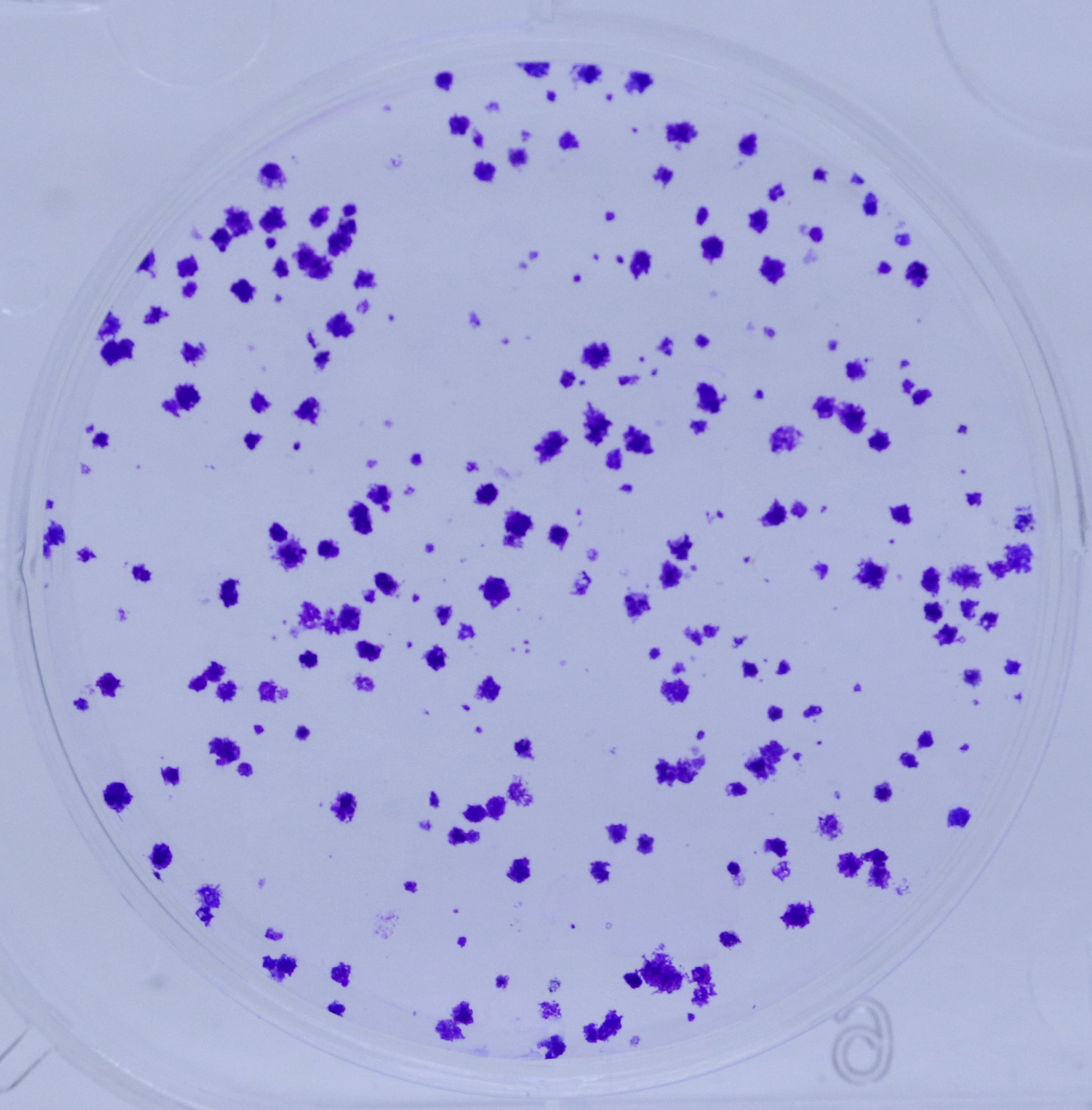

Supplement: Supplementary file 1 [file DataSheet1.zip › Figure 3 Excel/C/LINC01572.JPG]

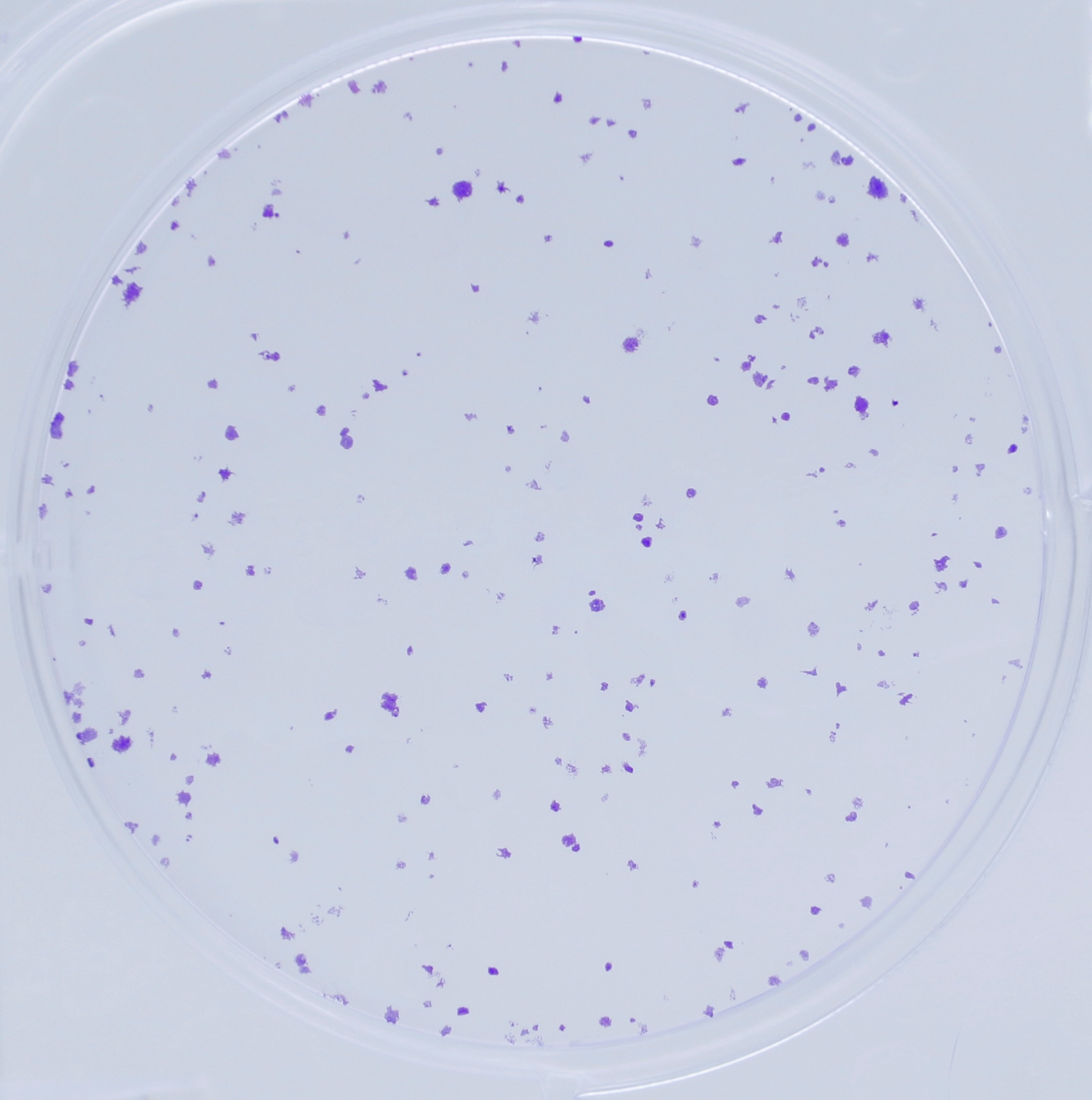

Supplement: Supplementary file 1 [file DataSheet1.zip › Figure 3 Excel/C/NC.JPG]

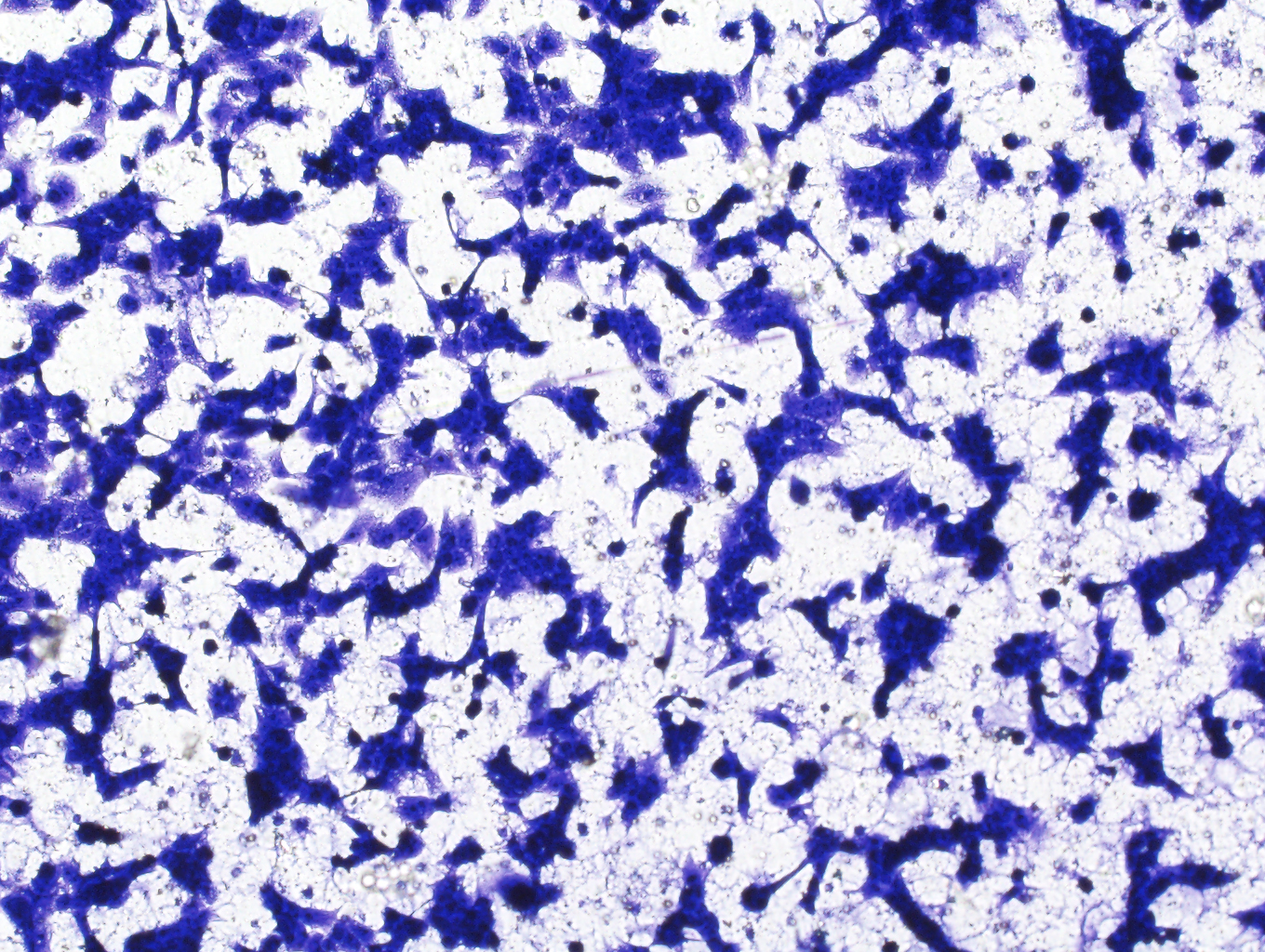

Supplement: Supplementary file 1 [file DataSheet1.zip › Figure 3 Excel/D/Invasion/LINC01572.jpg]

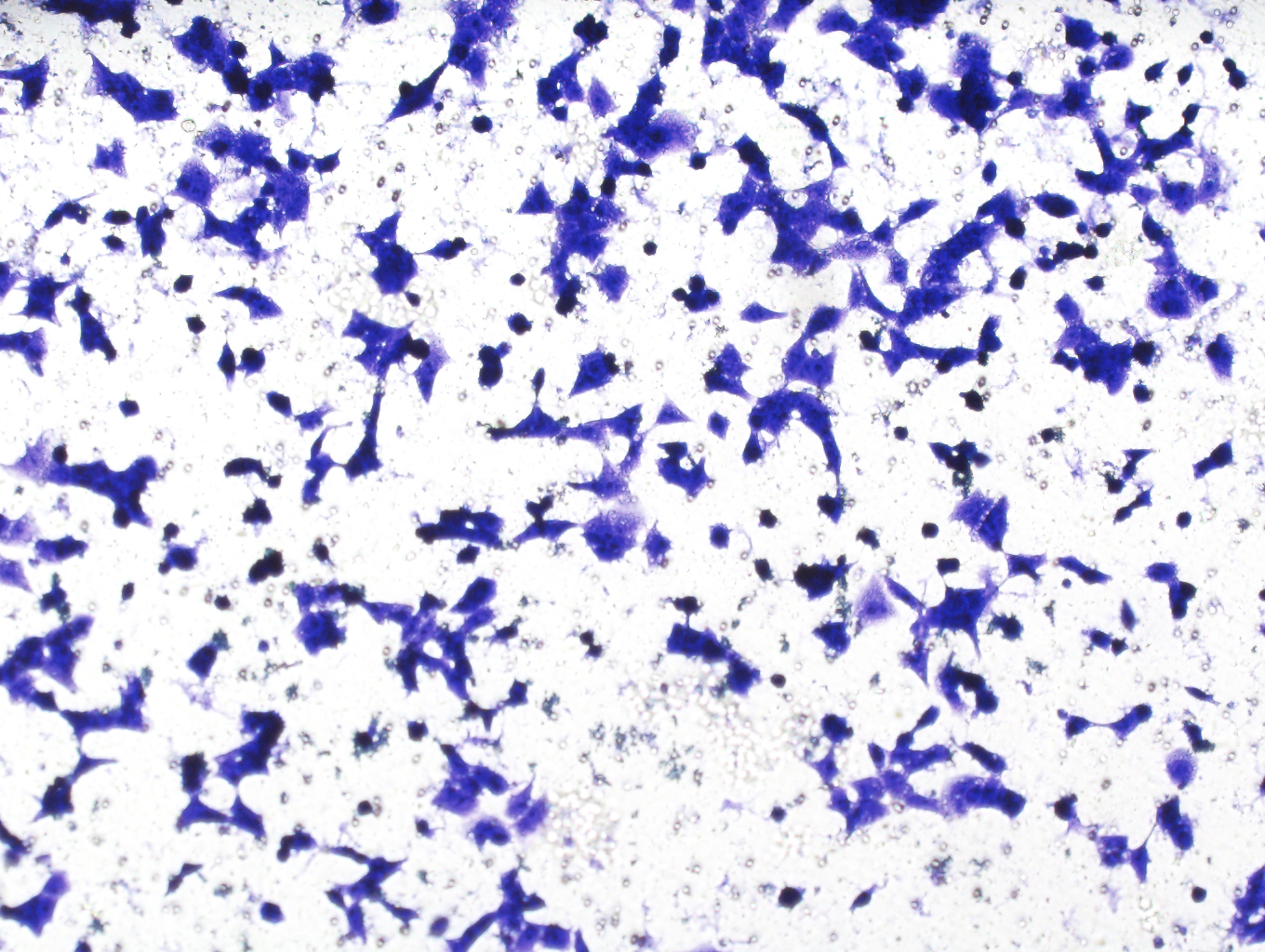

Supplement: Supplementary file 1 [file DataSheet1.zip › Figure 3 Excel/D/Invasion/NC.jpg]

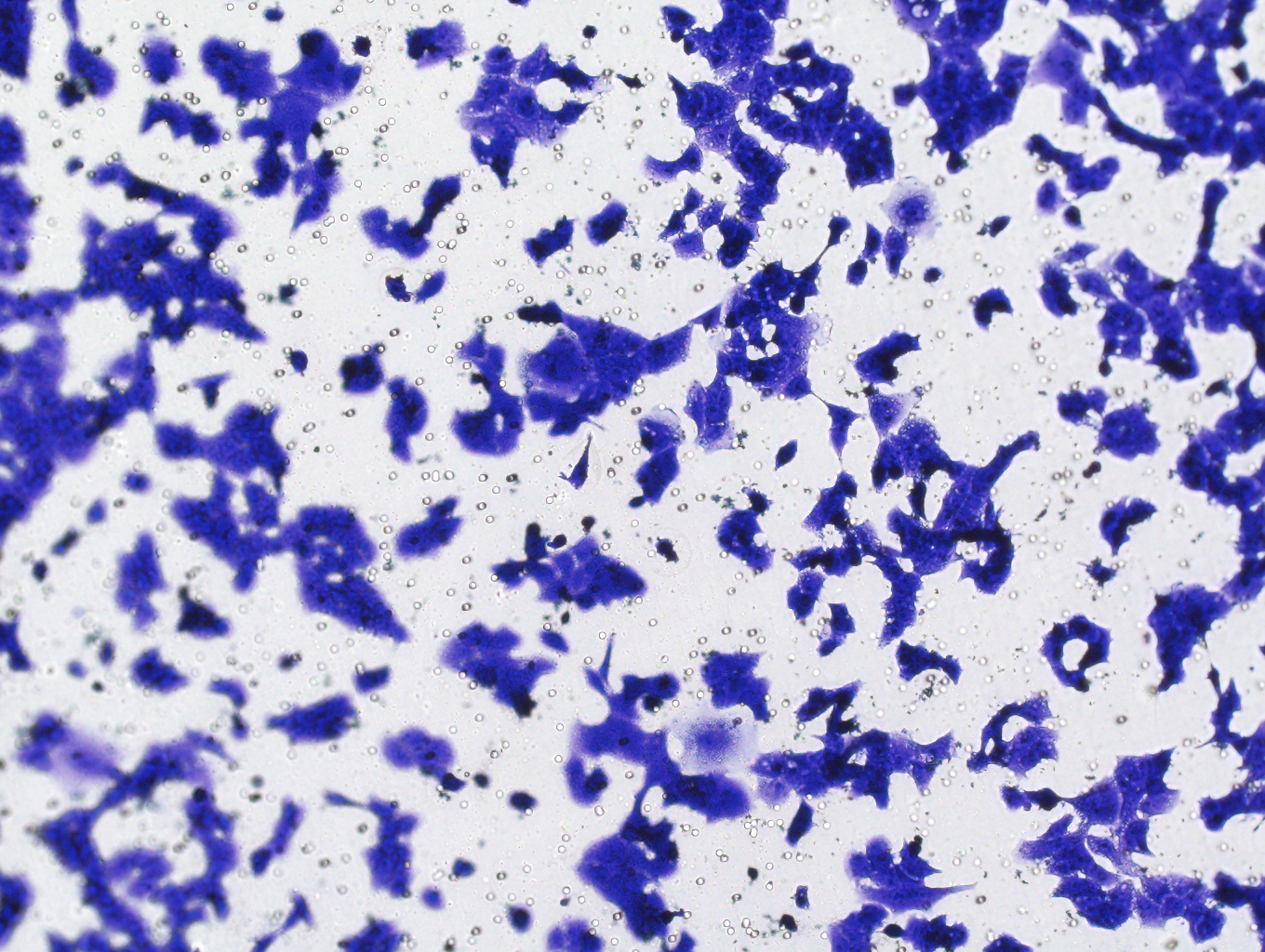

Supplement: Supplementary file 1 [file DataSheet1.zip › Figure 3 Excel/D/Migration/LINC01572.jpg]

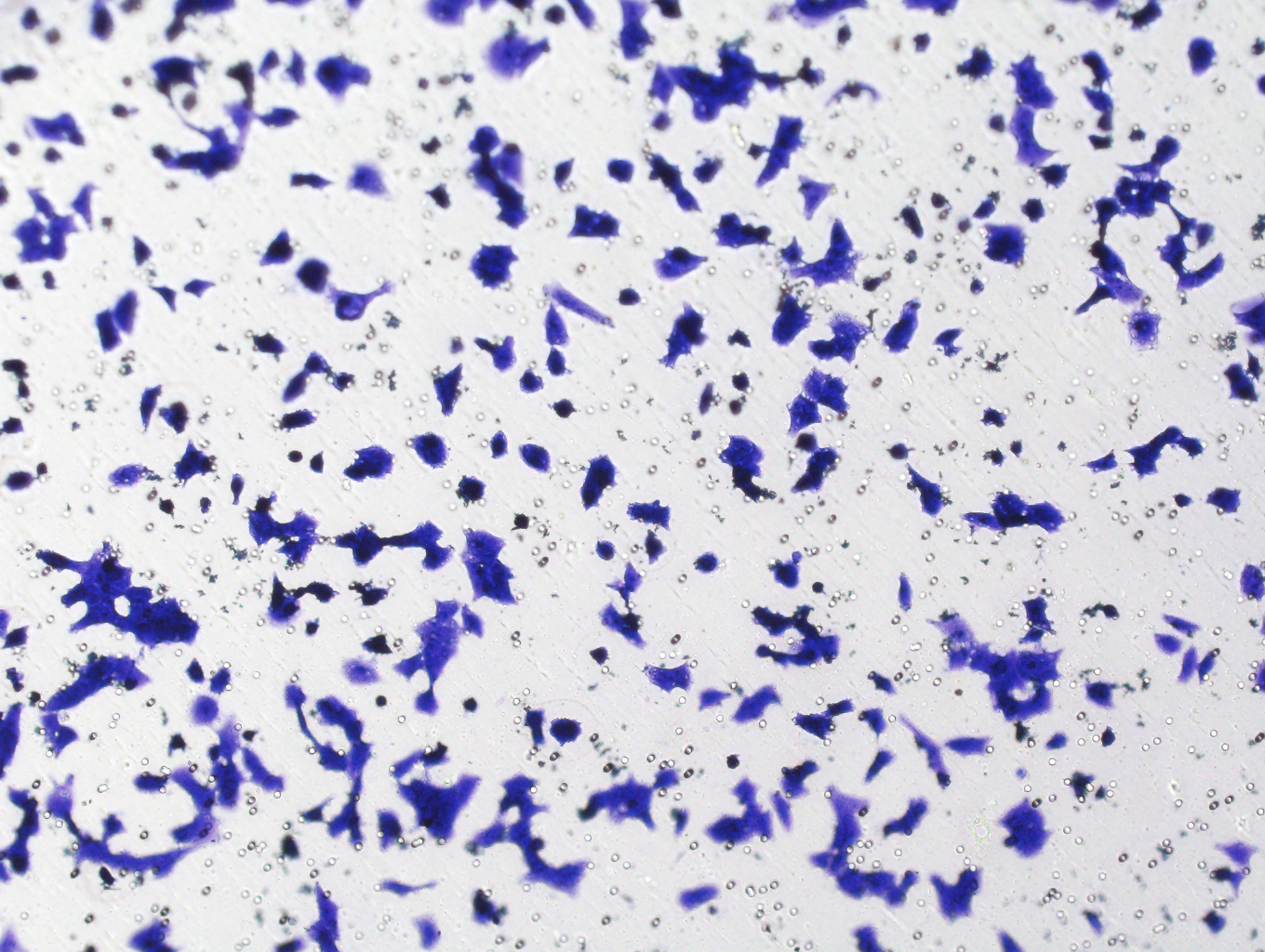

Supplement: Supplementary file 1 [file DataSheet1.zip › Figure 3 Excel/D/Migration/NC.jpg]

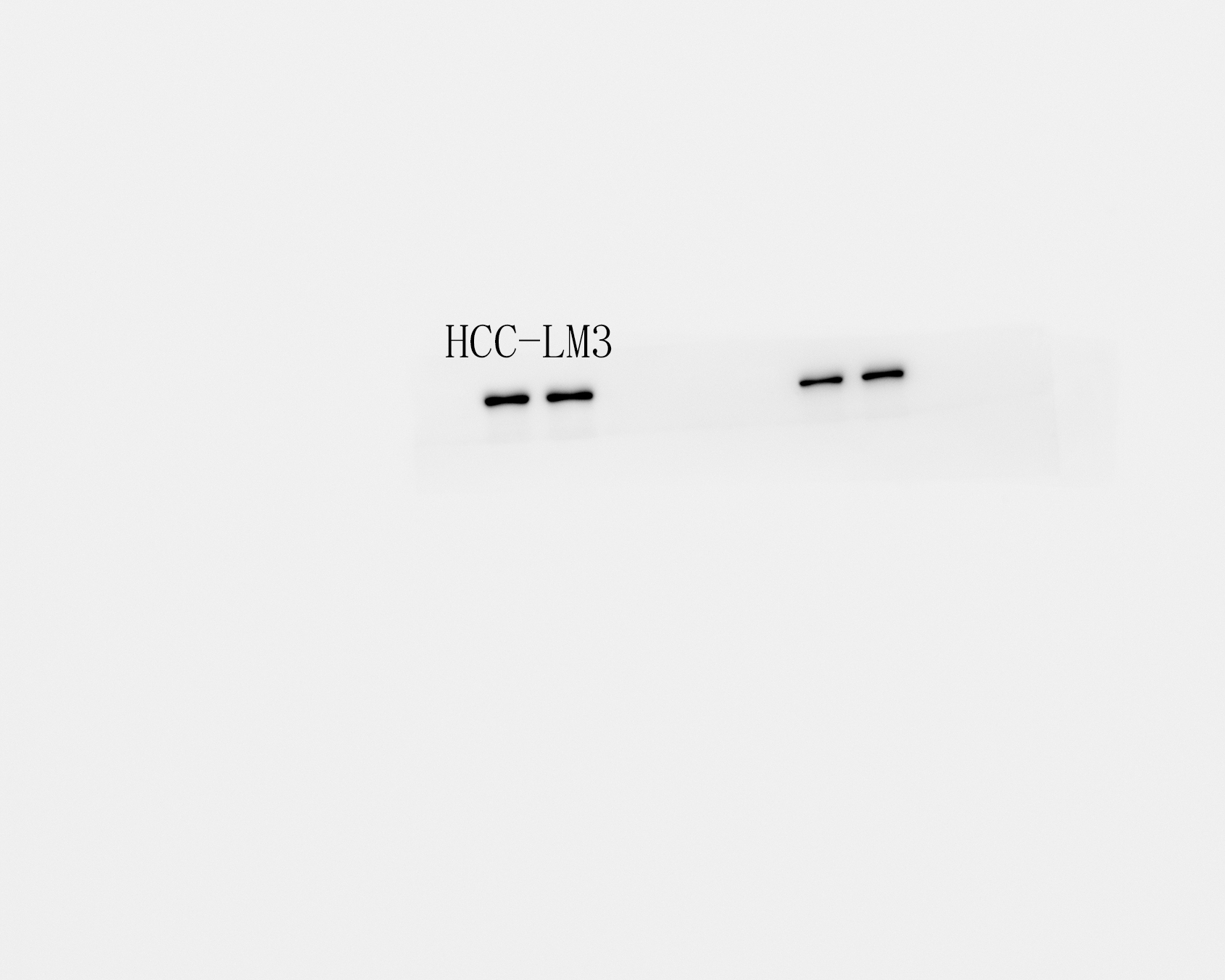

Supplement: Supplementary file 1 [file DataSheet1.zip › Figure 3 Excel/F/a--actin 2.jpg]

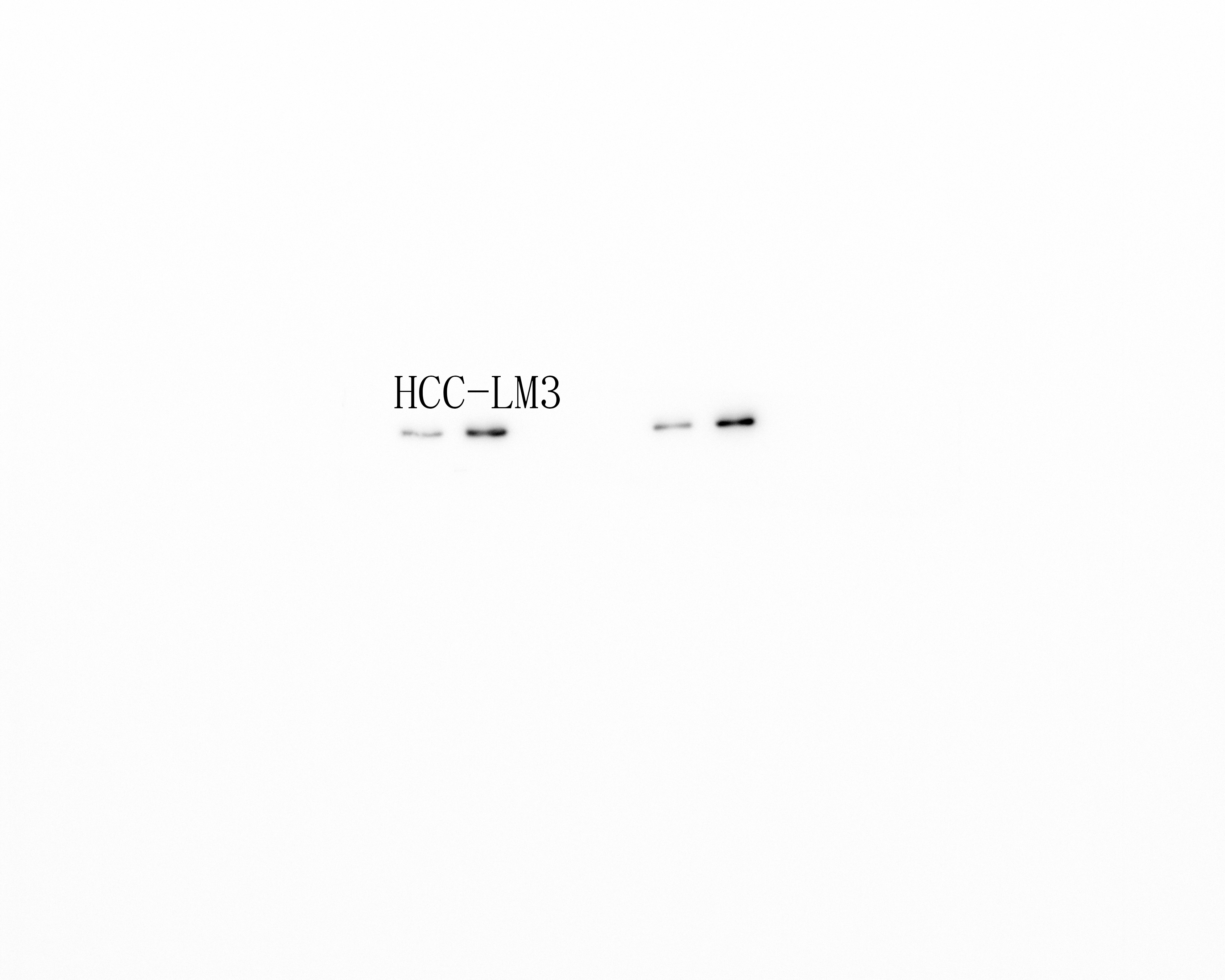

Supplement: Supplementary file 1 [file DataSheet1.zip › Figure 3 Excel/F/a--Catenin 2.jpg]

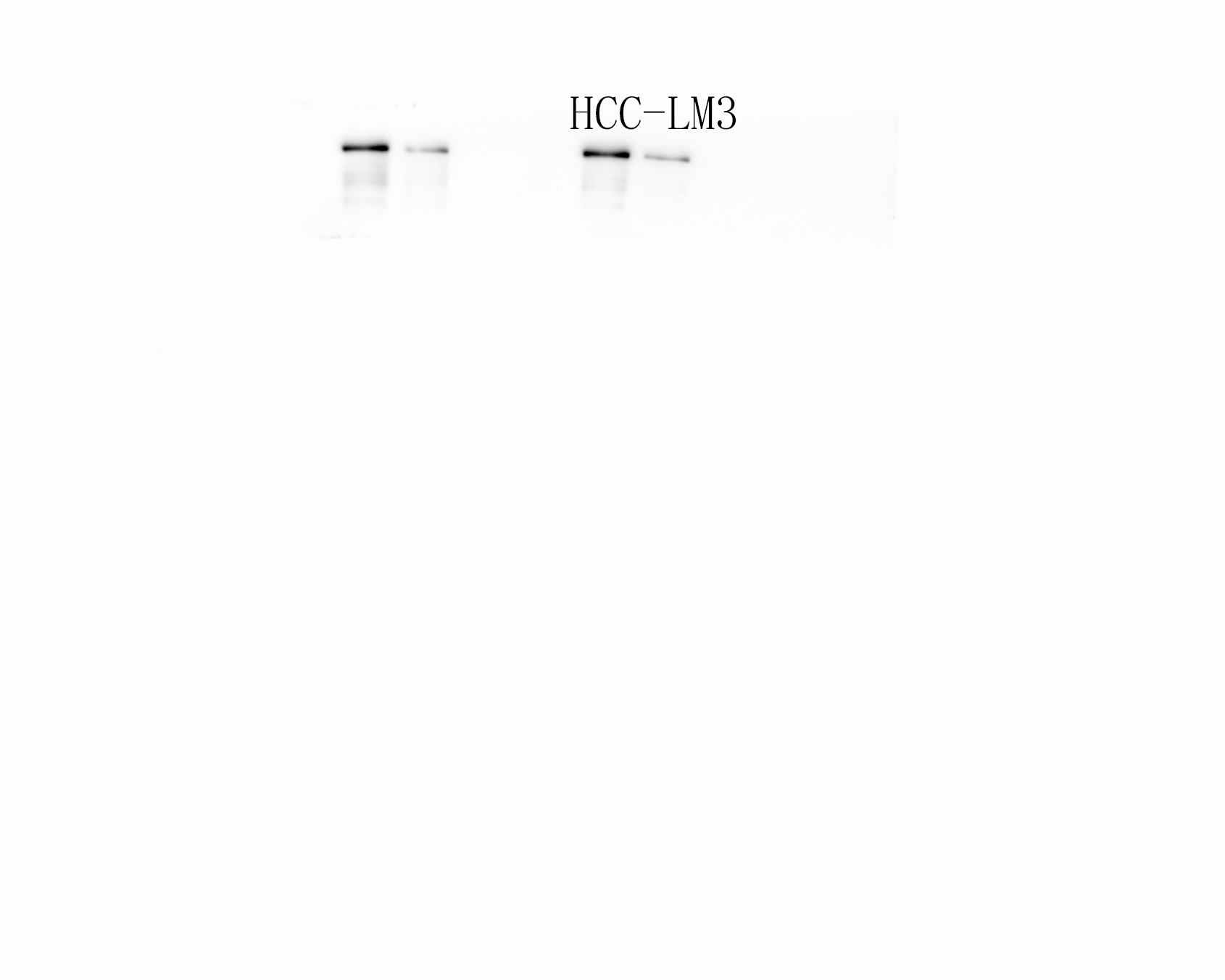

Supplement: Supplementary file 1 [file DataSheet1.zip › Figure 3 Excel/F/E-cadherin (2).jpg]

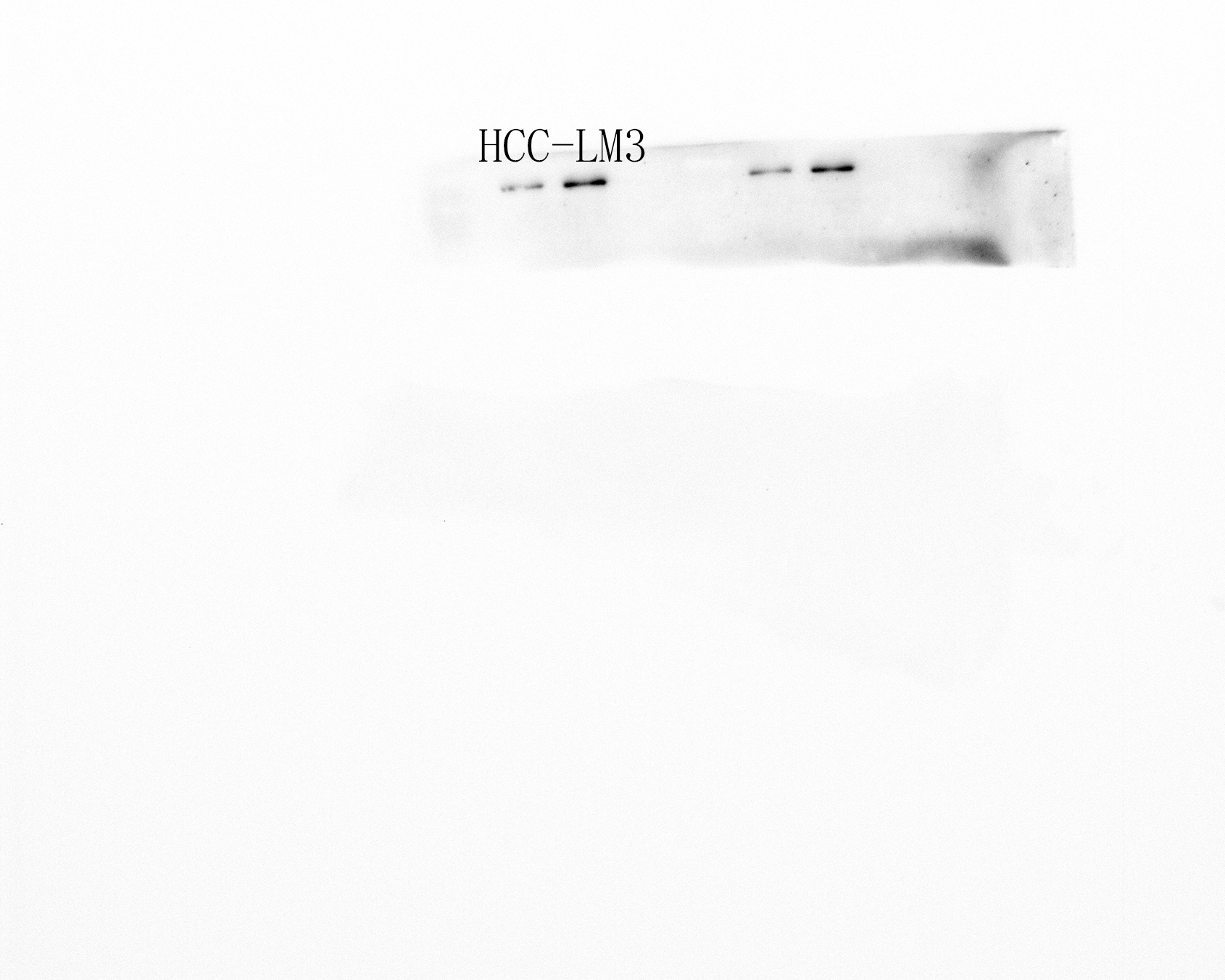

Supplement: Supplementary file 1 [file DataSheet1.zip › Figure 3 Excel/F/N-cadherin 2.jpg]

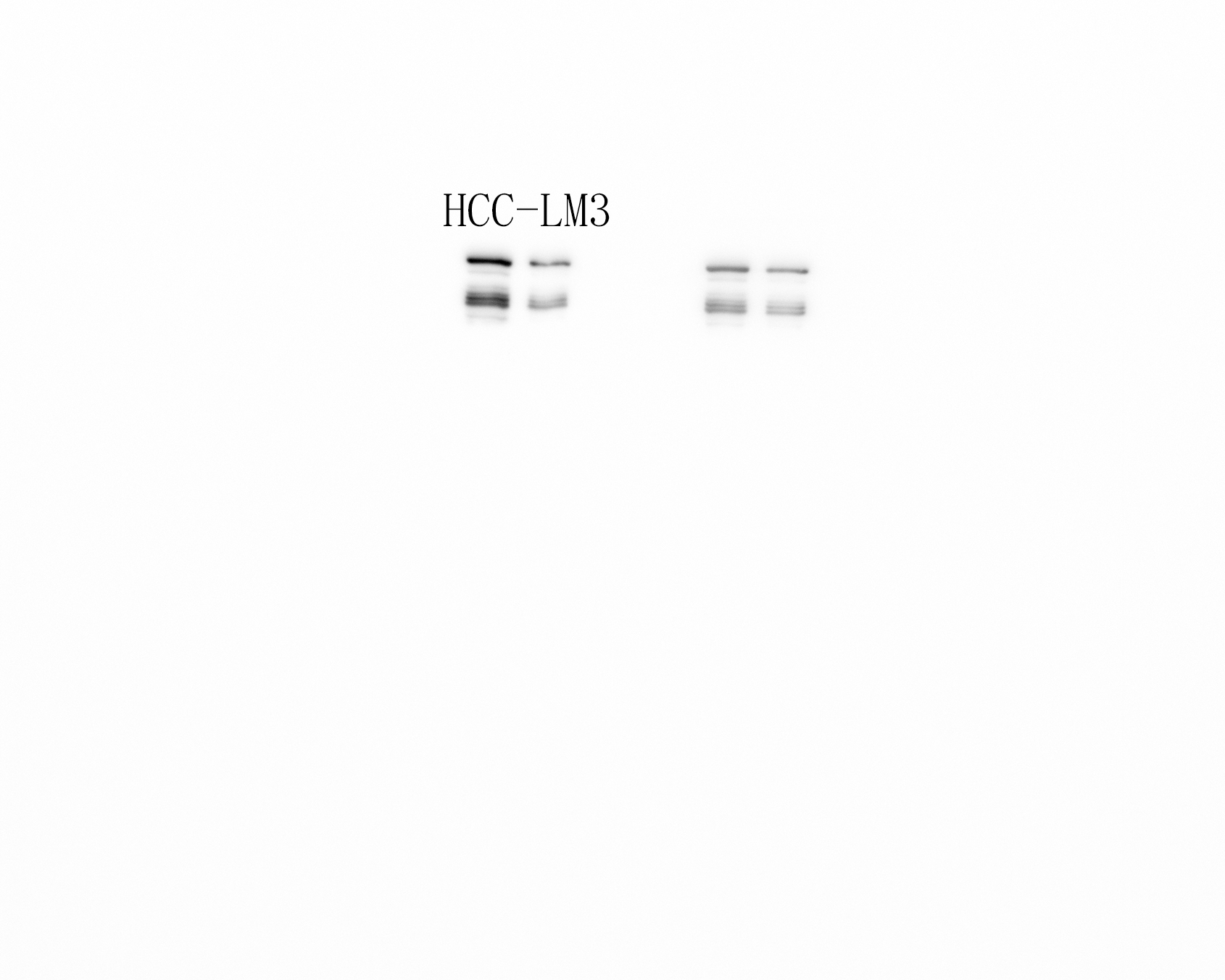

Supplement: Supplementary file 1 [file DataSheet1.zip › Figure 3 Excel/F/Vimentin 2.jpg]

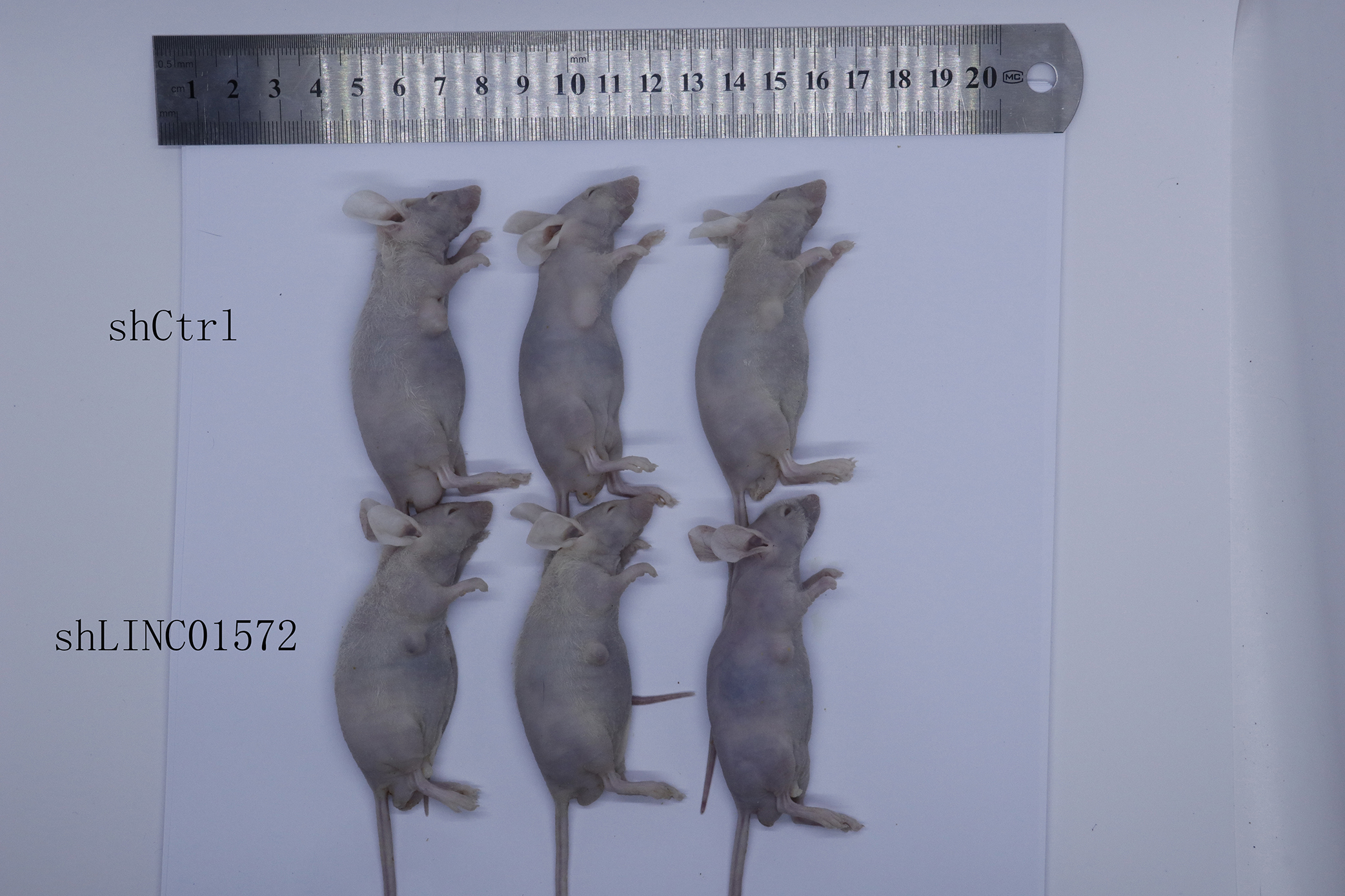

Supplement: Supplementary file 1 [file DataSheet1.zip › Figure 4 Excel/A/_MG_0685.JPG]

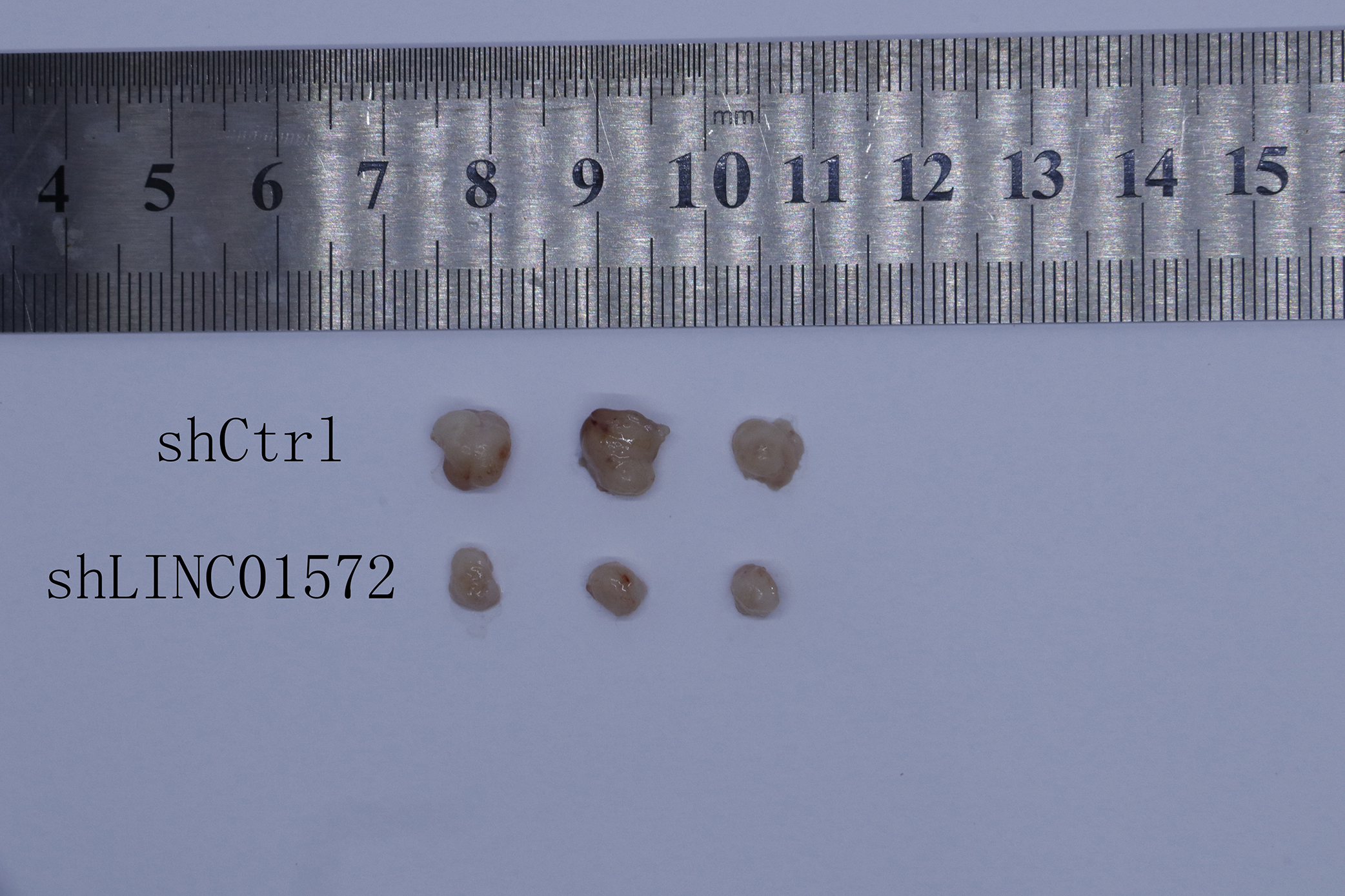

Supplement: Supplementary file 1 [file DataSheet1.zip › Figure 4 Excel/A/_MG_0700.JPG]

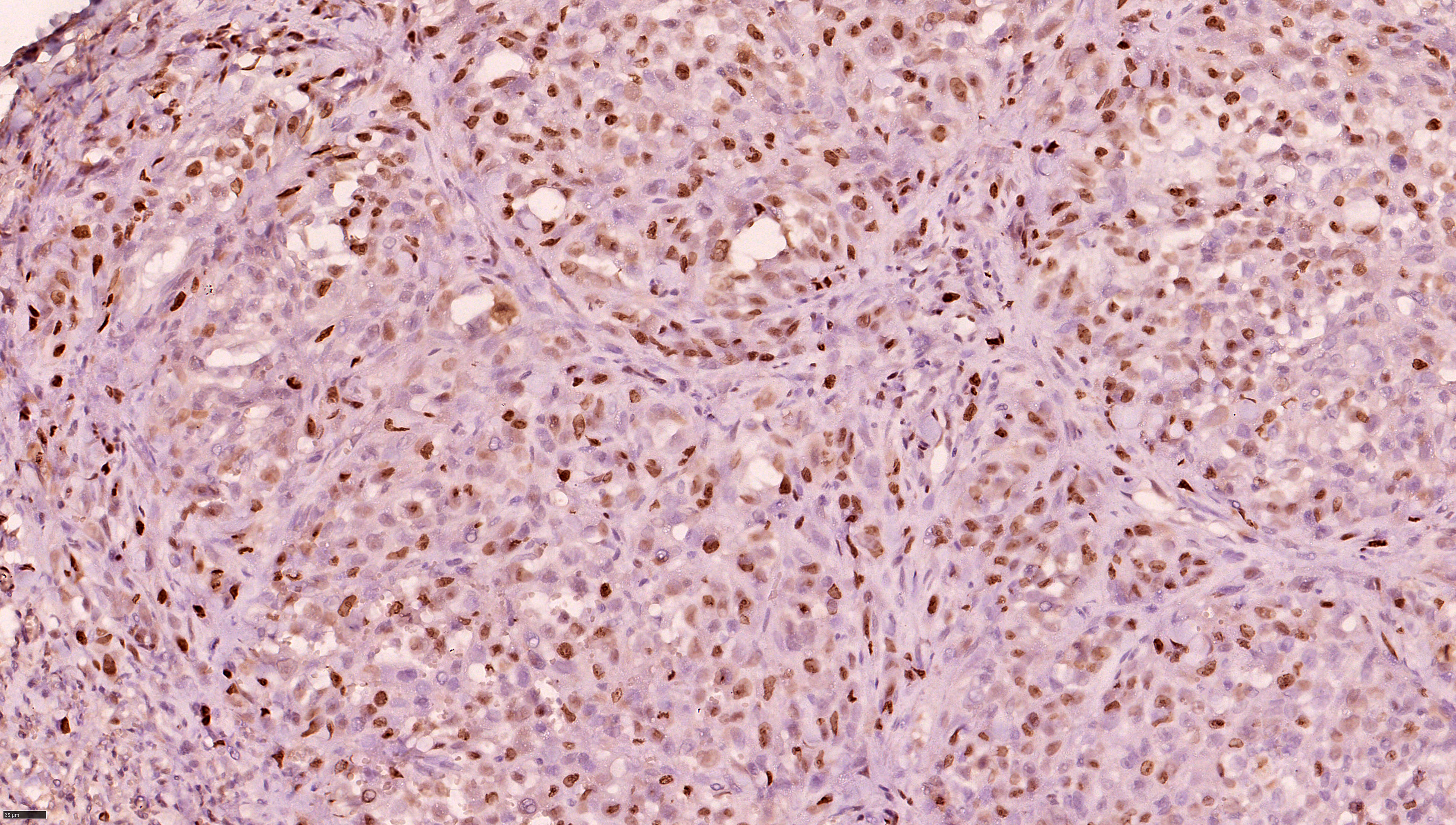

Supplement: Supplementary file 1 [file DataSheet1.zip › Figure 4 Excel/D/shCtrl 20X.jpg]

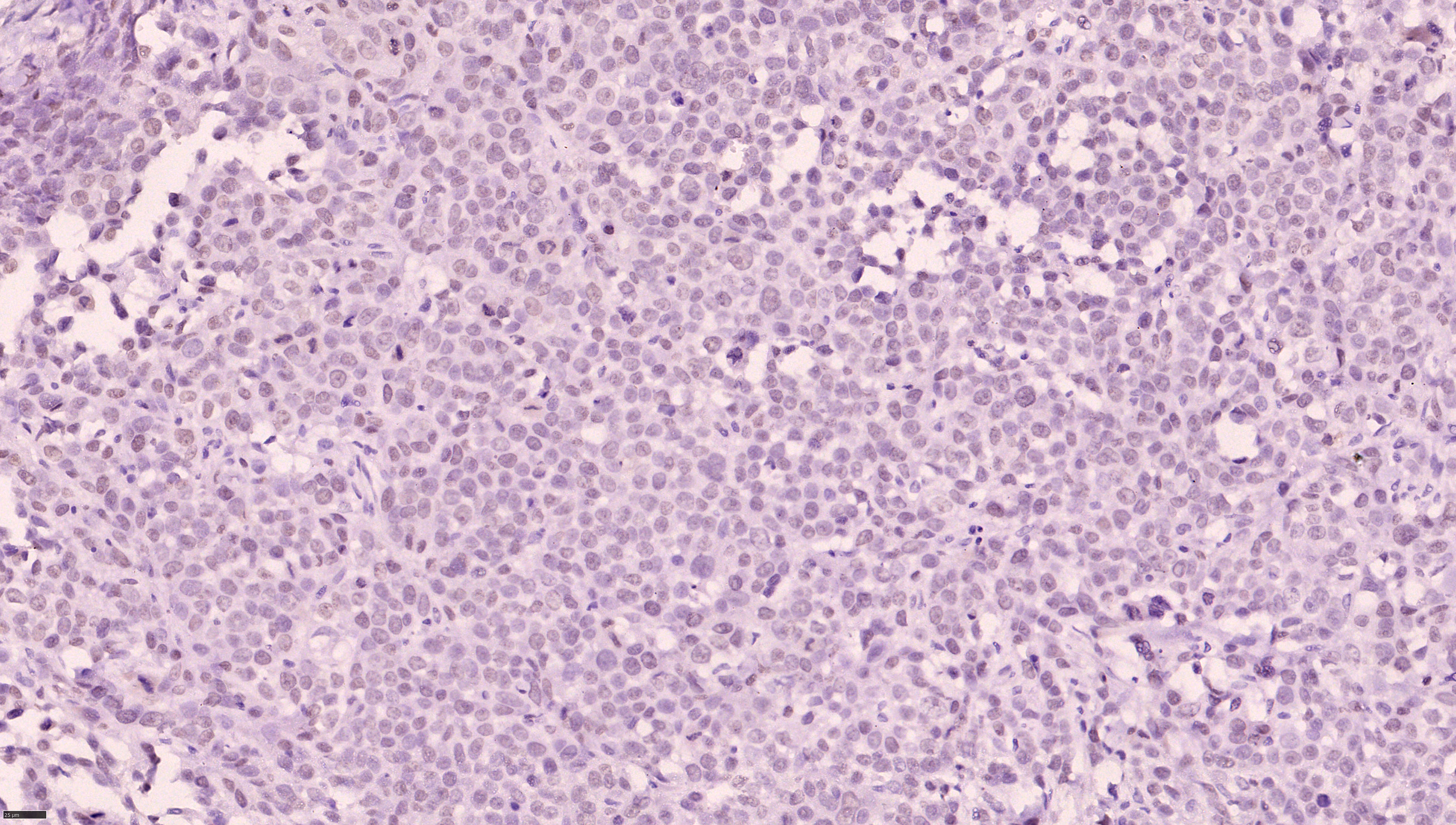

Supplement: Supplementary file 1 [file DataSheet1.zip › Figure 4 Excel/D/shLINC01572 20X.jpg]

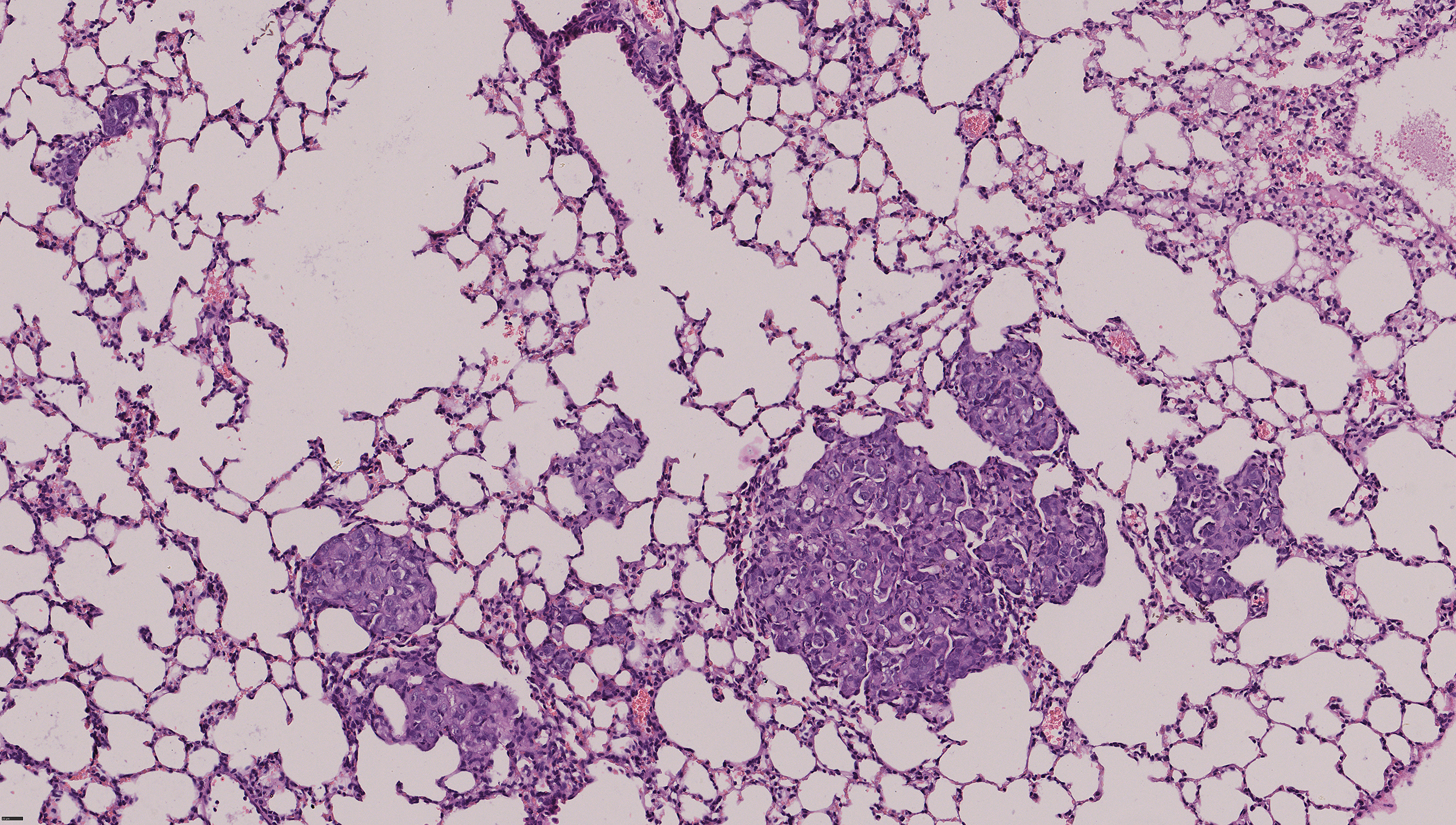

Supplement: Supplementary file 1 [file DataSheet1.zip › Figure 4 Excel/E/shCtrl+T 10X.jpg]

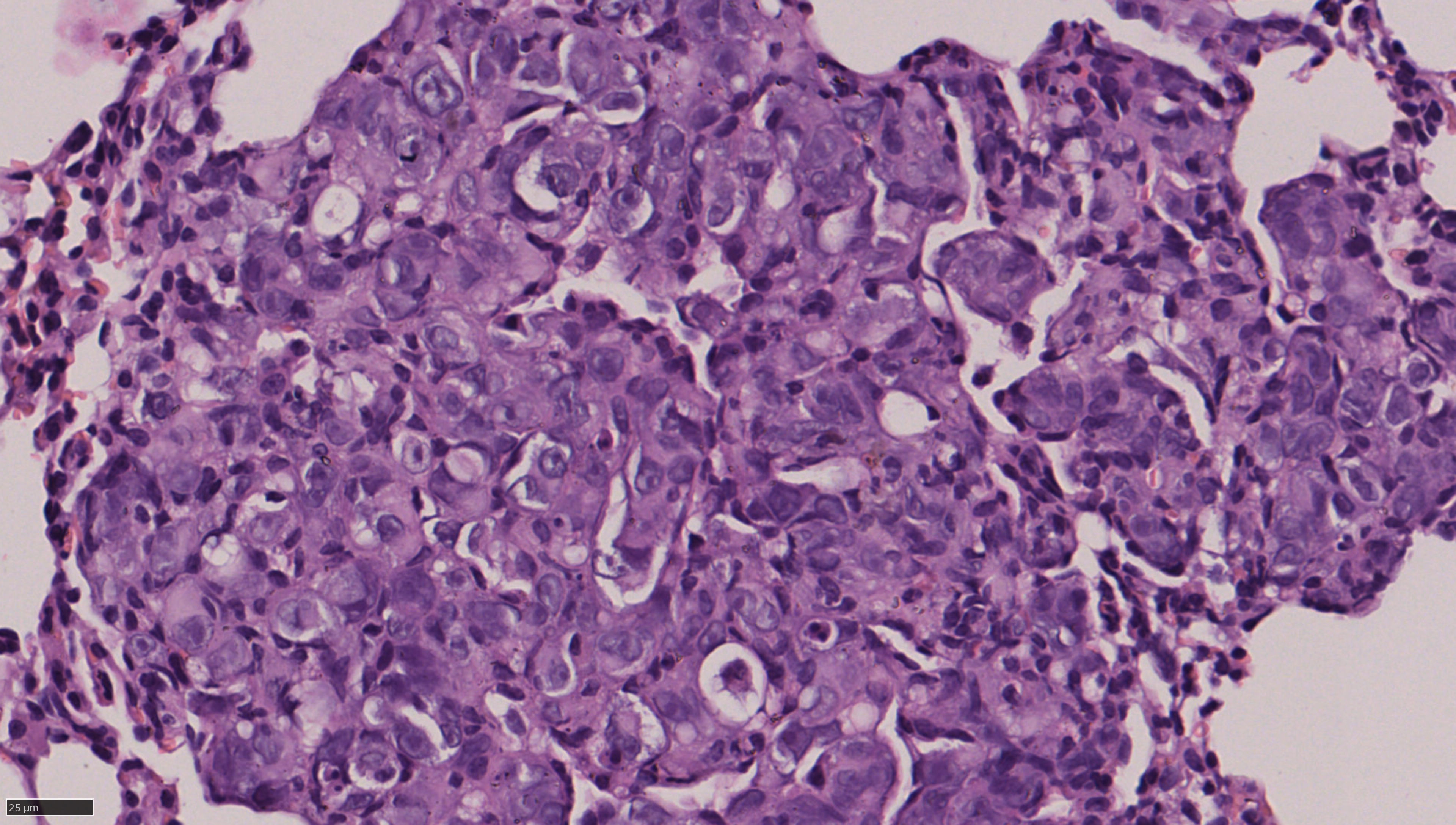

Supplement: Supplementary file 1 [file DataSheet1.zip › Figure 4 Excel/E/shCtrl+T 40X.jpg]

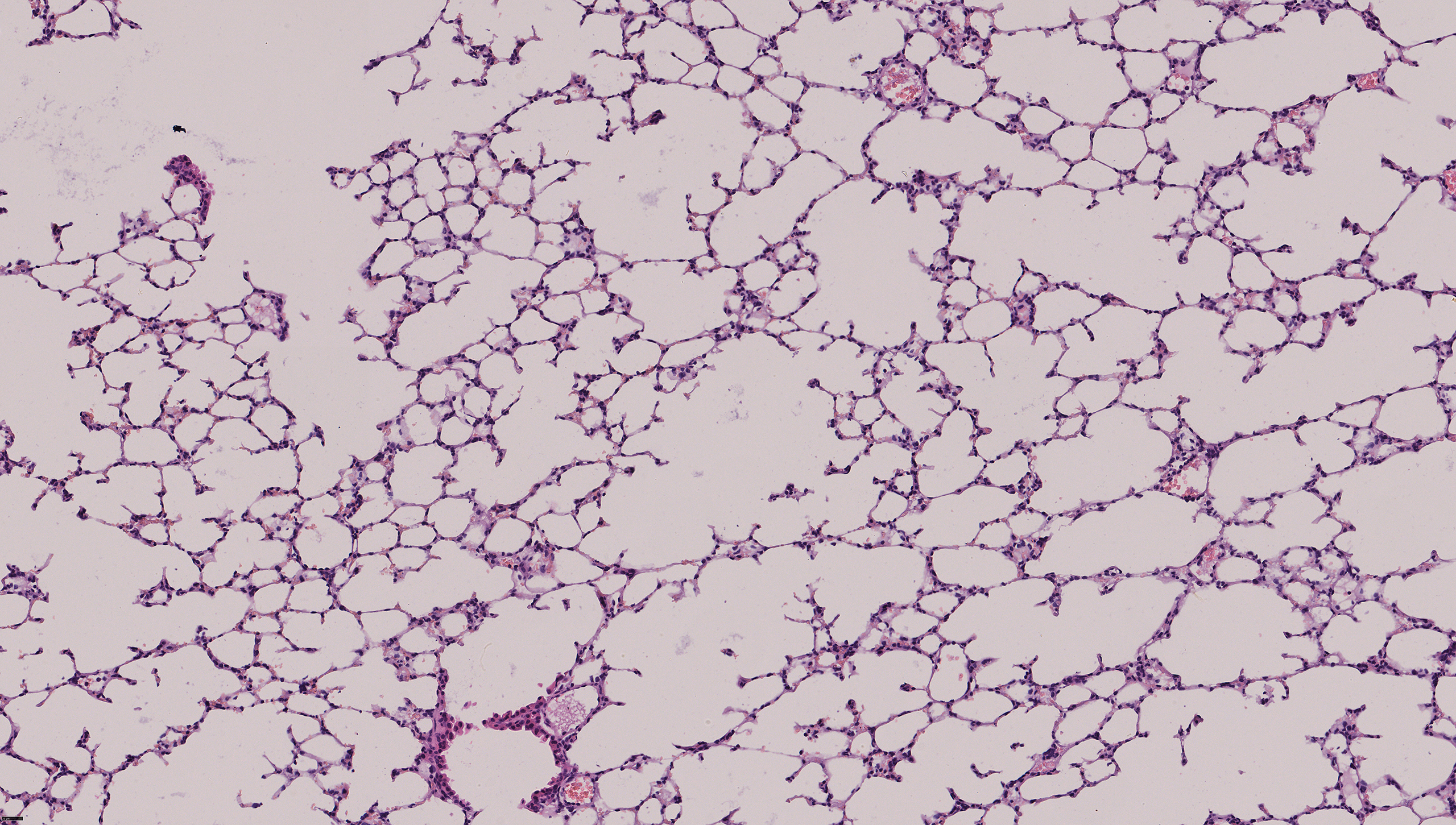

Supplement: Supplementary file 1 [file DataSheet1.zip › Figure 4 Excel/E/shLINC01572+T 10X.jpg]

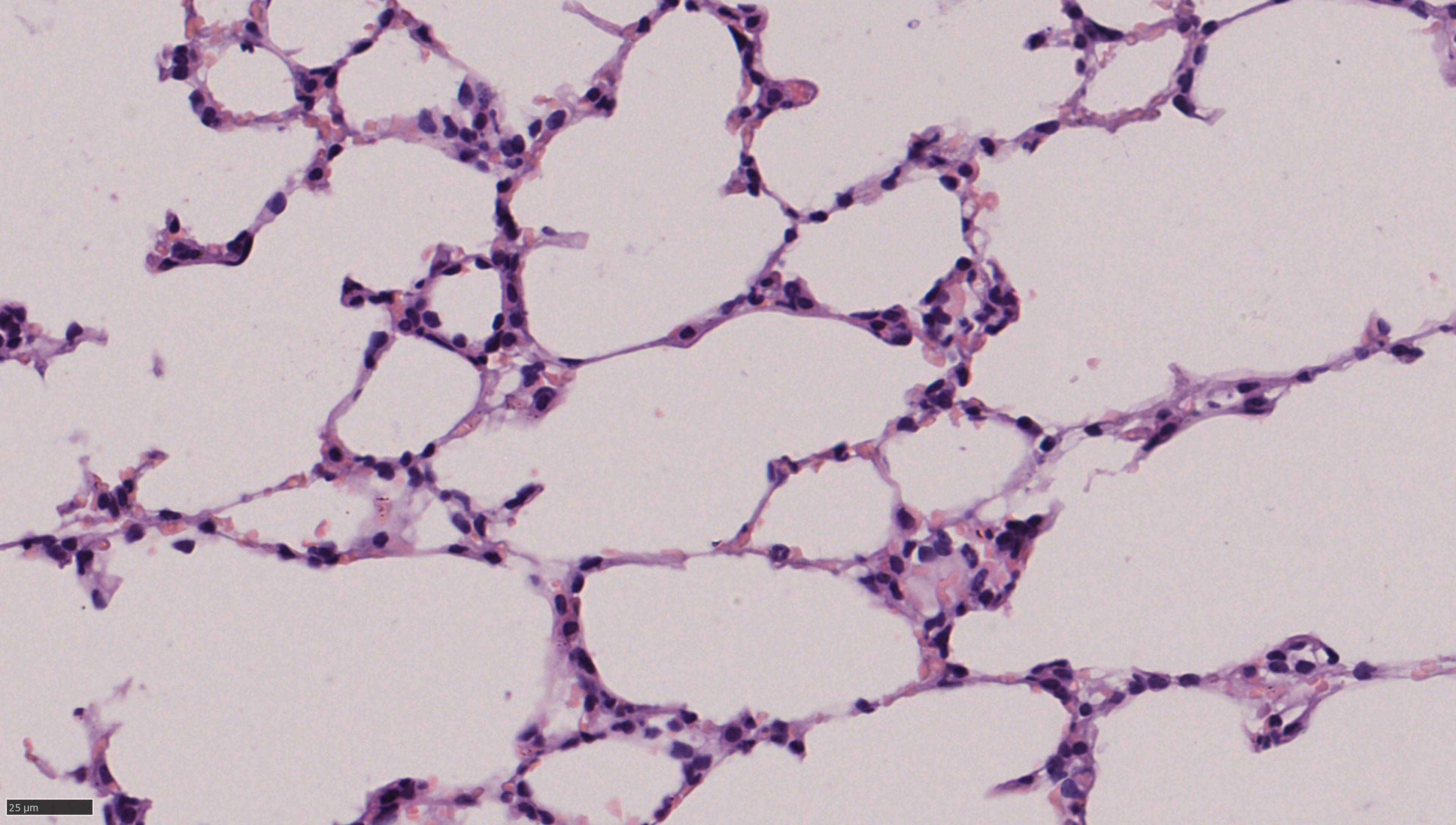

Supplement: Supplementary file 1 [file DataSheet1.zip › Figure 4 Excel/E/shLINC01572+T 40X.jpg]

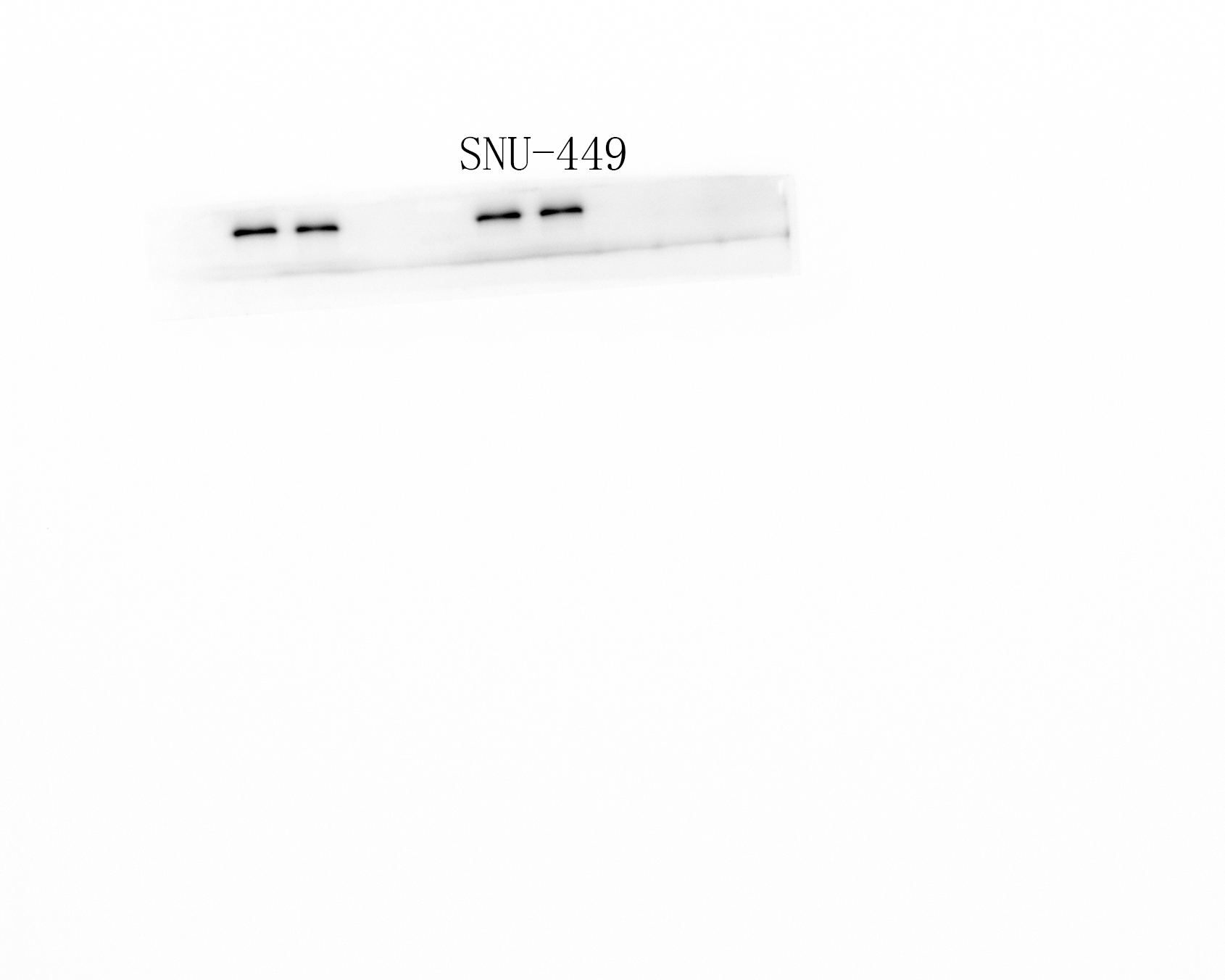

Supplement: Supplementary file 1 [file DataSheet1.zip › Figure 5 Excel/H/AGO2 1.jpg]

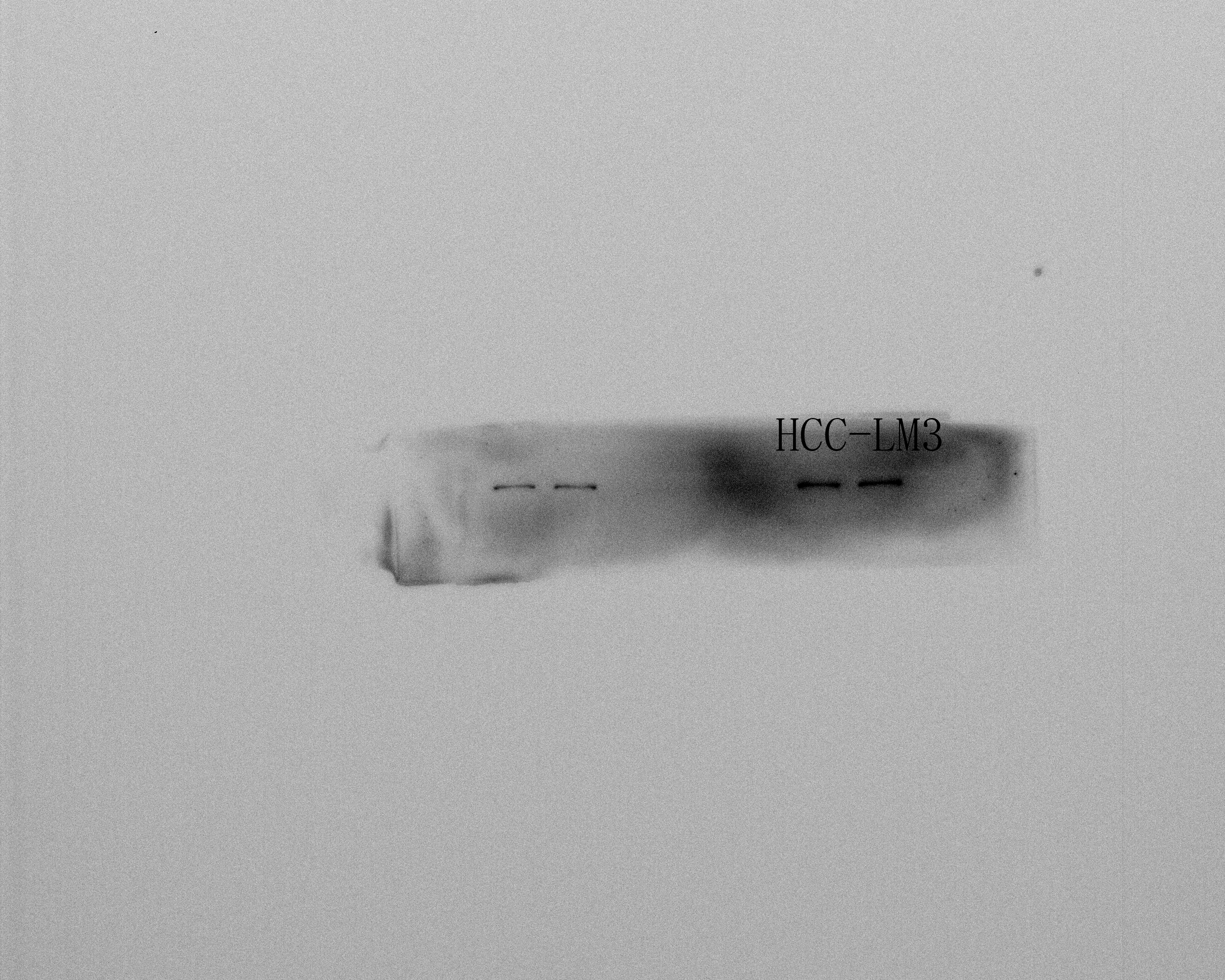

Supplement: Supplementary file 1 [file DataSheet1.zip › Figure 5 Excel/I/AGO2 2.jpg]

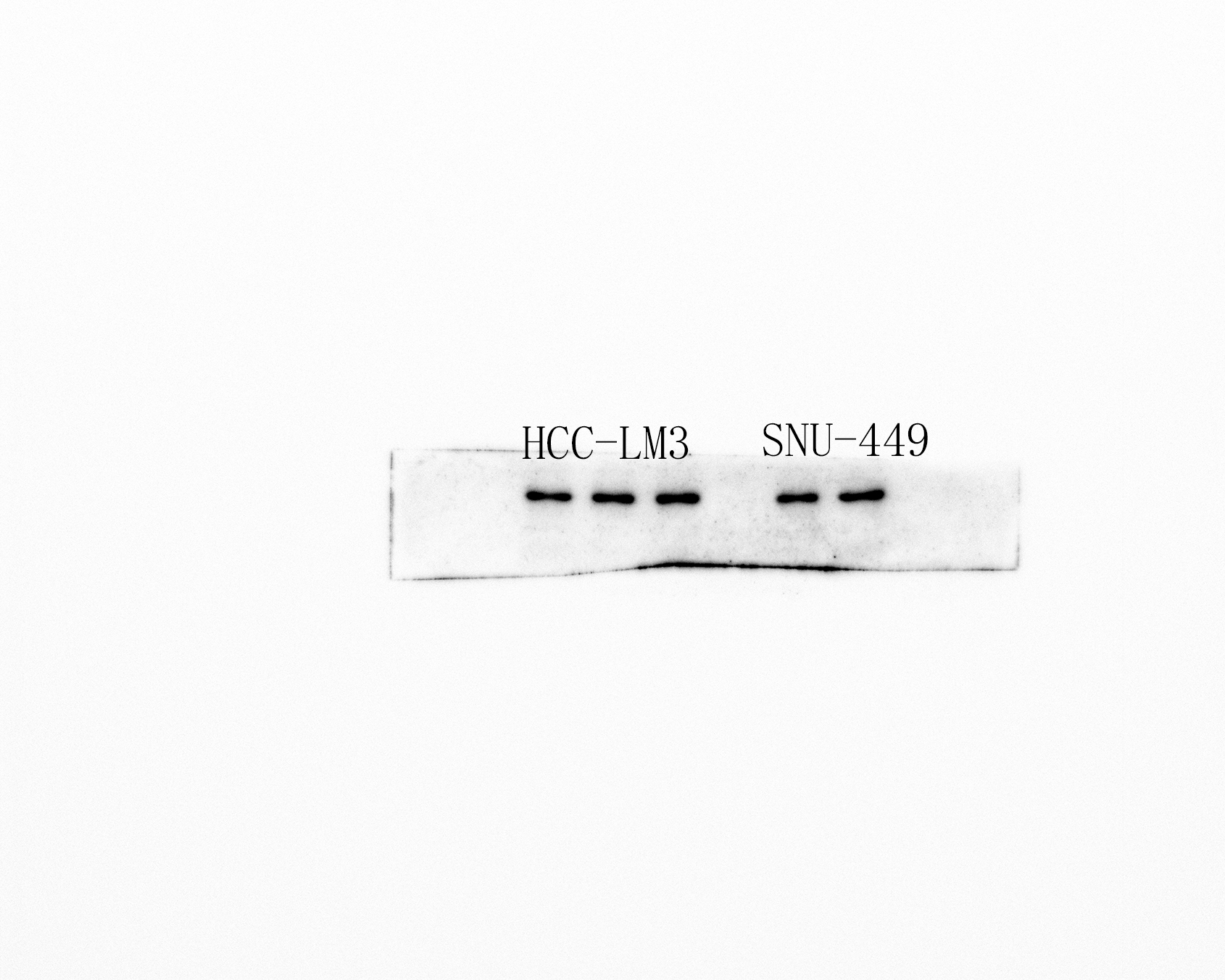

Supplement: Supplementary file 1 [file DataSheet1.zip › Figure 6 Excel/a--actin 3.jpg]

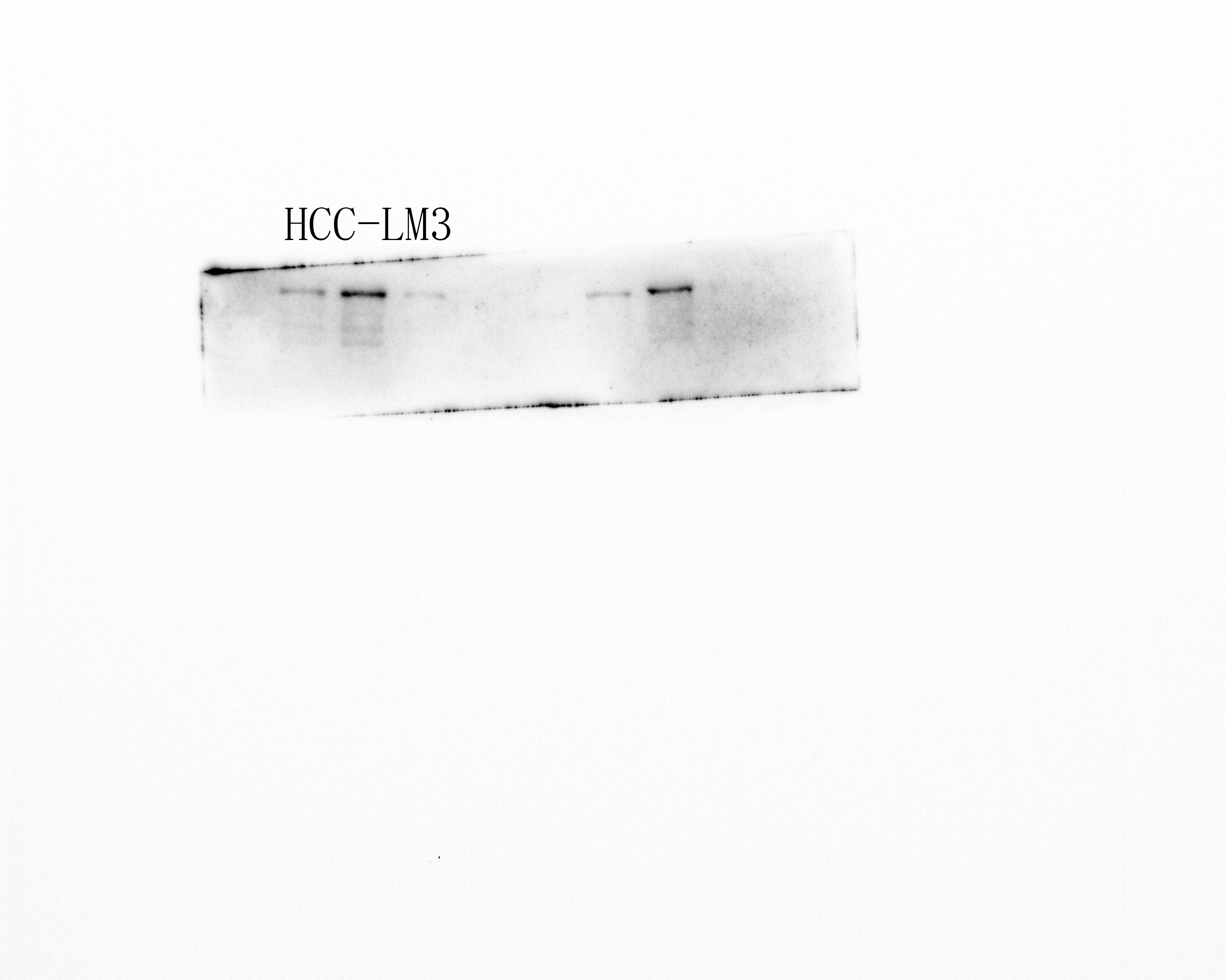

Supplement: Supplementary file 1 [file DataSheet1.zip › Figure 6 Excel/PFKFB4 2.jpg]

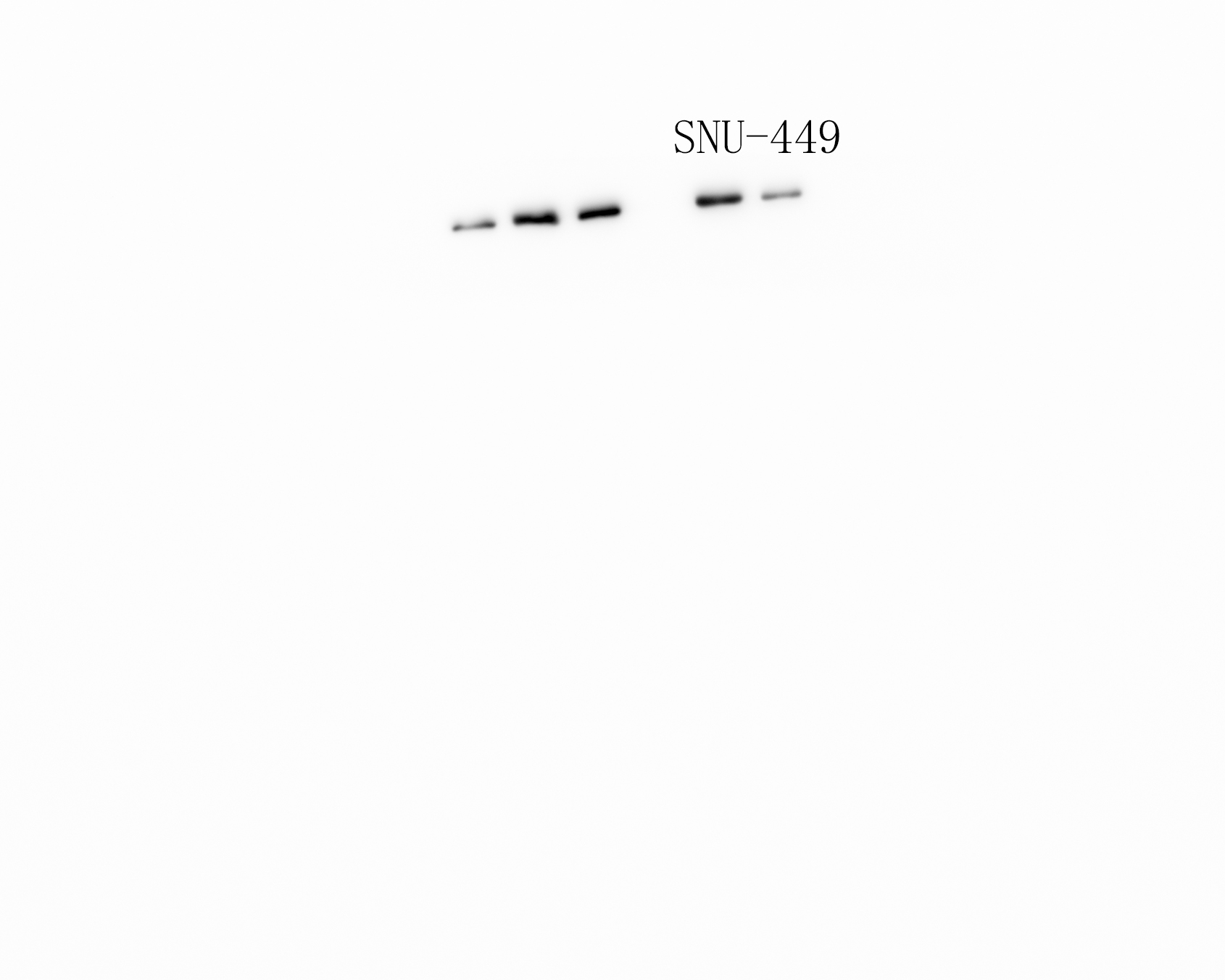

Supplement: Supplementary file 1 [file DataSheet1.zip › Figure 6 Excel/PFKFB4 1.jpg]

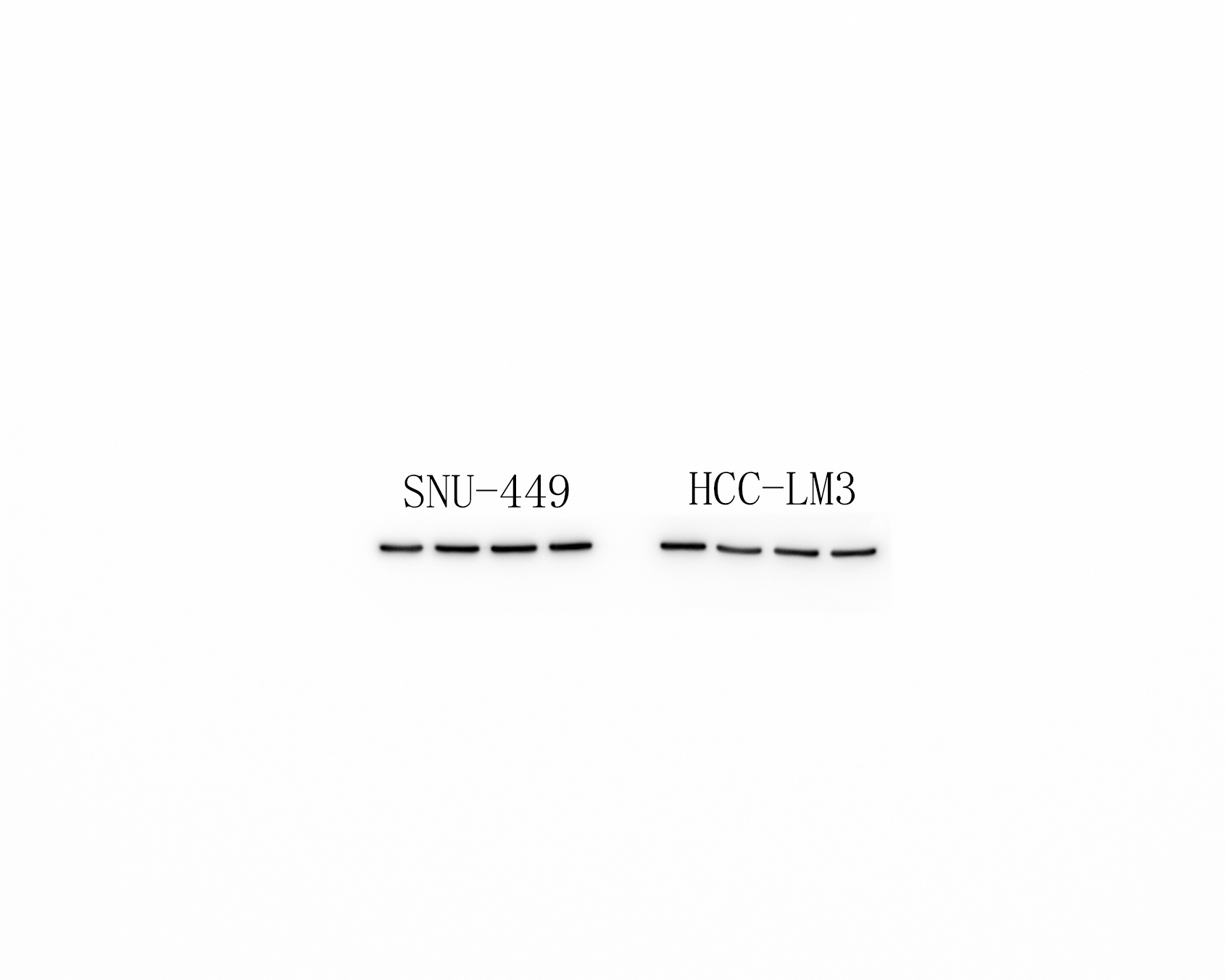

Supplement: Supplementary file 1 [file DataSheet1.zip › Figure 7 Excel/WB/a--actin.jpg]

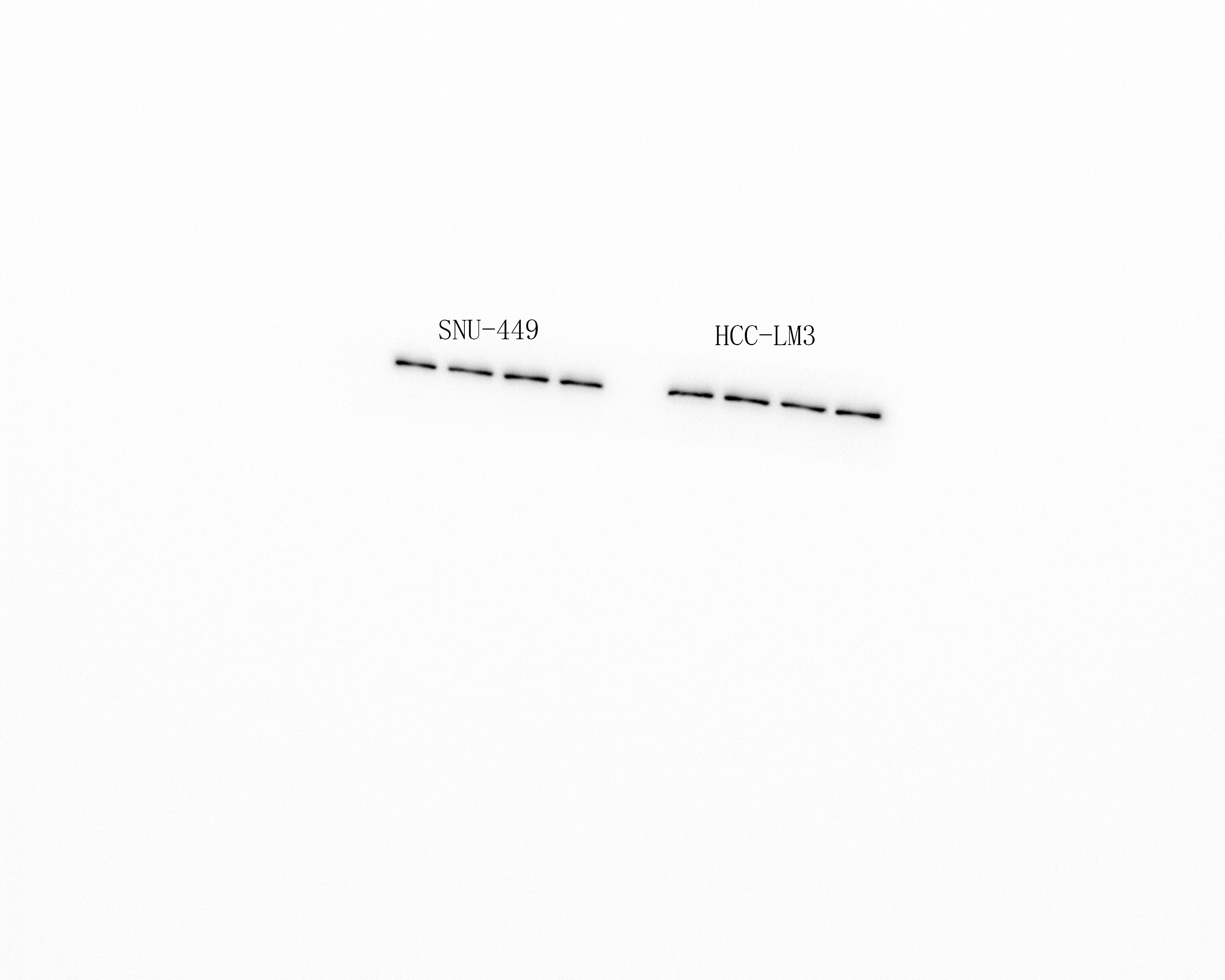

Supplement: Supplementary file 1 [file DataSheet1.zip › Figure 7 Excel/WB/AKT.jpg]

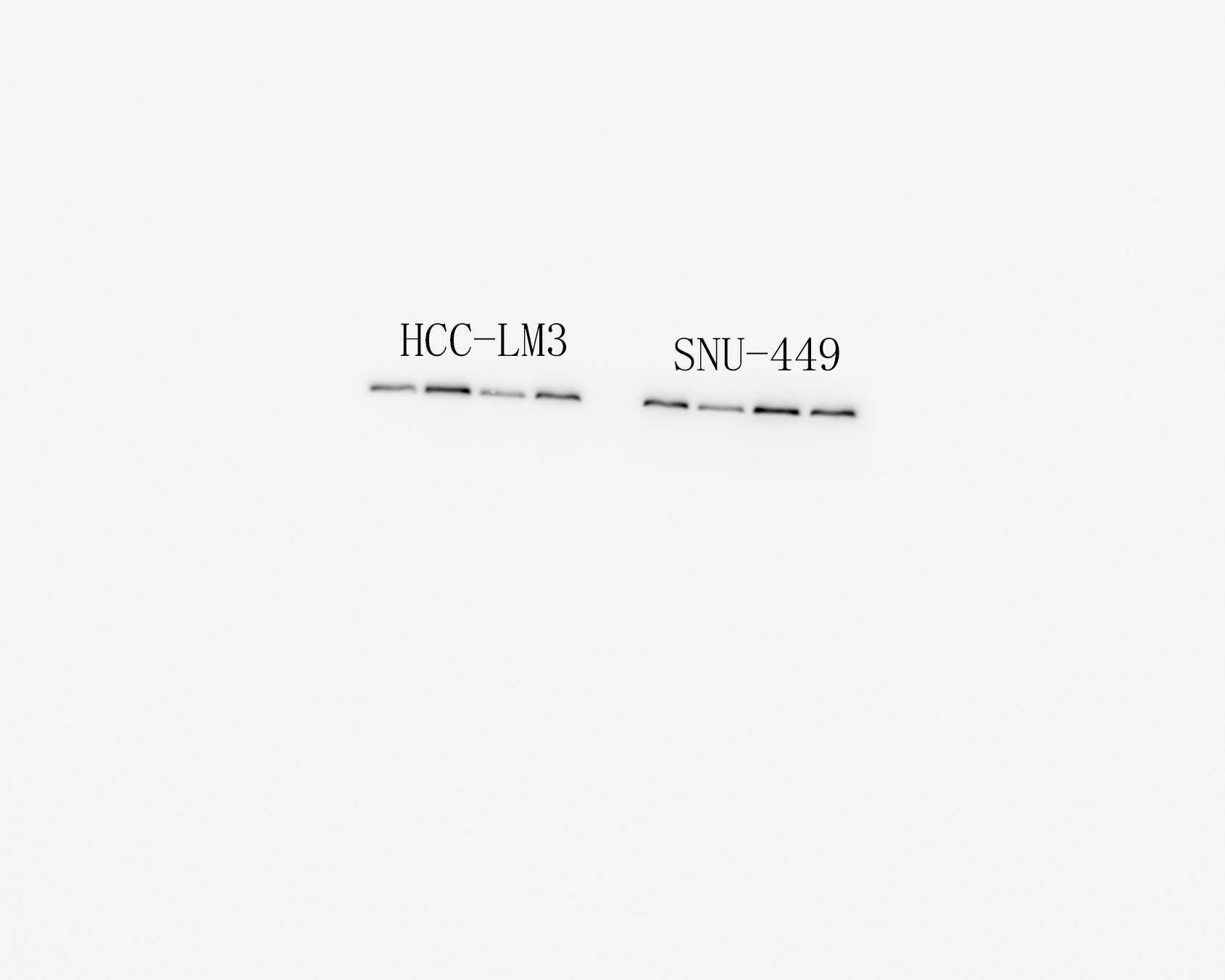

Supplement: Supplementary file 1 [file DataSheet1.zip › Figure 7 Excel/WB/P-AKT.jpg]

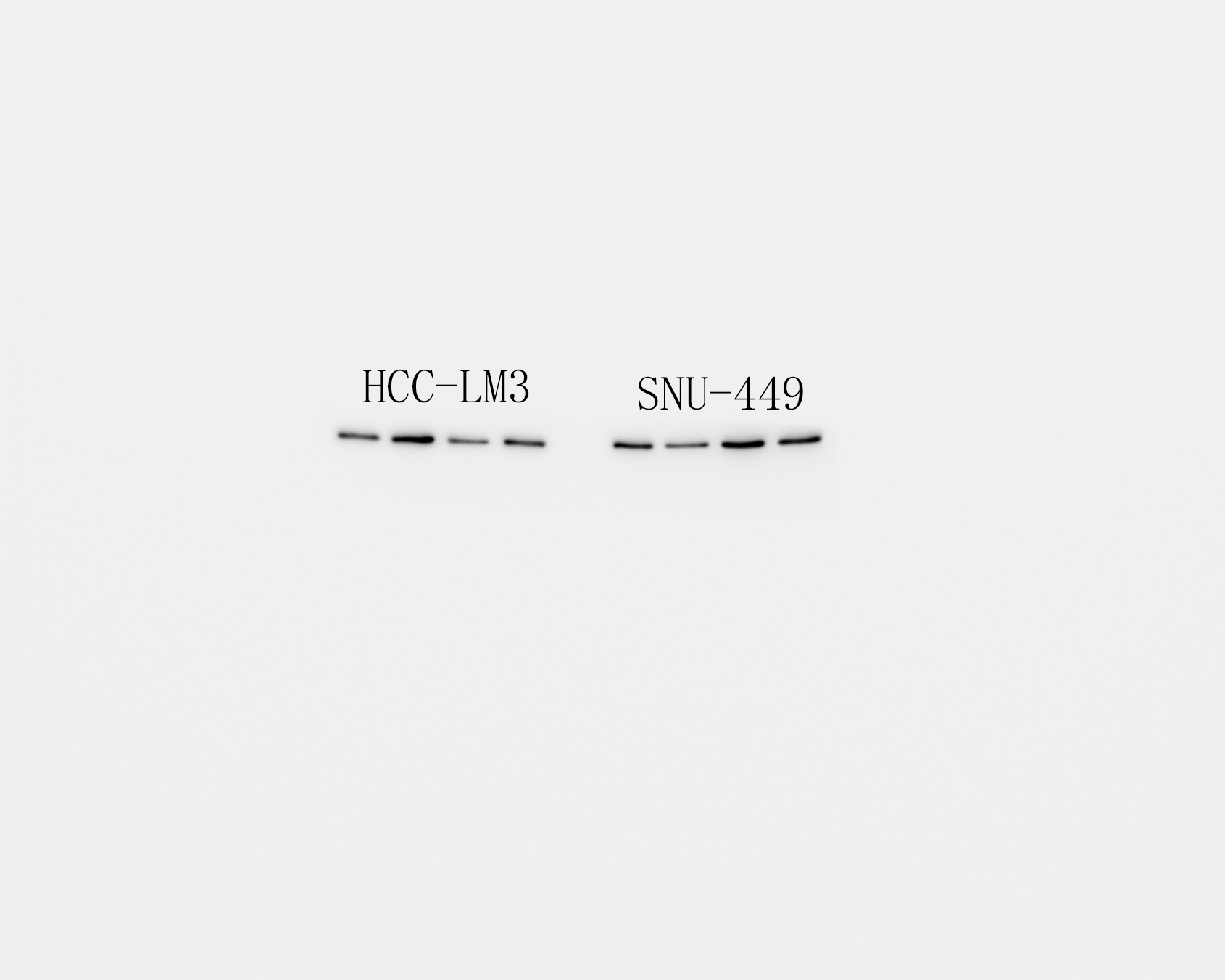

Supplement: Supplementary file 1 [file DataSheet1.zip › Figure 7 Excel/WB/PFKFB4.jpg]

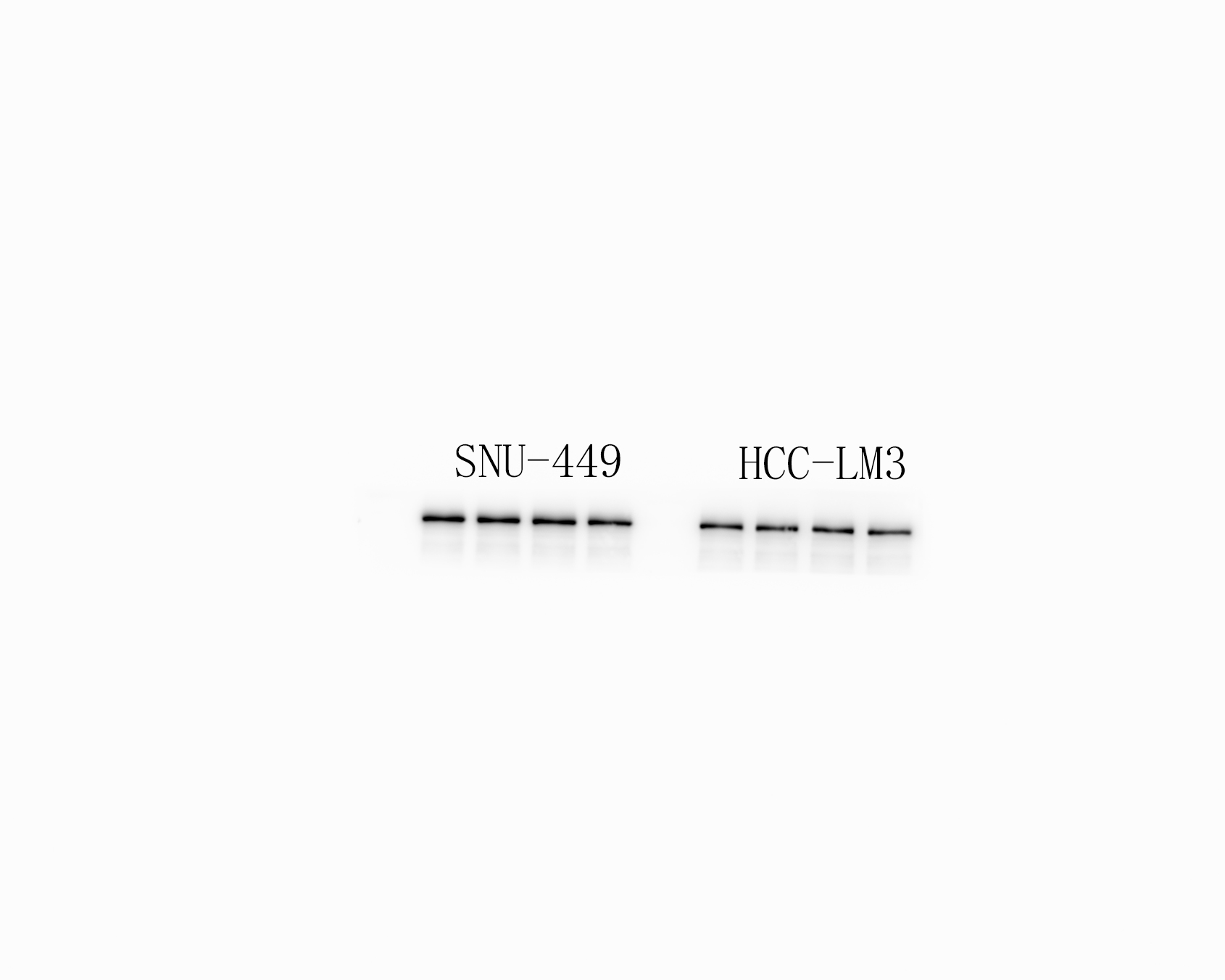

Supplement: Supplementary file 1 [file DataSheet1.zip › Figure 7 Excel/WB/PI3K.jpg]

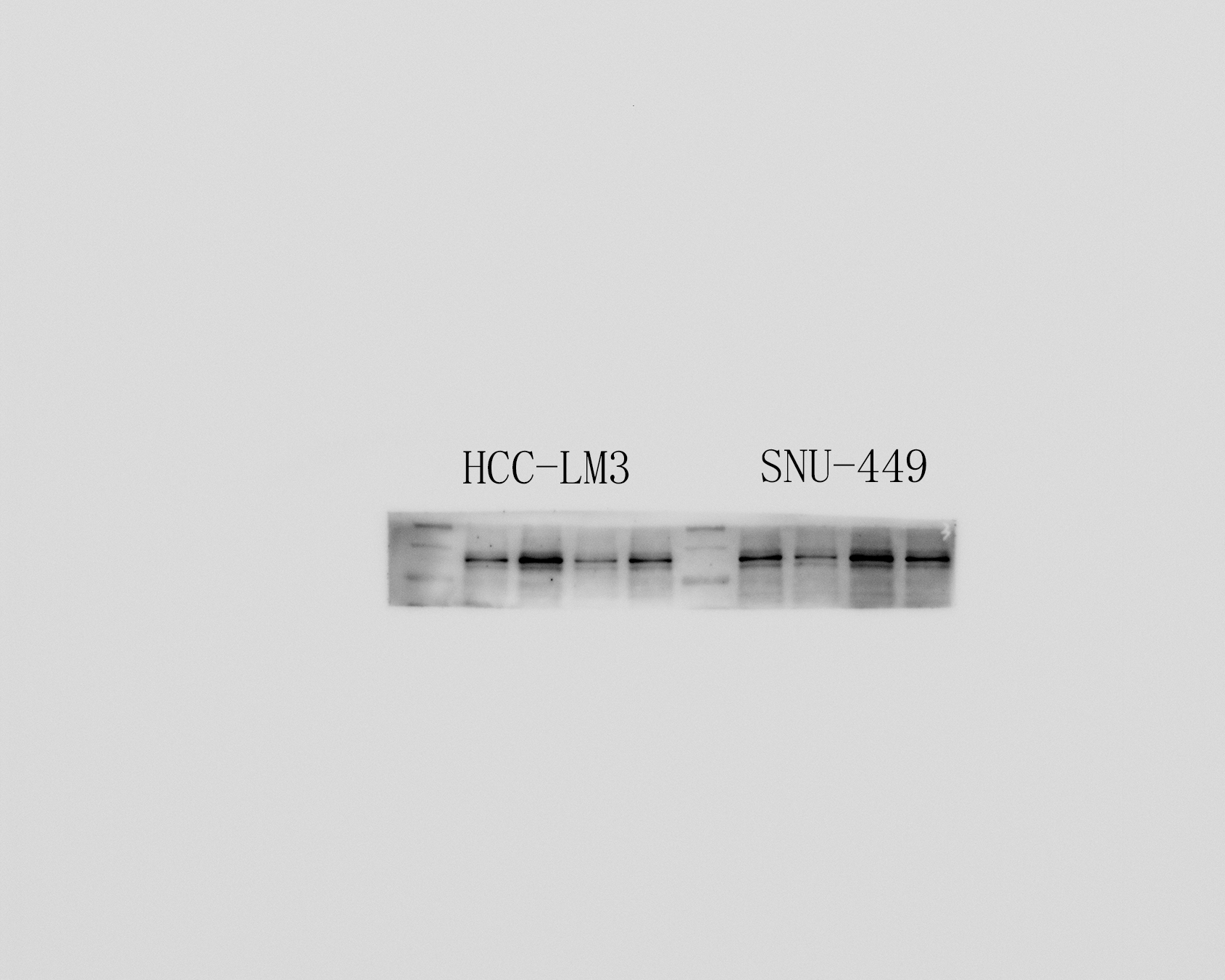

Supplement: Supplementary file 1 [file DataSheet1.zip › Figure 7 Excel/WB/P-PI3K.jpg]

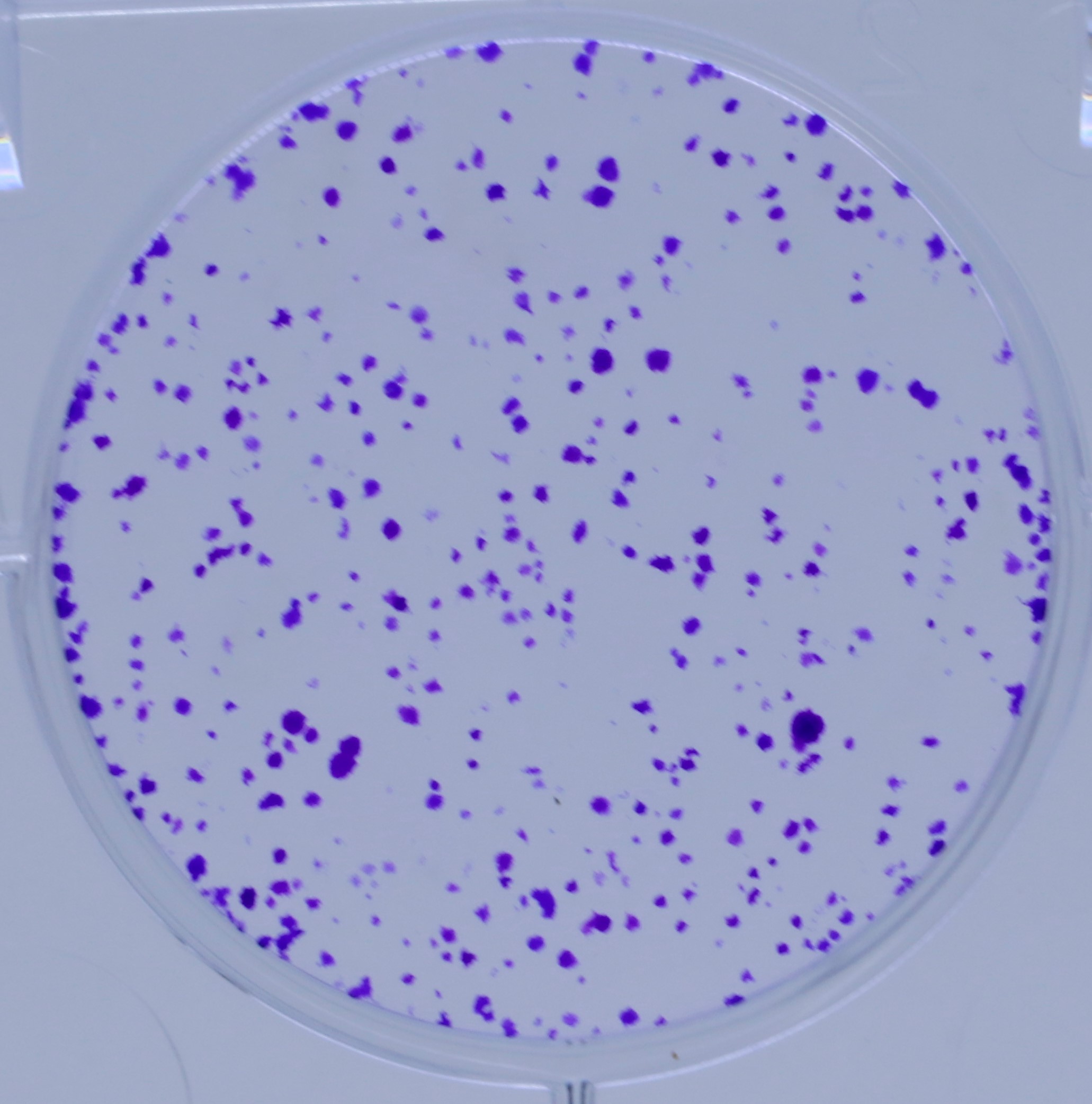

Supplement: Supplementary file 1 [file DataSheet1.zip › Figure 8 Excel/C/NC.JPG]

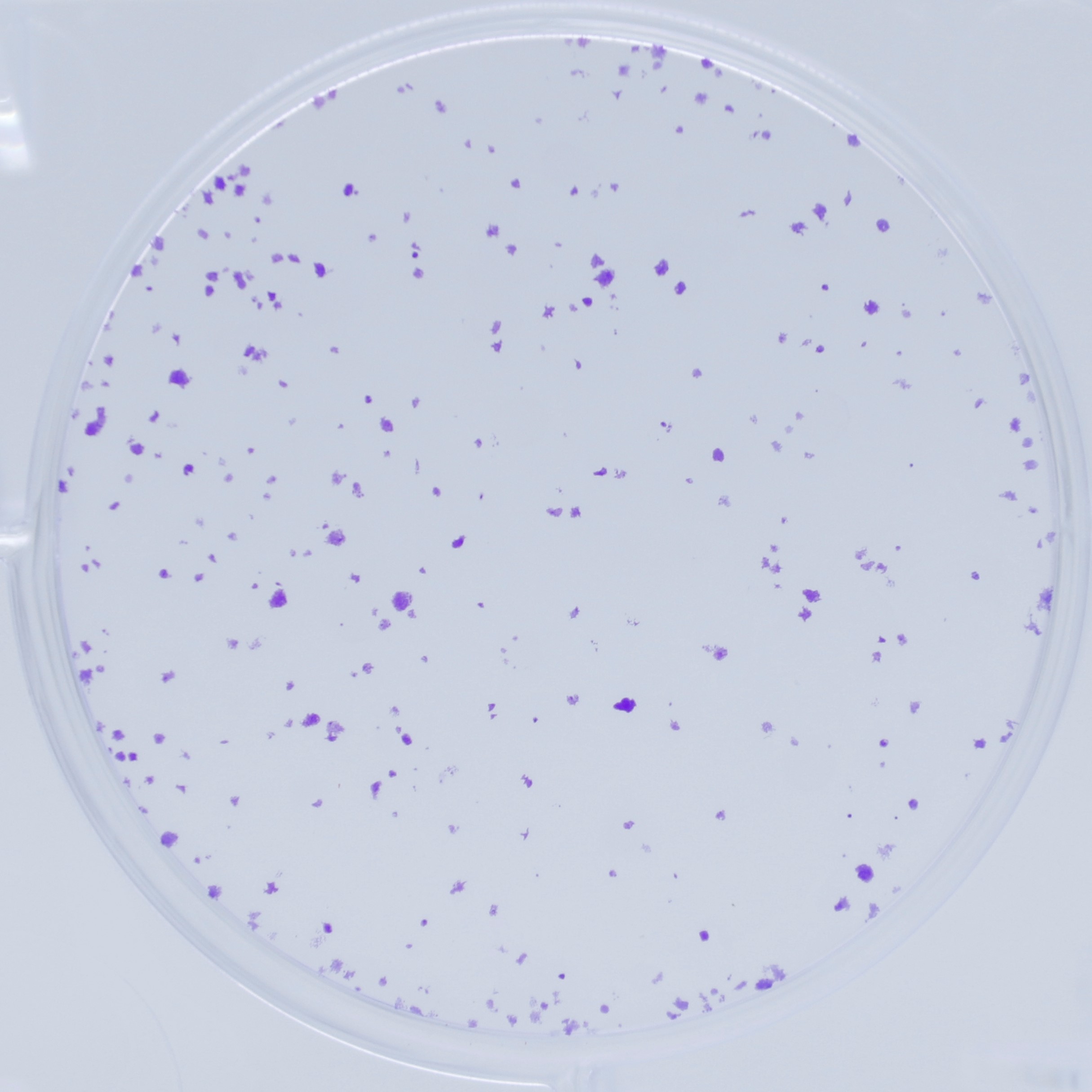

Supplement: Supplementary file 1 [file DataSheet1.zip › Figure 8 Excel/C/shLINC01572.JPG]

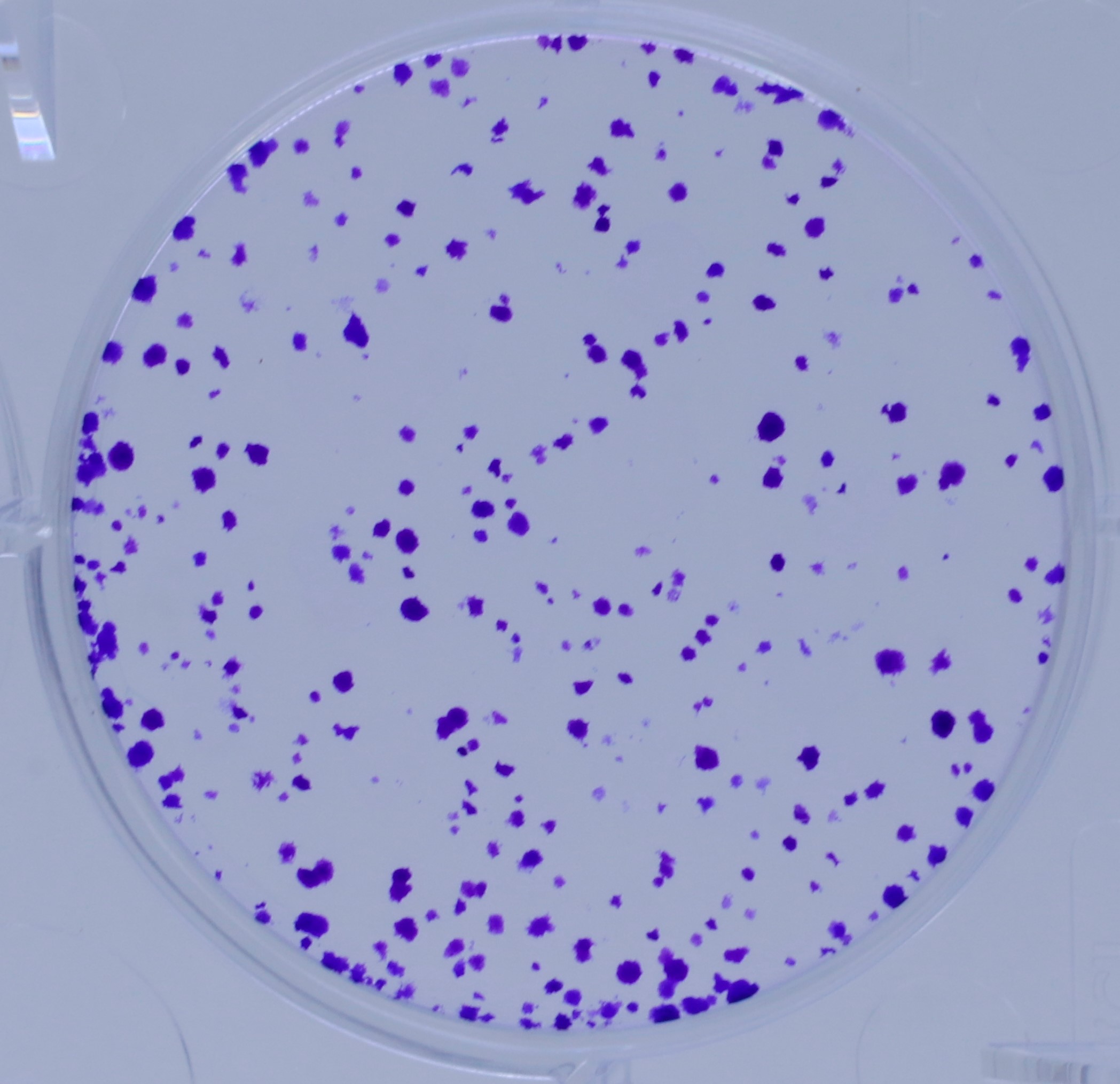

Supplement: Supplementary file 1 [file DataSheet1.zip › Figure 8 Excel/C/shLINC01572+PFKFB4.JPG]

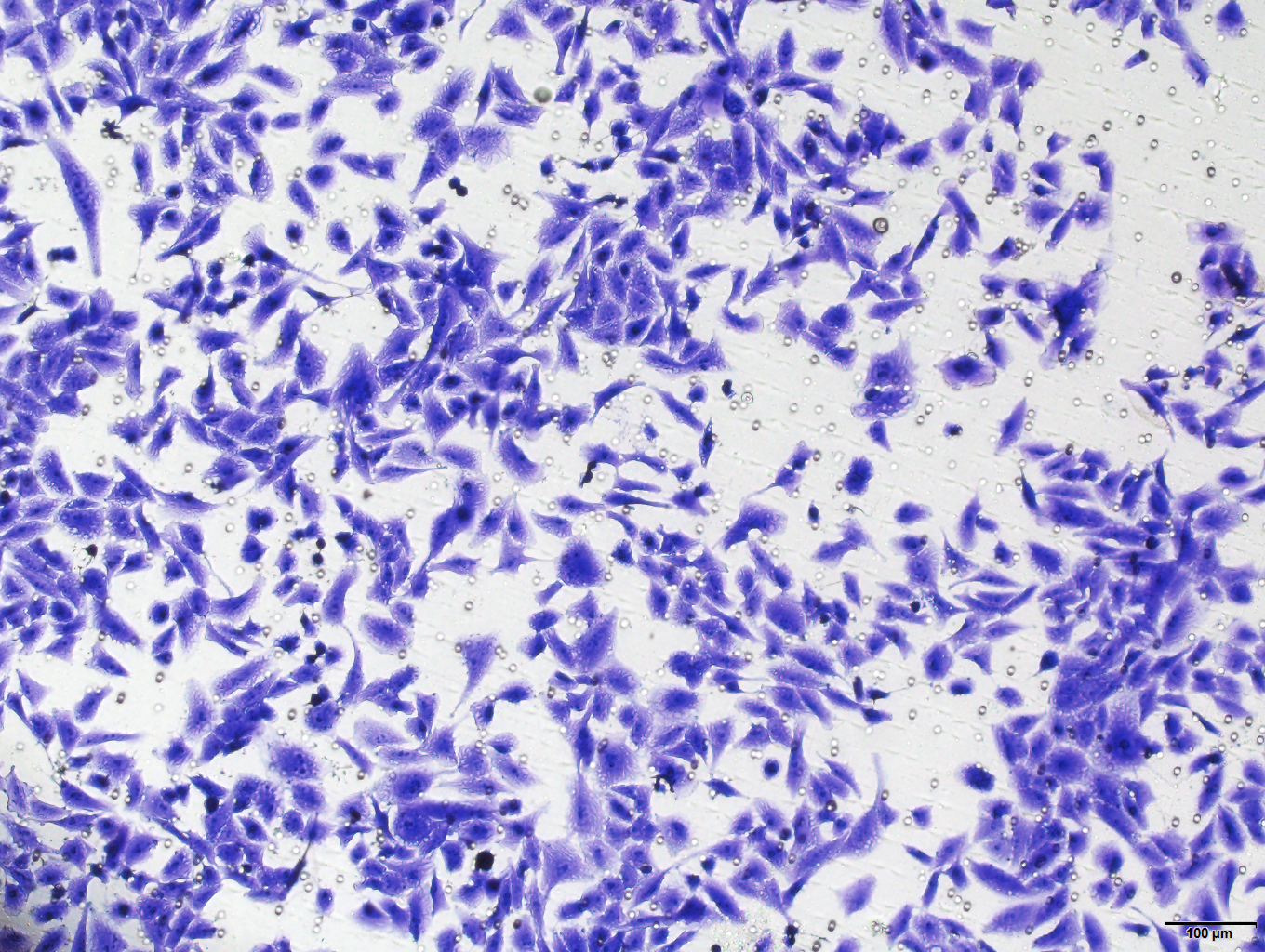

Supplement: Supplementary file 1 [file DataSheet1.zip › Figure 8 Excel/D/▌¿-▌/NC.tif]

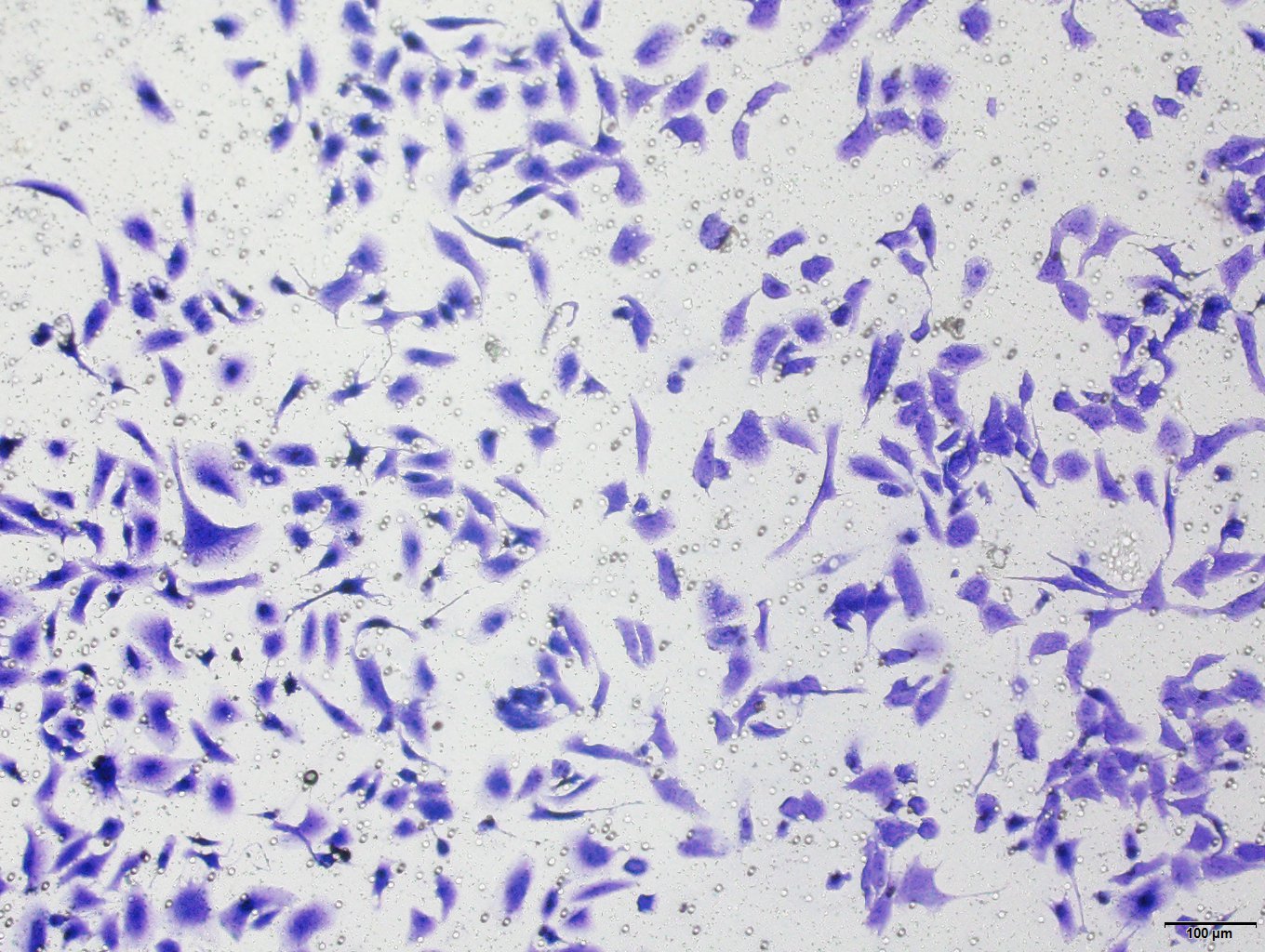

Supplement: Supplementary file 1 [file DataSheet1.zip › Figure 8 Excel/D/▌¿-▌/shLINC01572#1.jpg]

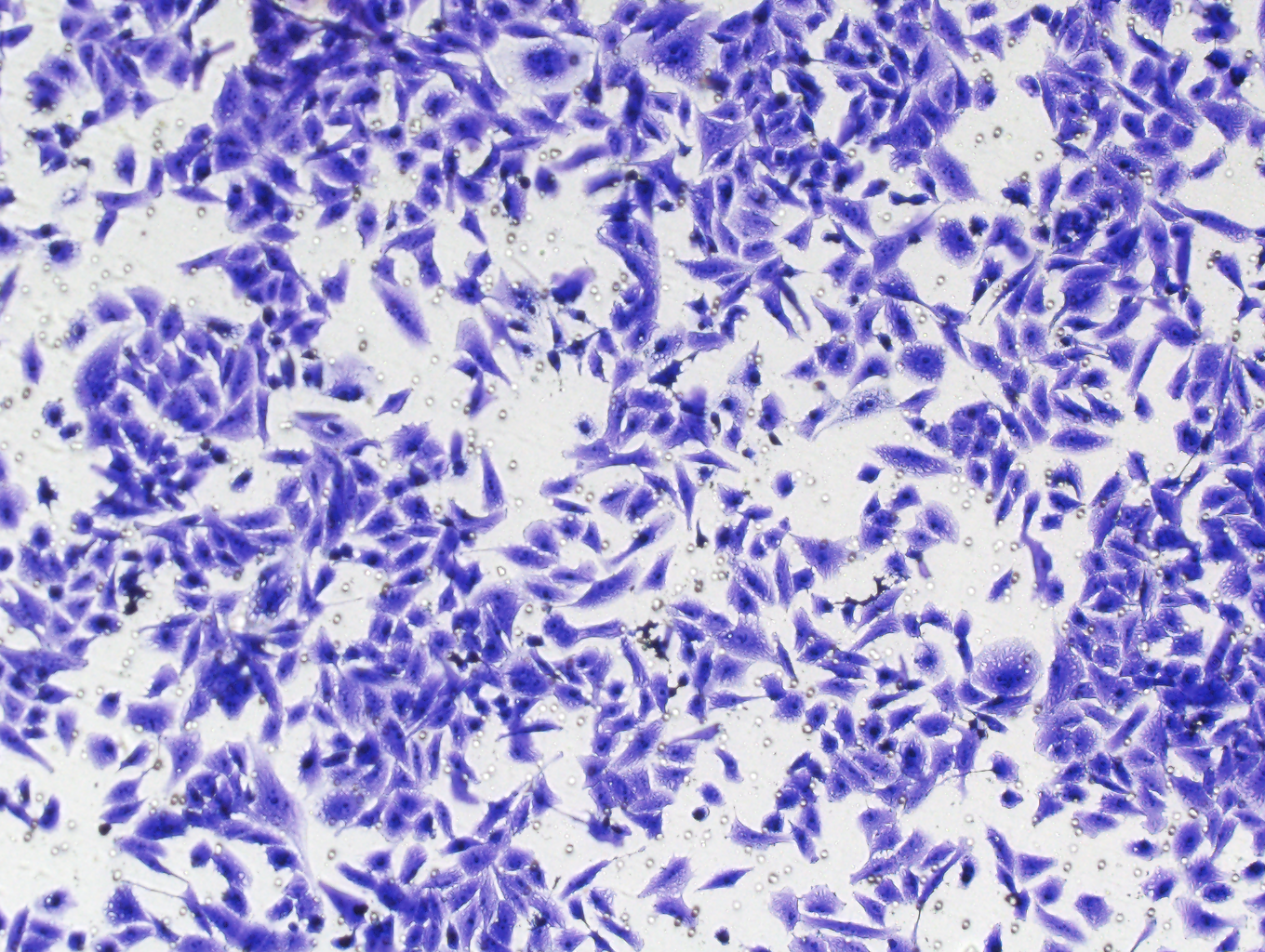

Supplement: Supplementary file 1 [file DataSheet1.zip › Figure 8 Excel/D/▌¿-▌/shLINC01572#1+PFKFB4.tif]

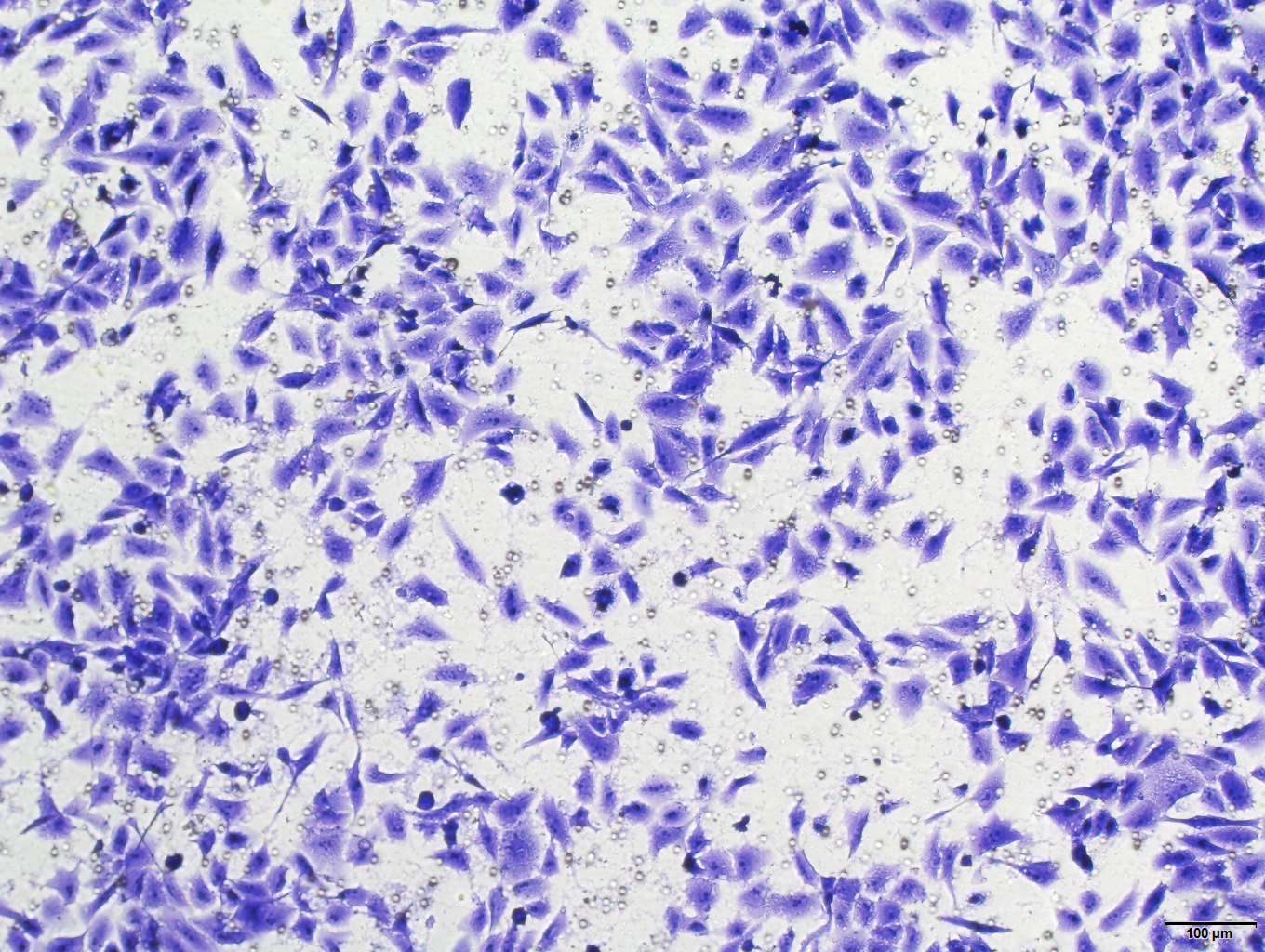

Supplement: Supplementary file 1 [file DataSheet1.zip › Figure 8 Excel/D/▌+-«/NC.tif]

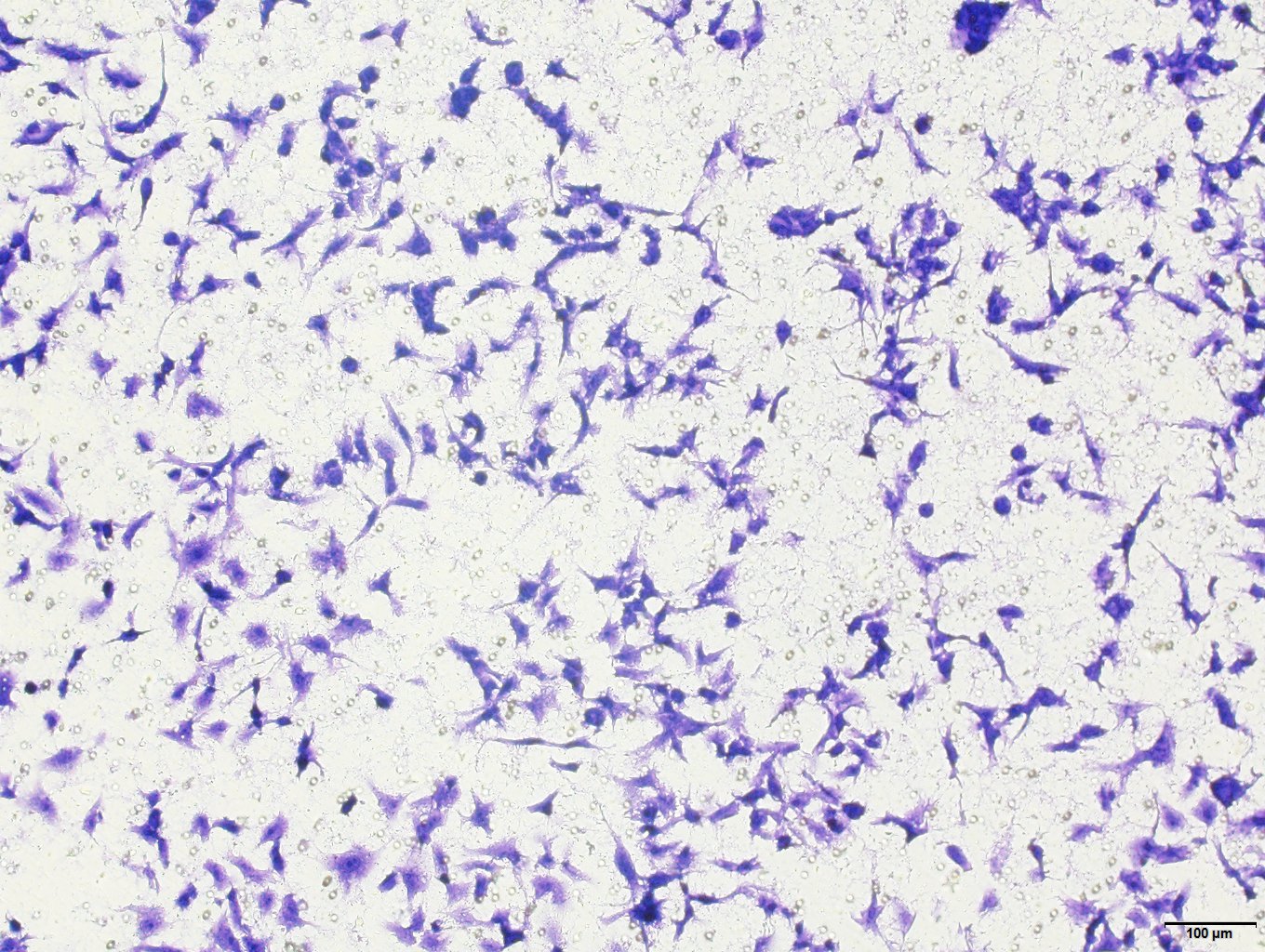

Supplement: Supplementary file 1 [file DataSheet1.zip › Figure 8 Excel/D/▌+-«/shLINC01572#1.jpg]

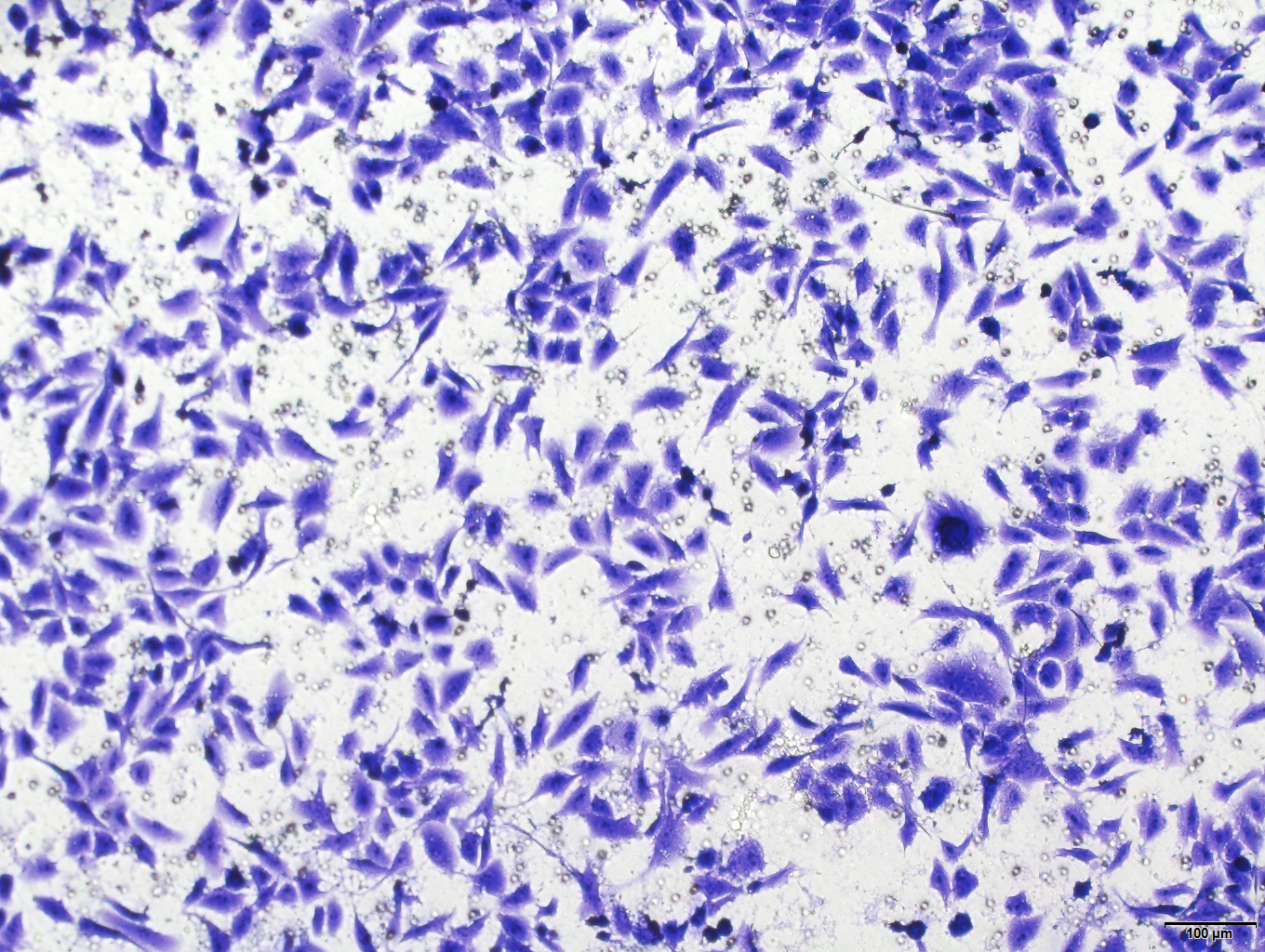

Supplement: Supplementary file 1 [file DataSheet1.zip › Figure 8 Excel/D/▌+-«/shLINC01572#1+PFKFB4.tif]

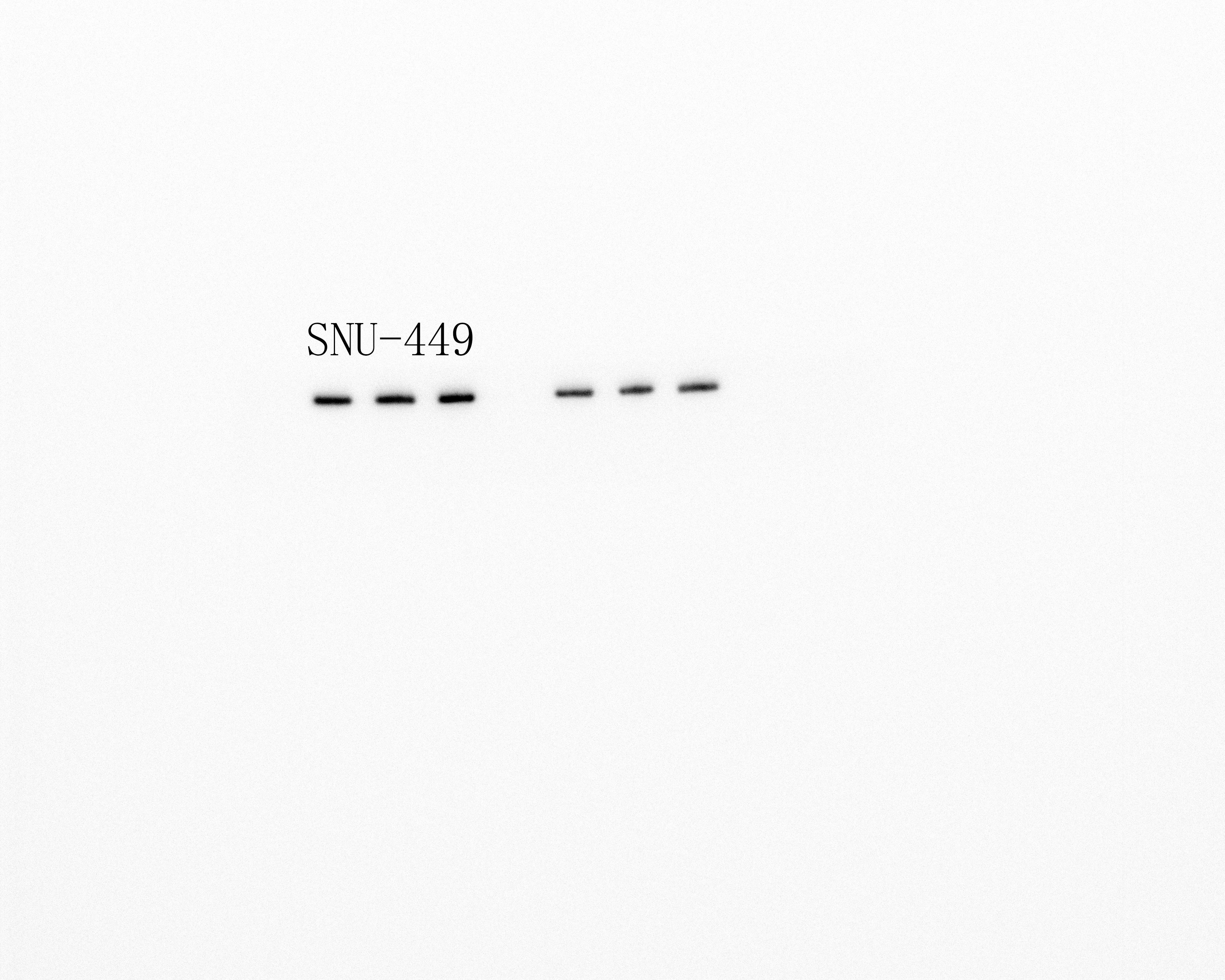

Supplement: Supplementary file 1 [file DataSheet1.zip › Figure 8 Excel/E WB/a--actin 3.jpg]

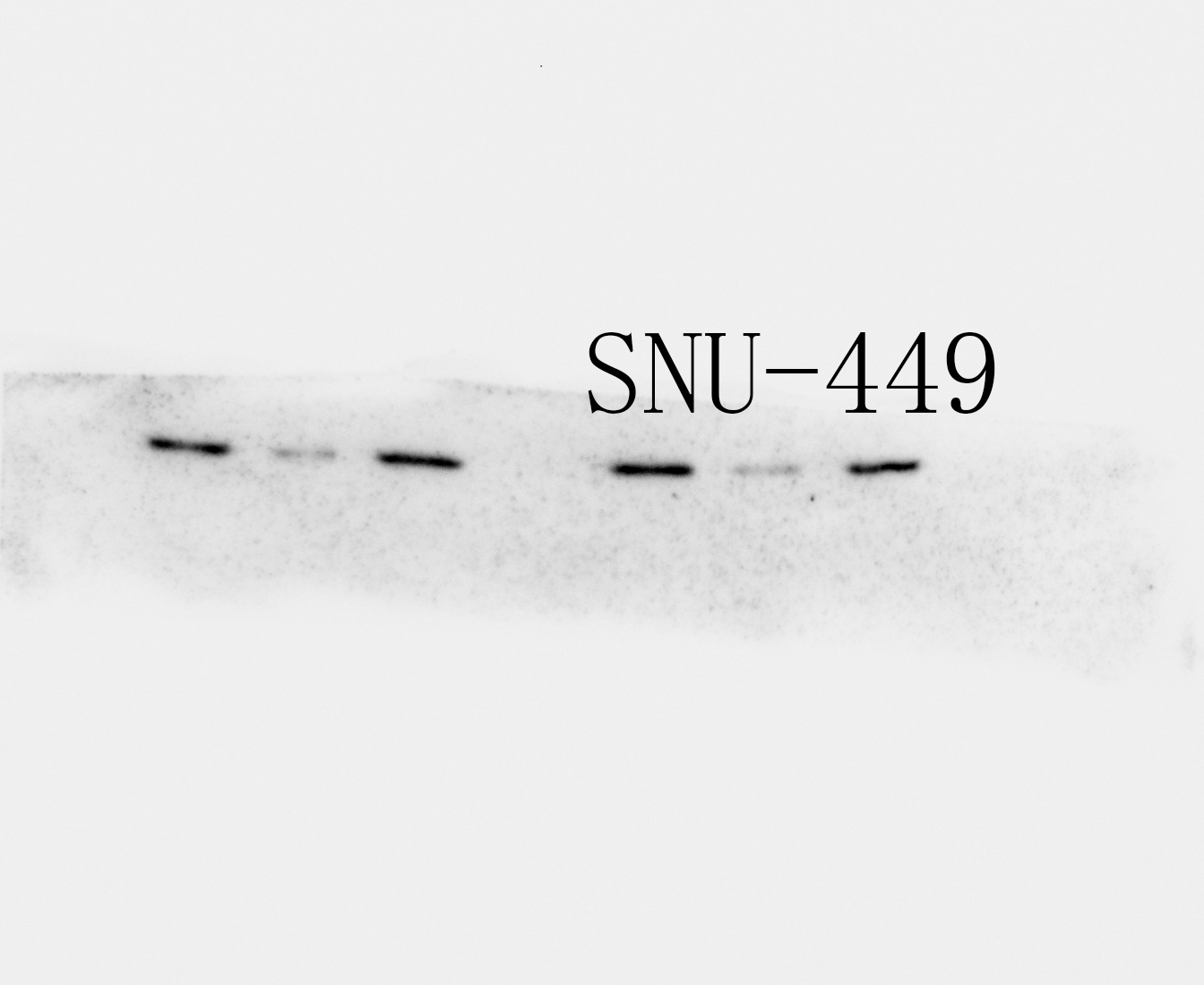

Supplement: Supplementary file 1 [file DataSheet1.zip › Figure 8 Excel/E WB/a--catenin 3.jpg]

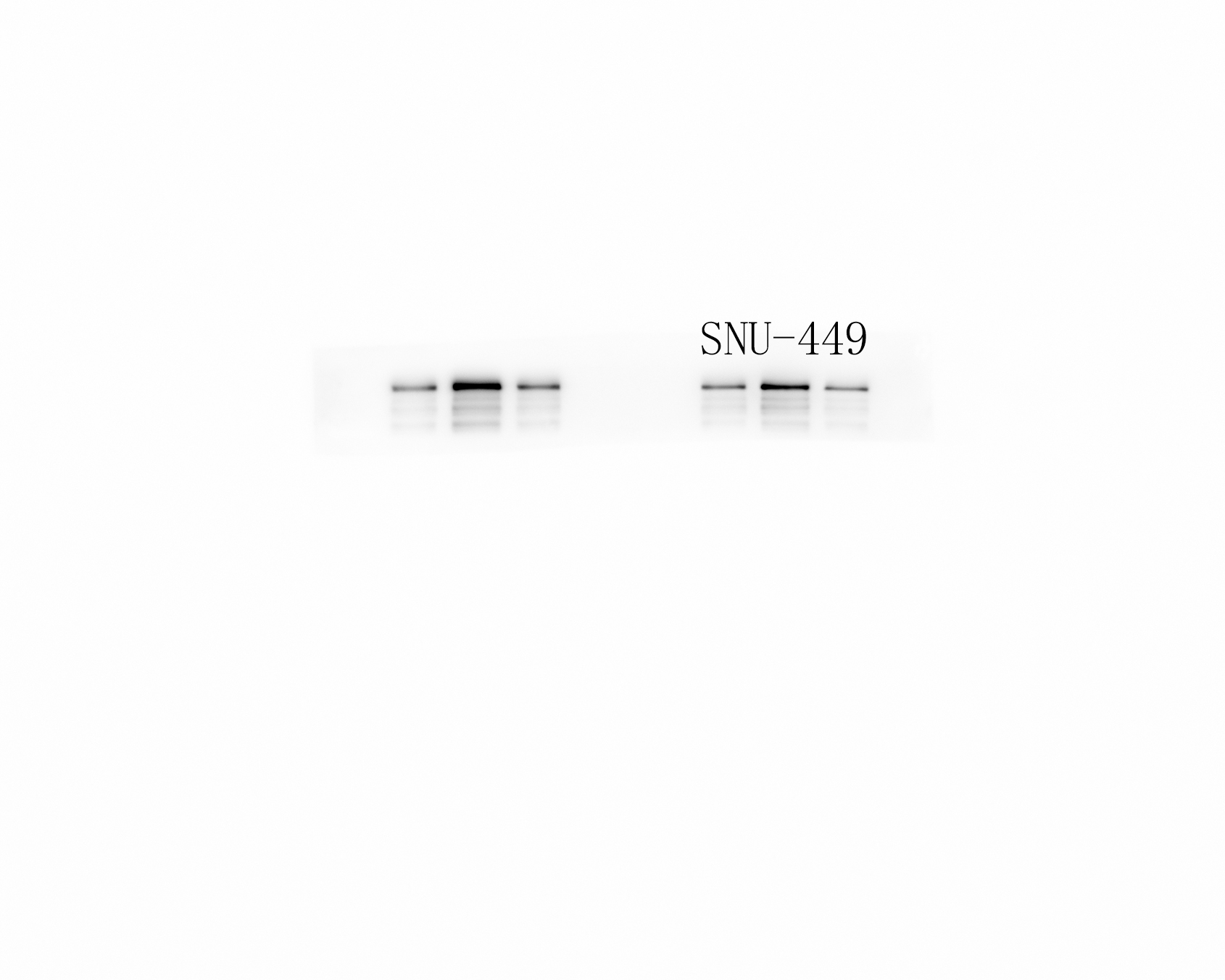

Supplement: Supplementary file 1 [file DataSheet1.zip › Figure 8 Excel/E WB/E-cadherin 3.jpg]

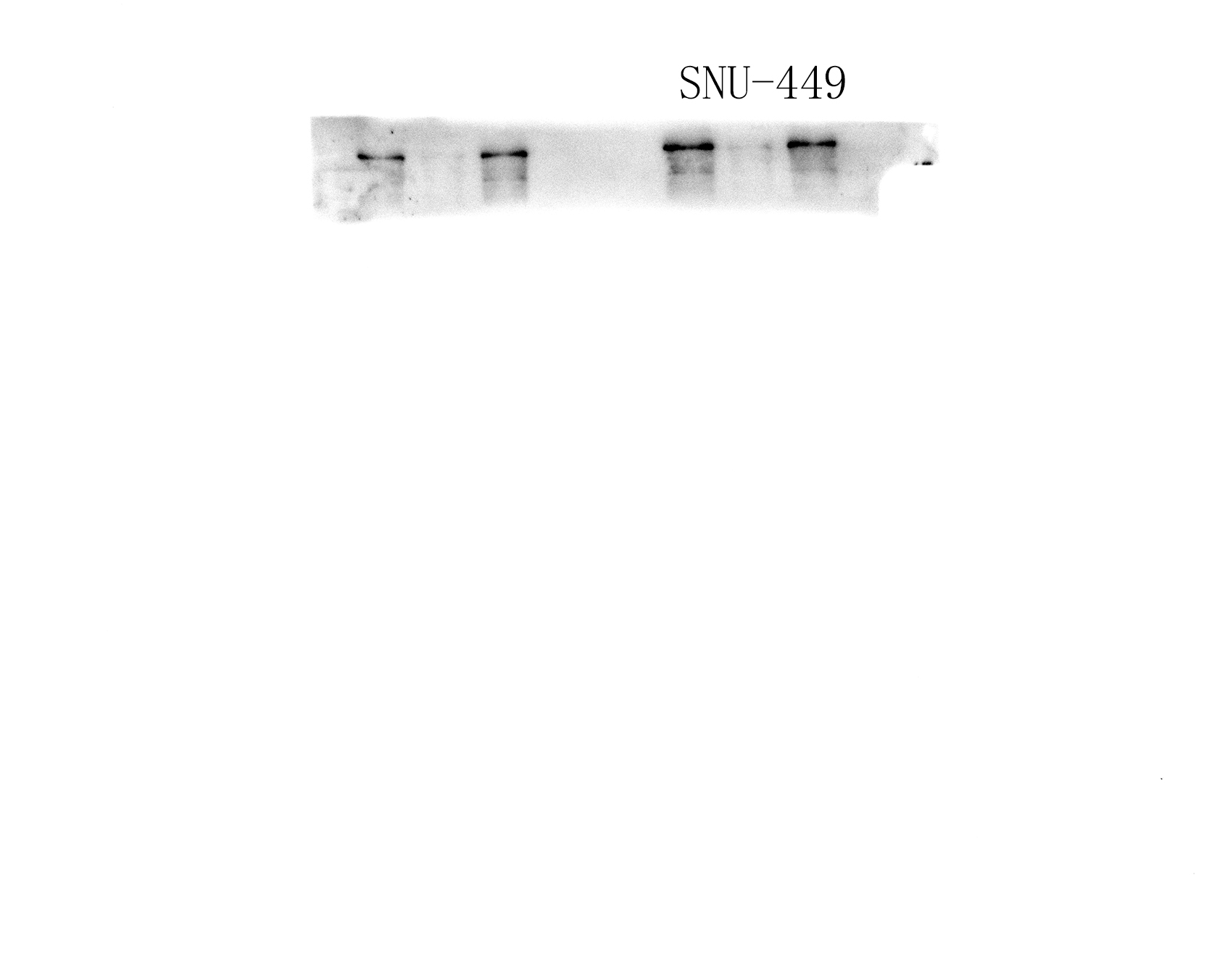

Supplement: Supplementary file 1 [file DataSheet1.zip › Figure 8 Excel/E WB/N-cadherin 3.jpg]

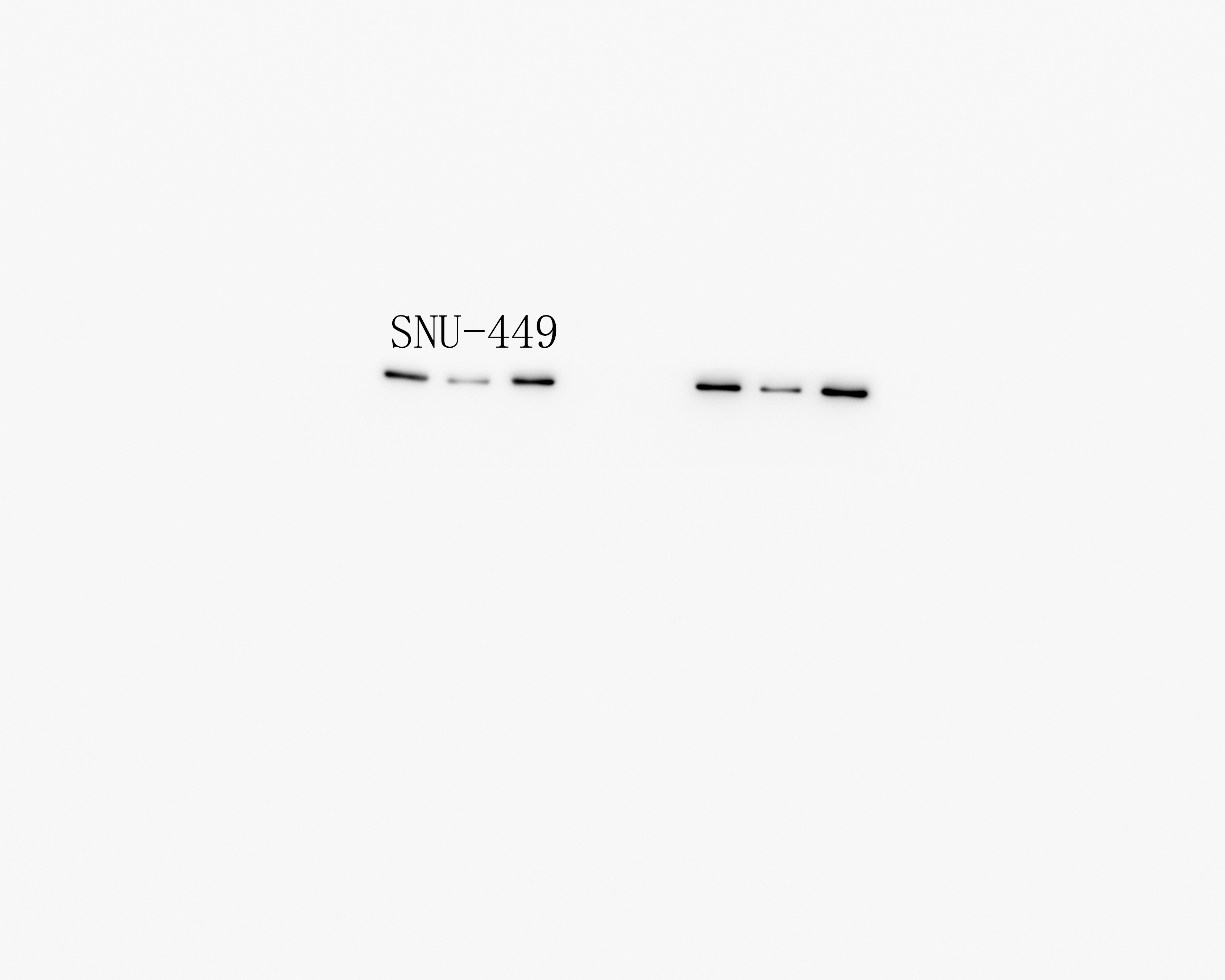

Supplement: Supplementary file 1 [file DataSheet1.zip › Figure 8 Excel/E WB/Vimentin 3.jpg]
